# Synthesis and Biological Evaluation of TDP1 Inhibitors Based on Coumarin and Monoterpenoid Fragments Conjoined by Heterocyclic Moieties

**DOI:** 10.3390/ijms27146421

**Published:** 2026-07-19

**Authors:** Dmitriy Tsypyshev, Tatyana Khomenko, Tatyana Kornienko, Alexandra Zakharenko, Nina Komarova, Vyacheslav Krasnov, Natalya Soldatova, Pavel Postnikov, Suat Sari, Konstantin Volcho, Olga Lavrik, Nariman Salakhutdinov

**Affiliations:** 1 Novosibirsk Institute of Organic Chemistry, Siberian Branch of the Russian Academy of Sciences (SB RAS), Novosibirsk 630090, Russia; tsypyshev@nioch.nsc.ru (D.T.); chomenko@nioch.nsc.ru (T.K.); komar@nioch.nsc.ru (N.K.); krasnov@nioch.nsc.ru (V.K.); postnikov@tpu.ru (P.P.); anvar@nioch.nsc.ru (N.S.); 2 Institute of Chemical Biology and Fundamental Medicine, Siberian Branch of the Russian Academy of Sciences (SB RAS), Novosibirsk 630090, Russia; t.kornienko1995@gmail.com (T.K.); zakharenko@1bio.ru (A.Z.); lavrik@niboch.nsc.ru (O.L.); 3 Tomsk Polytechnic University, Tomsk 634050, Russia; olsonata@tpu.ru; 4 Department of Pharmaceutical Chemistry, Faculty of Pharmacy, Hacettepe University, Ankara 06230, Turkey; suat.sari@hacettepe.edu.tr

**Keywords:** coumarin, monoterpene, heterocycles, enzyme inhibition, DNA repair, cancer, synergy

## Abstract

Tyrosyl-DNA phosphodiesterase 1 (TDP1) represents a compelling pharmacological target for the development of agents designed to circumvent tumor resistance to topoisomerase 1 (TOP1) inhibitors, a major class of clinically relevant antineoplastic drugs. This paper describes the design and synthesis of novel hybrid TDP1 inhibitors combining coumarin and monoterpene moieties via rigid isoxazole and 1,2,3-triazole heterocyclic linkers. The synthesis was accomplished via [3 + 2] cycloaddition of nitrile oxides to alkynes and copper-catalyzed click chemistry. Biological tests have demonstrated the crucial role of linker nature in the activity of the compounds. Isoxazole-linked conjugates showed strong inhibitory effects on TDP1, with IC_50_ values in the submicromolar to low micromolar range (0.8–3.2 μM). Overall, these values slightly surpassed those of the triazole-linked analogues, whose IC_50_ values ranged from 1.1 to 23.3 μM. At noncytotoxic doses, compounds **26e** and **16b** enhanced the sensitivity of human cervical cancer (HeLa) cells to the antitumor agent topotecan, a TOP1 inhibitor, thereby supporting the promise of this structural class as components of combination chemotherapy.

## 1. Introduction

Topoisomerase I (TOP1) inhibition is a well-established chemotherapy strategy for a number of tumors, including colorectal cancer, ovarian cancer, and small-cell lung cancer [1,2]. TOP1 is a critical nuclear enzyme that eliminates DNA topological stress (supercoiling) that occurs during replication and transcription [3]. Clinically relevant camptothecin derivatives (topotecan and irinotecan) act as TOP1 poisons. Their mechanism of action involves stabilizing the enzyme’s covalent complex with DNA (TOP1cc), which prevents the religation of dissected strands. The accumulation of stabilized TOP1cc complexes leads to lethal DNA damage and subsequent tumor cell death [2].

**Figure 1:**
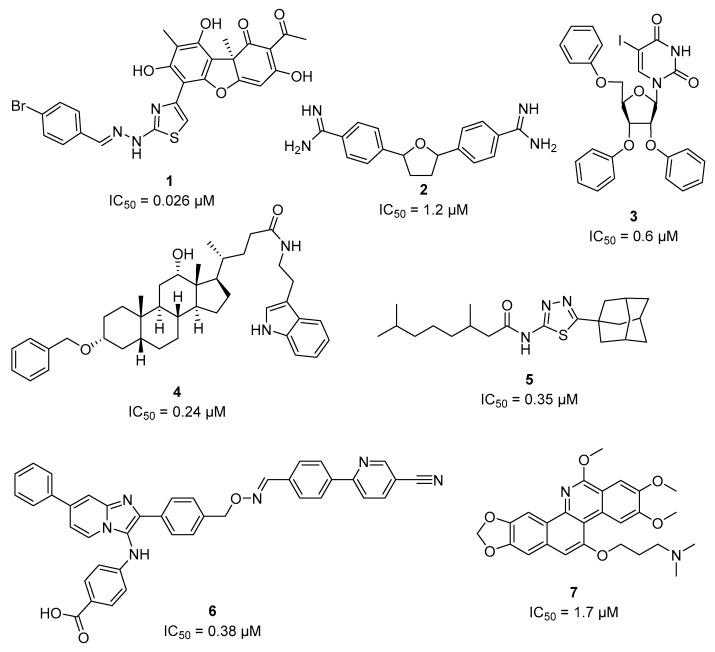
Some known TDP1 inhibitors and their IC_50_ values.

However, the clinical efficacy of camptothecin therapy is often limited by the evolvement of drug resistance. One of the key factors in this resistance is considered to be the activity of the repair enzyme tyrosyl DNA phosphodiesterase 1 (TDP1) [4,5]. TDP1 belongs to the phospholipase D (PLD) superfamily and plays a central role in the repair of DNA damage blocked at the 3′ end by protein adducts or other chemical groups [6,7]. The main function of the enzyme is to hydrolyze the phosphodiester bond between the tyrosine residue of the active site of TOP1 and the 3′-phosphate of DNA. This reaction ensures the removal of the covalently “stuck” TOP1 enzyme, allowing access for subsequent stages of repair [3]. Thus, inhibition of TDP1 prevents the elimination of cytotoxic damage induced by TOP1 poisons and is capable of sensitizing tumor cells to chemotherapy [7,8]. Some known TDP1 inhibitors are shown in Figure 1.

To date, numerous classes of TDP1 inhibitors of natural and synthetic origin have been described, such as usnic acid derivatives (**1**) [9,10,11,12], demonstrating activity in the nanomolar range; furamidine (**2**) [13]; nucleoside derivatives (**3**) [14,15]; deoxycholic acid derivatives (**4**) [16,17]. Also of considerable interest are hybrid structures based on adamantane or thiazolidine-2,4-dione conjugated with monoterpenes [18,19,20], for example, benzophenanthridine derivatives (**5**) [21,22,23]; imidazopyridine derivatives (**6**) [24,25,26]; natural compounds and their analogs (**7**) [27,28,29]; substrate-binding inhibitors [30]; substrate-like inhibitors [31]; other synthetic small molecules [32,33,34,35,36,37,38]; and dual Top1-TDP1 inhibitors [39,40] showing high inhibitory activity.

**Figure 2:**
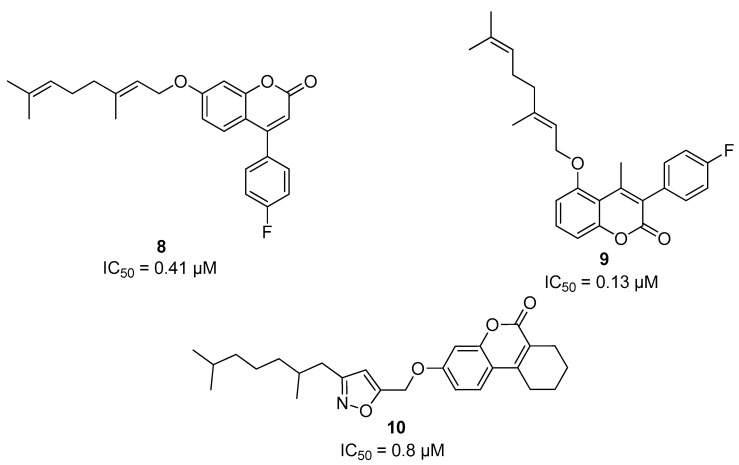
Some known TDP1 inhibitors containing the coumarin moiety and their IC_50_ values.

A special place among potential inhibitors is occupied by hybrid structures combining fragments of monoterpenes and coumarins [41,42,43,44], some of which are shown in Figure 2. Coumarins and terpenoids are accessible secondary plant metabolites characterized by a broad spectrum of biological activity and, as a rule, favorable toxicity profiles. It has previously been shown that the combination of terpene and coumarin pharmacophores allows the production of highly active TDP1 inhibitors, such as **8**–**10**, with IC_50_ values in the nanomolar and submicromolar ranges.

In vivo studies have also been performed for some compounds to reveal the mechanisms of action and possible synergetic effects with respect to TOP1 inhibitors [12,15,43]. For example, compound **1** demonstrated [12] low toxicity together with significant synergism with topotecan. While low doses of topotecan alone were ineffective against the primary tumor, the combination with **1** resulted in a 57.6% reduction in tumor weight and an order-of-magnitude reduction in the number of metastases. Furthermore, the inhibitor did not worsen the side effects of chemotherapy, such as weight loss or internal organ pathologies.

Combination therapy with compound **8** and topotecan [43] exhibited high antitumor efficacy in mice. Combined use of these compounds at optimal doses suppresses tumor growth and reduces the number of cancer cells in ascites. In addition, this combination increases the survival time of diseased animals (by 42% compared to no treatment and by 26% compared to topotecan alone), while the individual drugs do not lead to statistically significant improvements in survival. The efficacy of compound **8** was also further confirmed in another in vivo experiment: when mice were administered a topotecan-and-compound **8** mixture intraperitoneally (0.25 mg/kg of topotecan, 10–160 mg/kg of **8**), the mutagenic effect of topotecan was enhanced with doses of 8 ranging from 20 to 160 mg/kg compared to the control (topotecan without **8**). The effect was not dose-dependent: the increase in activity (2.1x to 2.6x) was similar for all active doses of **8** [45]. Furthermore, recently developed phenanthridine derivatives acting as dual TOP1/TDP1 inhibitors have also demonstrated high in vivo efficacy, exhibiting dose-dependent tumor growth suppression in an A549 non-small-cell lung cancer xenograft mouse model while causing less body weight loss compared to that observed in the topotecan control group [46].

It has been shown that the introduction of a rigid heterocyclic linker between the monoterpene and adamantane moieties has a positive effect on inhibitory activity against TDP1 [18]. Recently, we found that the introduction of an isoxazole linker between the coumarin and monoterpene moieties, as carried out using compound **10** as an example, allows one to retain the inhibitory activity against TDP1 [44]. We hypothesize that the inclusion of a rigid heterocyclic fragment, such as isoxazole or triazole, can facilitate the optimization of the spatial organization of molecules (limiting conformational mobility), enhance interaction with the active site of the enzyme via additional hydrogen bonds or stacking interactions, and improve the physicochemical properties of the compounds. Thus, our contribution aims to elucidate the role of rigid heterocyclic linkers in the activity of TDP1 inhibitors in combination therapy with topotecan and propose a novel family of potential anticancer drugs. To achieve this, we sufficiently expanded the library of isoxazole-linked coumarin–monoterpene conjugates by varying the terpene moiety and coumarin backbone and introducing triazole-linked monoterpene–coumarin conjugates into the study.

## 2. Results and Discussion

### 2.1. Chemistry

#### 2.1.1. Synthesis of Monoterpene–Coumarin Conjugates Containing an Isoxazole Moiety

Many methods for the preparation of 3,5-disubstituted isoxazoles are known, among which the dominant ones are cyclocondensation reactions of hydroxylamine with 1,3-dicarbonyl compounds or their equivalents [47], as well as 1,3-dipolar cycloaddition reactions of nitrile oxides with alkynes [48]. However, the synthesis of such compounds is often complicated by the formation of difficult-to-separate mixtures of regioisomers (3,5- and 5,3-substituted products), which requires careful use of specific conditions [47,49]. Furthermore, many existing protocols impose significant limitations on the substrates used due to low tolerance to functional groups [50].

We used a method for the in situ generation of nitrile oxides by oxidation of aldoximes with reagents based on hypervalent iodine [51,52]. Despite the inherent risk of side dimerization with furoxans associated with nitrile oxides [53], this approach was chosen due to its “metal-free” nature, which avoids contamination of the product with traces of transition metals. Furthermore, the use of hypervalent iodine ensures the reaction is carried out under mild conditions with high, often complete, regioselectivity in favor of the target 3,5-isomer [51].

**Scheme 1:**
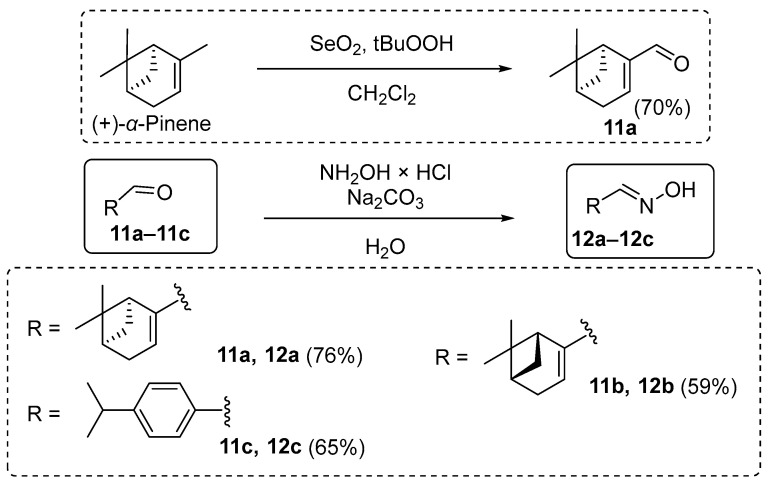
Synthesis of monoterpene oximes **12a**–**12c**.

For the synthesis of the terpene oximes, the corresponding commercially available aldehydes **11b** and **11c** were used as starting compounds. (+)-Myrtenal **11a** was prepared according to a previously described procedure by reacting (+)-*α*-pinene with selenium(IV) oxide in a solution of tert-butyl hydroperoxide in methylene chloride [54]. Aldehydes **11a**–**c** were reacted with hydroxylamine hydrochloride, resulting in *E*/*Z* mixtures of oximes **12a**–**c** in yields ranging from 59% to 76% [55] (Scheme 1).

Before synthesizing the target isoxazoles, it is necessary to select conditions that are most favorable for the synthesis of the target product. This requires an understanding of the rate of nitrile oxide formation from the oxime and the rate of nitrile oxide dimerization. Ideally, the nitrile oxide molecule should be surrounded by an excess of a substrate with a terminal triple bond, so the oxime should be added in small portions, with intervals between additions, allowing for complete conversion.

**Scheme 2:**
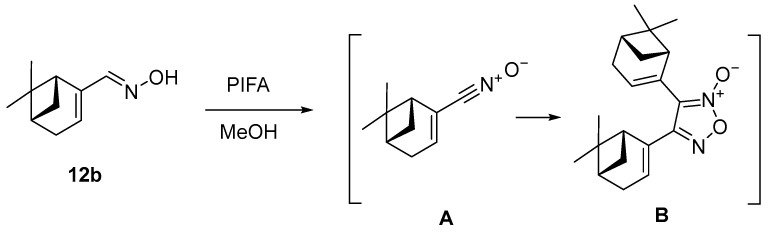
Principal scheme of the dimerization of **12b**.

To study the kinetics of the conversion of (−)-myrtenal oxime **12b** into nitrile oxide **A**, and then into furoxan **B**, by analogy with [53], upon exposure to PIFA in methanol (Scheme 2), the reaction was performed in an NMR tube. ^1^H NMR spectra were recorded every three minutes to monitor the progress of the transformations in the medium.

As detailed in the Supporting Information (Figure S47), ^1^H NMR monitoring of the reaction kinetics allowed us to compare the rates of reagent consumption and product formation. The oxidation of oxime by PIFA proceeds rapidly; the starting materials are consumed synchronously and quantitatively, achieving complete conversion within approximately 15 min. In contrast, the subsequent dimerization of the intermediate nitrile oxide into the furoxan product occurs at a slightly slower rate. This is evidenced by the olefinic proton of the terpene fragment, which begins to reflect the asymmetry of the newly formed furoxan ring at 15 min and reaches a stable state at 24 min, indicating the completion of the transformation. Comparing these rates confirms that the in situ generation of the nitrile oxide outpaces its dimerization. Therefore, to minimize dimerization and favor the target cycloaddition, we decided that the oxime should be introduced to the reaction mixture in small portions in 30 min intervals. This timeframe guarantees that all intermediate nitrile oxide is fully consumed before the next portion of oxime is added.

**Scheme 3:**
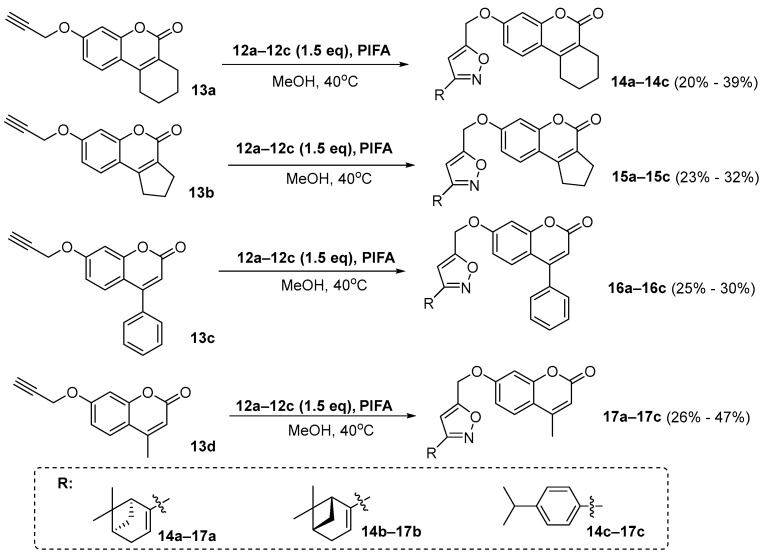
Synthesis of isoxazole-linked terpene–coumarin conjugates **14a**–**c**, **15a**–**c**, **16a**–**c**, and **17a**–**c**.

Through this procedure, isoxazole-linked conjugates **14a**–**c**, **15a**–**c**, **16a**–**c**, and **17a**–**c** were synthesized from propargyl ethers **13a**–**d** and oximes **12a**–**c** by in situ generation of nitrile oxides using PIFA [51], according to Scheme 3. Propargyl ethers **13a**–**d** were prepared according to a previously published procedure [54].

To increase the conversion of the starting propargyl ethers of coumarins, oxime was added in a 1.5-fold excess. However, adding oxime in a three-fold excess did not increase the yield, nor did heating above 40 °C. A number of different hypervalent iodine-based oxidants were also tested, including PIDA, PIFA, and IBX, and in this series, the highest yields were obtained using PIFA.

**Scheme 4:**
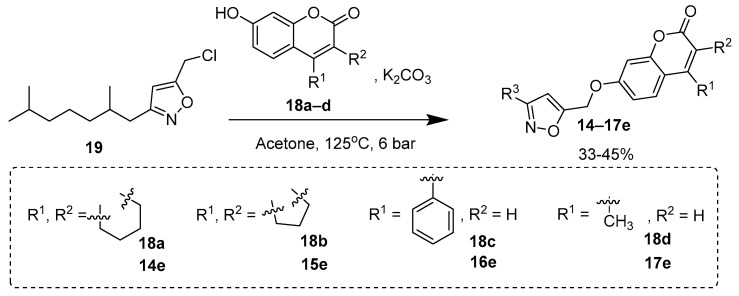
Previously performed synthesis of **14**–**17e**.

Conjugates **14**–**17e** were prepared previously [44,45] by reacting the corresponding hydroxycoumarins **18a**–**d** with 5-chloromethylisoxazole **19** in an Anton Paar Monowave 50 reactor in acetone and in the presence of potassium carbonate (Scheme 4).

#### 2.1.2. Synthesis of New Monoterpene–Coumarin Conjugates Containing a Triazole Moiety

**Scheme 5:**
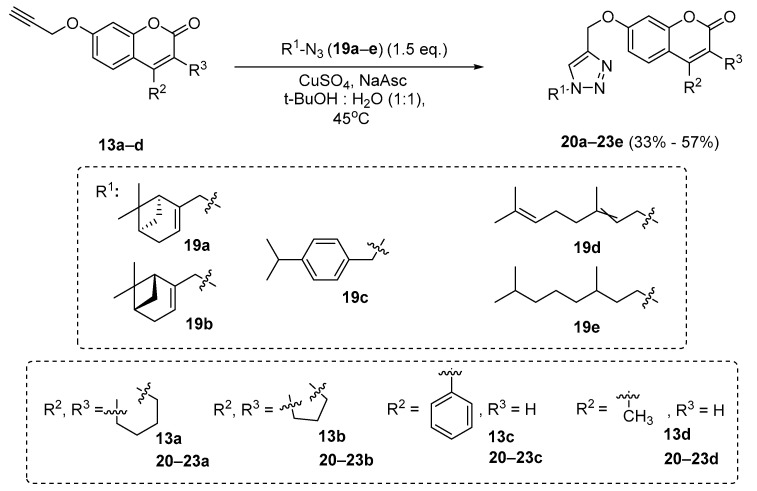
Previously performed synthesis of **20a**–**23e**.

A method for synthesizing triazole-containing conjugates was developed by us previously in [54]. To obtain new conjugates of this series, we employed the same approach via [3 + 2] azide-alkyne cycloaddition. Compounds **20a**–**23e** were also involved in the biological tests described below (Scheme 5).

**Scheme 6:**
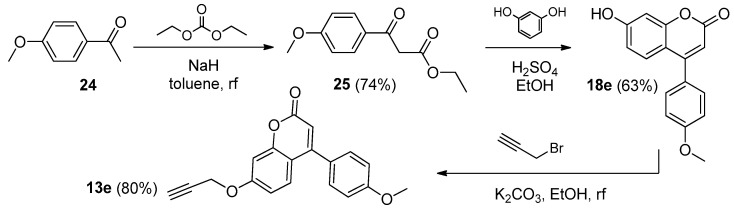
Synthesis of 4-methoxyphenyl-7-propargyloxycoumarin **13e**.

In order to synthesize a library of triazole-containing coumarin–monoterpene conjugates containing methoxy or hydroxyl groups in the coumarin and terpene parts of the molecules, propargyl ester **13e** containing a *p*-methoxyphenyl group in the coumarin skeleton was synthesized (Scheme 6).

*β*-Ketoester **25** was prepared according to the method outlined in [56] by reacting 4-methoxyacetophenone **24** with diethyl carbonate and sodium hydride via reflux in toluene, which led to intermediate product **25** in a 74% yield (Scheme 6). Ethyl 3-(4-methoxyphenyl)-3-oxopropanoate **25** was then introduced into a Pechmann condensation with resorcinol [57], leading to 7-hydroxycoumarin **18e** in a 63% yield, which was alkylated with propargyl bromide [58]. The target ether **13e** was isolated in an 80% yield.

**Scheme 7:**
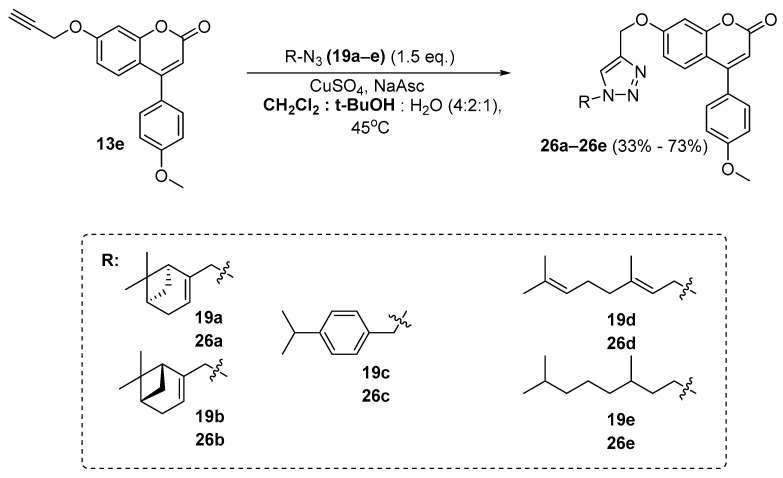
Synthesis of triazole-containing compounds **26a**–**e**.

Triazole-containing derivatives **26a**–**e** were prepared from compound **13e** (Scheme 7) using a modified azide–alkyne cycloaddition method [54]. To increase solubility, the commonly used solvent mixture (tert-butyl alcohol and water) was modified: an excess amount of methylene chloride and tert-butyl alcohol was added to the mixture until a homogeneous solvent mixture was achieved. Conjugates **26a**–**26e** were obtained in yields ranging from 33 to 73% (Scheme 7).

### 2.2. Biology

The ability of the synthesized compounds to inhibit the TDP1 enzyme was assessed using a method described previously [59,60]. A 16-mer single-stranded DNA containing a fluorophore at the 5′ end (FAM) and a quencher at the 3′ end (BHQ1) was used as a substrate for measuring TDP1 activity. The inhibitory activity of the compounds was assessed based on their ability to suppress the activity of TDP1, that is, to prevent the cleavage of the BHQ1 and, thus, prevent an increase in fluorescence intensity.

To facilitate the assessment of the “structure of the synthesized compounds–TDP1 inhibitory activity” relationship, the biological test results are summarized in “heat maps” (Table 1 and Table 2), in which the IC_50_ inhibitory concentration values are plotted at the intersection of the data axes containing the compound fragment types.

Isoxazole-containing conjugates exhibited comparable activity, with the exception of compound **15c**, with IC_50_ values ranging from 0.8 to 3.1 µM. A slight increase in activity for 4-arylcoumarin derivatives and a slight decrease in activity for 4-methylcoumarins were observed. Compounds **14e**, **15a**, **16a**, and **16b** exhibited the highest activity, with IC_50_ values less than 1 µM. The absolute configuration of the pinene moiety did not significantly affect the inhibitory activity of the compounds.

Among the emerging patterns in the comparison of the activity of triazole-containing compounds, it should be noted that 4-methyl-substituted coumarins exhibit lower activity, while coumarins with an aromatic substituent at the 4 position exhibit comparatively higher activity. Furthermore, derivatives with a 3,7-dimethyloctane moiety also exhibit enhanced inhibitory activity. No clear general pattern in the influence of the absolute configuration of the pinene moiety on the inhibitory concentration was revealed.

Among isoxazole-linked coumarin–terpene conjugates, compounds **14e**, **15a**, **16a**, and **16b** had the highest TDP1 inhibitory activity (with IC_50_ < 1.0 µM); among triazoles, there were no compounds with submicromolar activity. Triazoles that demonstrated acceptable inhibitory activity included derivatives **26a**–**e** (IC_50_ 1.5 to 5 µM), **22a**–**e** (IC_50_ 1.1 to 3.2 µM), and **20**–**23e**, **26e** (IC_50_ 1.5 to 5 µM).

Based on the information obtained, the most promising compounds for further targeted development of new effective TDP1 inhibitors are conjugates containing aromatic moieties in the coumarin backbone, linked via a 3,5-isoxazole fragment to acyclic fragments of the terpene moiety of the molecule.

### 2.3. In Vitro Studies—Toxicity and Synergistic Effect in Topotecan Therapy

**Figure 3:**
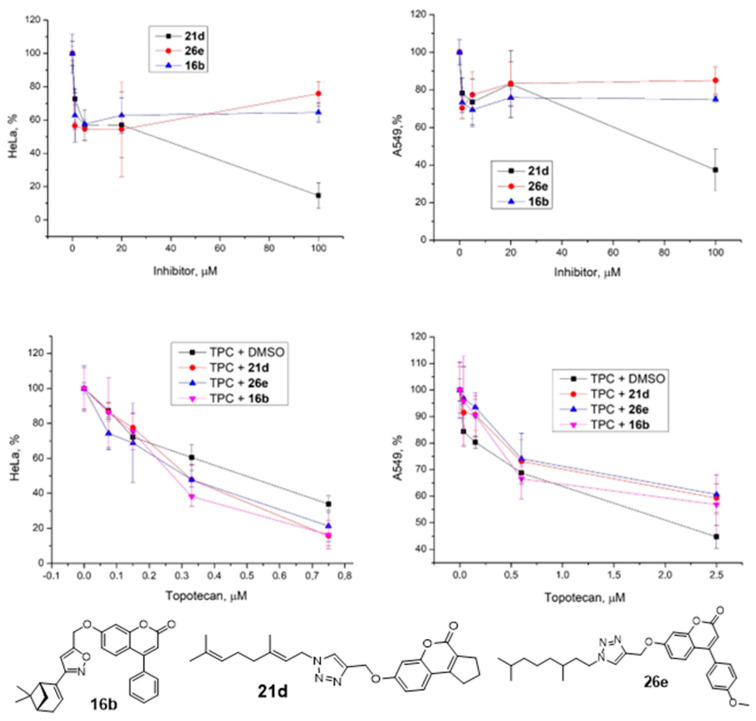
Graphs of the proportion of surviving HeLa and A549 cells versus the intrinsic concentration of the compound, as well as the concentration of topotecan, or the “topotecan + monoterpene–coumarin conjugate” mixture.

To study the effects of inhibitors on cell lines and the cytotoxic effect of topotecan, the cytotoxicity of compounds **21d**, **26e**, and **16b** was examined. Three independent cytotoxicity tests were then conducted with each inhibitor in combination with topotecan (Figure 3, Table 3). For cells treated with the monoterpene–coumarin derivative alone, the values in the control wells treated with 1% DMSO were taken to be 100% viability. For cells treated with the combination of the drugs, the viability of cells treated with the corresponding TDP1 inhibitor alone was taken to be 100%.

Compound **21d** exhibited intrinsic cytotoxicity towards HeLa and A549 cells, while compounds **26e** and **16b** did not exhibit cytotoxicity towards the studied cell lines. However, a synergistic effect was observed during combination therapy with topotecan on HeLa cells using compounds **26e** and **16b**. No synergistic effect was observed for the A549 cell line. Variable synergistic effects of TDP1 inhibitors in topotecan therapy applied to different cell lines have been observed before [61]; this effect was revealed in a family of heterocycle-linked monoterpene–coumarin conjugates, potentially indicating that there is a hidden selectivity pathway, which demands further study.

### 2.4. Molecular Modelling of Monoterpene Derivatives with Human TDP1 Structures

#### 2.4.1. Validation of Docking and the Crystallographic Structure

Many crystallographic structures of human TDP1 have been deposited in Protein Data Bank so far. In order to evaluate the binding poses of the title compounds at the TDP1 active site, we evaluated these structures and looked for the structure with the best relationship between the TDP1-inhibitory effects of the compounds and their docking scores. We initially identified crystallographic TDP1 structures with a co-crystallized non-covalent small-molecule inhibitor in the catalytic site and with a resolution ≤ 2.0 Å. Then, we performed a redocking validation for these 27 structures, in which the co-crystallized inhibitor was redocked to the catalytic site of each receptor to compare the resulting pose with the co-crystallized one by calculating the RMSD (root-mean-square deviation). Six structures with RMSD values of co-crystallized inhibitors ≤2.0 Å were identified (Table S1), and they were later used for docking of the title compounds. The docking scores obtained from these structures showed almost no correlation with their TDP1 IC_50_ values (Table S2). Then, the three top-scoring poses of each compound from molecular docking were selected, and their ΔG values in complex with a receptor were calculated using the MM-GBSA method, which yielded the best R^2^ value (0.1723) for 6DJE (Table S2). Although such R^2^ values may be thought to be generally too low to build a strong correlation, they might be helpful in the case of docking given the following considerations: (i) Molecular docking predicts the fitness of a molecule inside a receptor pocket and the physical affinity of the two, while IC_50_ shows inhibition of an entire enzymatic process, and these two factors may be poorly related in real time. (ii) A highly predictive molecular docking process can be expected to enrich active molecules among inactive molecules; given that most of the title compounds had IC_50_ values in a narrow range, which can easily be interpreted as a “potent inhibitory effect”, a molecular docking procedure for sorting such compounds in the same order as their biological activity would not be prudent. Therefore, 6DJE was considered a reliable structure with which to evaluate the binding poses of the compounds (see Table S3 for full scores).

#### 2.4.2. Predicted Binding of the Title Compounds at the TDP1 Active Site

**Figure 4:**
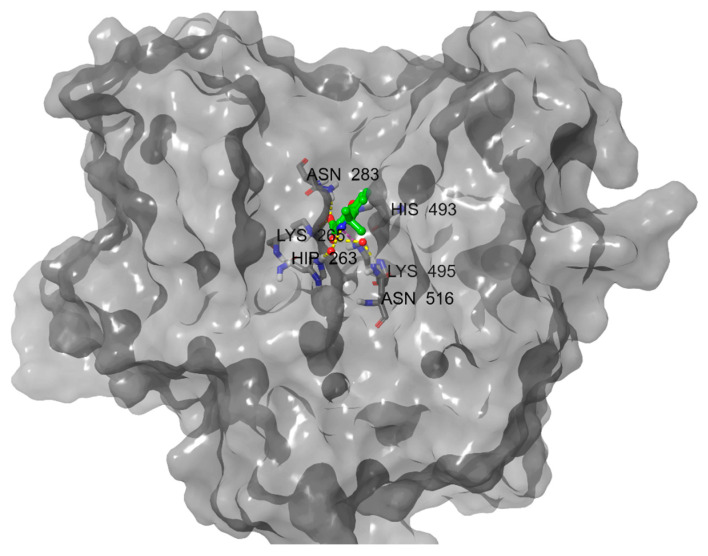
Human TDP1 crystallographic structure in complex with an inhibitor. Both catalytic HNK triads are represented via grey sticks, and the residues are tagged, while the inhibitor is shown via a green stick-and-ball representation. Protein surface is rendered.

The human TDP1 catalytic site is a buried pocket in the middle of a ca. 33 Å substrate binding groove, which is composed of a wide half that widens towards the end accommodating a peptide moiety of the substrate and a narrow half for DNA binding. The catalytic process is governed by a catalytic double-HNK motif, i.e., histidine (His263, His493), asparagine (Asn283, Asn516), and lysine (Lys265, Lys495), with each couple located on either side of the gorge lining the catalytic pocket (Figure 4). The inhibitors of TDP1 described so far have been reported to interact with these residues, as well as others that join catalytic process by substrate stabilization, such as Tyr204 and Ser518 [62,63].

**Figure 5:**
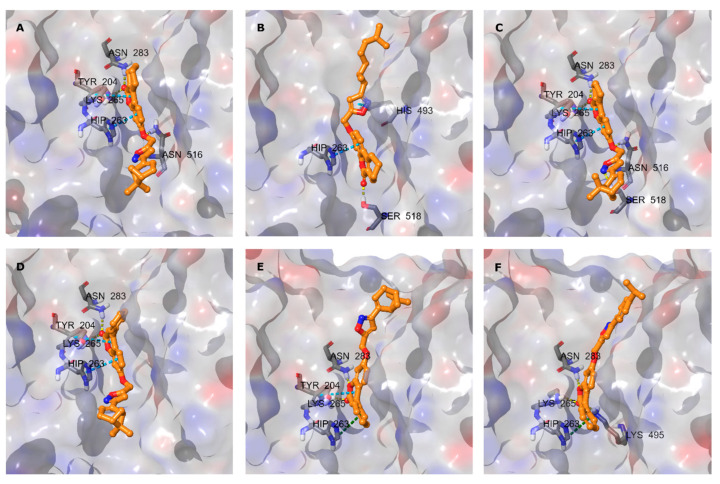
Predicted binding modes of **14b** (**A**), **14d** (**B**), **15a** (**C**), **16a** (**D**), **16b** (**E**), and **16c** (**F**) at the human TDP1 active site. Ligands are shown using an orange stick-and-ball representation, amino acid residues are given as grey sticks, and electrostatic interactions are represented using colorful dashed lines. Protein surface is rendered according to electrostatic potential.

The title compounds were predicted to have two possible binding modes, in which the monoterpene moiety occupied either the peptide-binding site or DNA-binding site of the groove (Figure 5). However, for both binding modes, there was one thing in common: the coumarin (1,2-benzopyrone) ring was inside the catalytic pocket and engaging in electrostatic interactions with the catalytic residues. The oxazole/triazole ring and the etheric oxygen between the rings contributed to these interactions, which were enabled through electron-donating groups and the π systems of the rings. For example, **14b** was in contact with one of the HNK motifs and Tyr204 via the 1,2-benzopyrone ring and with Asn516 via the etheric oxygen while its monoterpene moiety occupied the DNA-binding cleft (Figure 5A). In the case of **14d**, the aromatic rings engaged with His263, His493, and Ser518, while the isooctyl moiety stretched over the peptide-binding cleft (Figure 5B). Compounds **15a** and **16a** (Figure 5C,D) were predicted to have binding poses similar to that of **14b**, while **16b** and **16c** were predicted to have binding poses similar to that of **14d** (Figure 5E,F), in which catalytic and key residues were involved (see Figure S49 for details). These results show that the compounds may assume alternating binding poses, but the 1,2-benzopyrone core is responsible for blocking the catalytic pocket through electrostatic contacts, hence the inhibitory effect, while the monoterpene moiety mainly stabilizes this blockage through effective hydrophobic contacts in the groove, either in the peptide- or DNA-binding part. Detailed 2D interaction diagrams and the corresponding interacting residues are provided in Figure S49 and Table S4, respectively.

**Figure 6:**
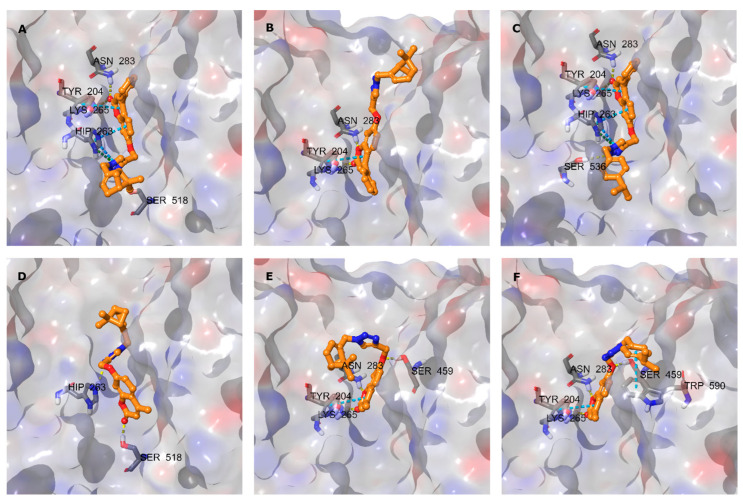
Predicted binding modes of **22a** (**A**), **22b** (**B**), **22c** (**C**)**, 23a** (**D**), **23b** (**E**), and **23c** (**F**) at the human TDP1 active site. Ligands are shown via orange stick-and-ball representation, amino acid residues are depicted as grey sticks, and electrostatic interactions are represented as colorful dashed lines. Protein surface is rendered according to electrostatic potential.

When compared with their counterparts in the **22** and **23** series compounds, as they showed markedly higher IC_50_ values than the rest of the collection, the triazole derivatives were predicted to have poses diverging from the pattern described above. **22a**–**c** were predicted to have poses following the above-mentioned trend, where the coumarin ring was effectively inside the catalytic pocket and the monoterpene moiety was in the groove, either in the narrow or wide section (Figure 6A–C). However, 1,2-benzopyrone of **23a**–**c** was dragged partly outside of the catalytic pocket, and, in the case of **23b** and **23c**, the monoterpene moiety was outside the groove (Figure 6D–F), probably reducing the efficacy of binding (see Figure S50 for details on the predicted binding interactions).

The configuration of the *α*-pinene moiety had little or no effect on the binding orientations of the compounds, a finding that is in compliance with the bioactivity data. Both the (+)-*α*-pinene and (−)-*α*-pinene moieties of the most active derivatives fit in the DNA-binding cleft in a similar way, while these moieties in the derivatives with much higher IC_50_ values occupied either cleft independently of the configuration.

### 2.5. Comparative Lipophilicity of the Obtained Compounds

To assess the effect of the introduced heterocyclic linkers on the lipophilicity of the adducts, the retention times of three compound groups were determined: **23b**, **14b**, and their ether analogue without a heterocyclic linker; **26b**, **17b**, and their ether analogue; and **24a**, **15a**, and their ether analogue. This was done by HPLC using an approach similar to that described in [17] (ProntoSIL-120-5-C18, C18 AQ column, the sorbent of which is silica gel modified with C-18 hydrocarbon fragments). Based on the properties of reversed-phase chromatography, hydrophilic compounds appear on the chromatogram earlier (their retention times are shorter) than lipophilic ones. Indeed, the retention times of compounds **23b**, **26b** and **24a** containing a triazole linker are shorter than those of isoxazole-containing conjugates **14b**, **17b** and **15a** and shorter still than those of related compounds that do not contain a heterocyclic linker (Figure S51), suggesting that inclusion of a heterocyclic linker, triazole in particular, reduces the lipophilicity of the biologically active compound from the monoterpene–coumarin family, which might be beneficial to their biological activity in vivo.

### 2.6. Drug-likeness

The drug-likeness of the title compounds was evaluated using a set of descriptors (logP, molecular weight, topological polar surface area, logS, ratio of sp^3^-hybridized carbons, and number of rotatable bonds, accounting for lipophilicity, size, polarity, aqueous solubility, unsaturation, and flexibility, respectively) and the bioavailability radar technique developed by Daina et al. utilizing these descriptors generated via the SwissADME web application [64].

Although most compounds complied with the majority of the descriptors, some of the modifications caused the compounds to lie outside the plot (Figure S48). For example, citral and 3,7-dimethyloctane moieties led to higher logP values and more rotatable bonds for some compounds such as **16e**, **26d**, **26e**, **20e**, and **22e**, which indicates higher lipid solubility and flexibility than required. In the case of cymene, which includes a benzene ring, the ratio of the sp^3^-hybridized carbons over all carbons was too low for compounds such as **16c**, **26c**, and **22c**, leading to excessively high unsaturation, which is considered a nondrug-like property [64].

## 3. Materials and Methods

Spectral and analytical studies were carried out at the Chemical Service Center for Collective Use of the Scientific Research Institute of Organic Chemistry SB RAS. ^1^H and ^13^C NMR spectra of the obtained compounds were recorded on AV-200, AV-400, DRX-500, AV-300, and AV-600 spectrometers (Billerica, MA, USA) in a solution of CDCl_3_, MeOH, or DMSO-d_6_. Solvent signals were used as an internal standard. The structure was established based on an analysis of ^1^H and ^13^C NMR spectra using, in some cases, two-dimensional homonuclear correlation spectra (^1^H-^1^H COSY, ^1^H-^1^H NOESY) with the involvement of two-dimensional heteronuclear correlation spectroscopy ^13^C-^1^H for direct and long-range spin–spin interaction constants (^13^C-^1^H HSQC, ^13^C-^1^H HMBC). Chloroform (*δ*H 7.26 ppm, *δ*C 76.16 ppm) or DMSO (*δ*H 2.50 ppm, *δ*C 39.52 ppm) signals were used as internal standards. The atom numbering of the compounds is given for assignment of NMR spectral signals and does not coincide with the nomenclature. The corresponding NMR spectra are provided in Figures S1–S28.

The reaction products were isolated via column chromatography using silica gel (Merck, Darmstadt, Germany, 60–200 mesh, Macherey-Nagel GmbH & Co. KG, Düren, Germany), with the eluents being hexane-ethyl acetate and benzene-chloroform. The solvents used in this study were purified before use using standard methods. Accurate molecular ion masses were determined using a Thermo Scientific DFS High-Resolution GC/MS (Waltham, MA, USA), Double-Focusing Sector Mass Spectrometer, DFS High-Resolution GC/MS) in full-scan mode (15–500 *m*/*z*, 70 eV electron impact ionization, direct sample injection). The corresponding HRMS spectra are provided in Figures S29–S46. Specific rotation ${\left[\alpha \right]}_{589}^{23.4}$ values were determined on a PolAAr 3005 spectrometer (Billerica, MA, USA)) using chloroform as solvent. Melting points were determined on a METTLER TOLEDO FP900 melting point apparatus.

### 3.1. Synthesis of Compounds

#### 3.1.1. Synthesis of Oximes **12a**–**c**

A solution of sodium hydrogen carbonate (20 mmol) in 15 mL of water was added to a vigorously stirred solution of hydroxylamine hydrochloride (20 mmol) in 10 mL of water containing the corresponding aldehyde (**11a**–**c**) (16 mmol). The mixture was stirred and heated for 5 h. The resulting organic layer was separated, and the aqueous phase was further extracted with ether. The organic phases were combined, dried over sodium sulfate, and evaporated. The yields of compounds **12a**–**c** were 76%, 59%, and 65%, respectively. The spectral data correspond to data from the literature [65,66,67].

#### 3.1.2. General Procedure for the Synthesis of Isoxazoles **14a**–**17c**

Coumarin ester (0.59 mmol) was added to a solution of PIFA oxidizer (0.86 mmol) in 10 mL of methanol while being heated to 40 °C. After 10 min, a solution of the corresponding oxime (0.86 mmol) in 5 mL of methanol was added in small portions every half hour for 3 h. After the addition was complete, the mixture was stirred at 40 °C for another hour and then left overnight. The reaction mixture was extracted with ethyl acetate; the organic phase was washed with water, dried over sodium sulfate, filtered, and evaporated. The resulting product was purified by column chromatography on SiO_2_ using a hexane–chloroform–ethyl acetate mixture, where the concentrations of hexane and chloroform were equal, and the EtOAc content was gradually increased from 0% to 5%.

**Figure 7:**
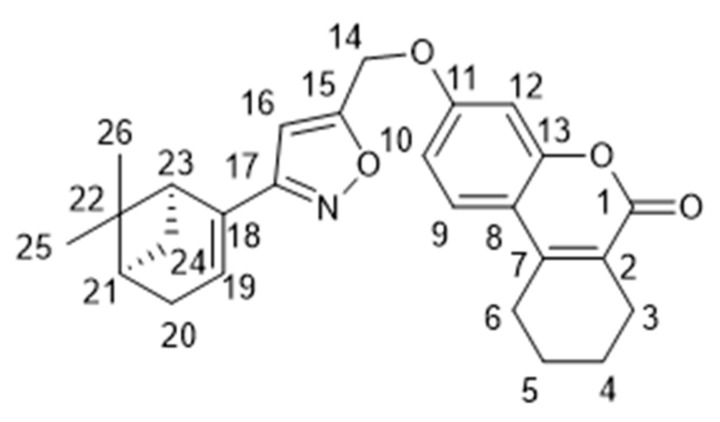
2D structure of compound **14a**, with carbon atoms enumerated for NMR assignment.

3-[3-[(1*S*,5*R*)-6,6-Dimethylbicyclo[3.1.1]hept-2-en-2-yl]isoxazol-5-yl]methoxy-7,8,9,10-tetrahydro-6*H*-benzo[c]chromen-6-one (**14a**) (Figure 7)

21% yield; white solid. m.p. 133.1 °C. ${\left[\alpha \right]}_{589}^{23.4}$ = +29.0 (c = 1.00, CHCl_3_). ^1^H NMR (400 MHz, CDCl_3_) δ 7.45 (d, *J =* 9.0 Hz, 1H, H-9), 6.87 (dd, *J =* 9.0, 2.6 Hz, 1H, H-10), 6.83 (d, *J =* 2.6 Hz, 1H, H-12), 6.42 (s, 1H, H-16), 6.14–6.20 (m, 1H, H-19), 5.13 (s, 2H, H-14), 2.95 (t, *J =* 5.7 Hz, 1H, H-23), 2.71 (t, *J =* 6.5 Hz, 2H, H-6), 2.53 (t, *J =* 6.5 Hz, 2H, H-3), 2.44–2.51 (m, 1H, H-24b), 2.35–2.50 (m, 2H, H-20), 2.13–2.17 (m, 1H, H-21), 1.71–1.87 (m, 4H, H-4, H-5), 1.33 (s, 3H, H-26), 1.22 (d, *J =* 8.7 Hz, 1H, H-24a), 0.82 (s, 3H, H-25). ^13^C NMR (101 MHz, CDCl_3_) δ 166.04 (s, C-15), 162.36 (s, C-17), 161.77 (s, C-1), 159.22 (s, C-11), 153.18 (s, C-13), 146.93 (s, C-7), 137.44 (s, C-18), 127.03 (d, C-9), 124.29 (d, C-19), 121.08 (s, C-2), 114.52 (s, C-8), 111.94 (d, C-10), 101.63 (d, C-16), 100.52 (d, C-12), 61.24 (t, C-14), 42.35 (d, C-23), 40.35 (d, C-21), 37.71 (s, C-22), 32.05 (t, C-24), 31.09 (t, C-20), 25.84 (q, C-26), 25.08 (t, C-6), 23.72 (t, C-3), 21.49 (t, C-5), 21.20 (t, C-4), 20.77 (q, C-25). HRMS *m*/*z*: [M]^+^ Calcd. for C_26_H_27_NO_4_ 417.1935; Found 417.1932.

**Figure 8:**
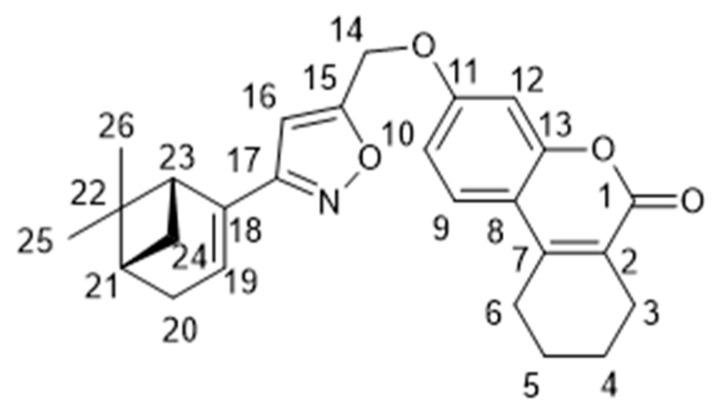
2D structure of compound **14b**, with carbon atoms enumerated for NMR assignment.

3-[3-[(1*R*,5*S*)-6,6-Dimethylbicyclo[3.1.1]hept-2-en-2-yl]isoxazol-5-yl]methoxy-7,8,9,10-tetrahydro-6*H*-benzo[c]chromen-6-one (**14b**) (Figure 8)

20% yield; white solid. m.p. 133.0 °C. ${\left[\alpha \right]}_{589}^{23.4}$ = −28.0 (c = 1.00, CHCl_3_). HRMS *m*/*z*: [M]^+^ Calcd. for C_26_H_27_NO_4_ 417.1935; Found 417.1937. Spectral data are identical to those for compound **14a**.

**Figure 9:**
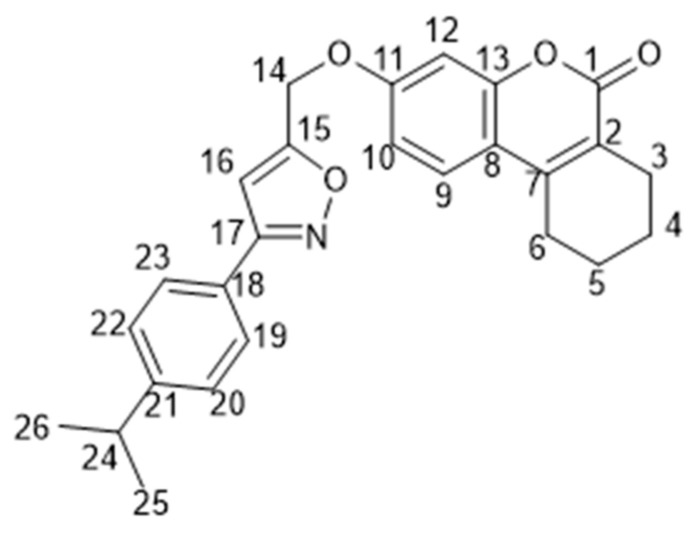
2D structure of compound **14c**, with carbon atoms enumerated for NMR assignment.

3-[3-(4-Isopropylphenyl)isoxazol-5-yl]methoxy-7,8,9,10-tetrahydro-6*H*-benzo[c]chromen-6-one (**14c**) (Figure 9)

39% yield; yellow solid. m.p. 176.2 °C. ^1^H NMR (400 MHz, CDCl_3_) δ 7.70 (dt, *J =* 8.2, 1.7 Hz, 2H, H-19,23), 7.45 (d, *J =* 8.6 Hz, 1H, H-9), 7.28 (dt, *J =* 8.2, 1.7 Hz, 2H, H-20,22), 6.89 (dd, *J =* 8.6, 2.4 Hz, 1H, H-10), 6.86 (d, *J =* 2.4 Hz, 1H, H-12), 6.63 (s, 1H, H-16), 5.21 (s, 2H, H-14), 2.92 (h, *J =* 6.9 Hz, 1H, H-24), 2.71 (tt, *J =* 6.1, 2.0 Hz, 2H, H-6), 2.52 (tt, *J =* 6.1, 2.0 Hz, 2H, H-3), 1.70–1.88 (m, 4H, H-4, H-5), 1.24 (d, *J =* 6.9 Hz, 6H, H-25,26). ^13^C NMR (101 MHz, CDCl_3_) δ 167.02 (s, C-15), 162.37 (s, C-17), 161.72 (s, C-1), 159.19 (s, C-11), 153.18 (s, C-13), 151.15 (s, C-18), 146.89 (s, C-7), 126.90 (d, 2C, C-20,22), 126.71 (d, 2C, C-19,23), 125.93 (s, C-21), 124.30 (d, C-9), 121.13 (s, C-2), 114.59 (s, C-8), 111.93 (d, C-10), 101.73 (d, C-16), 101.69 (d, C-12), 61.32 (t, C-14), 33.90 (d, C-24), 25.06 (t, C-6), 23.72 (t, C-3), 23.65 (q, 2C, C-25,26), 21.47 (t, C-5), 21.19 (t, C-4). HRMS *m*/*z*: [M]^+^ Calcd. for C_26_H_25_NO_4_ 415.1778; Found 415.1782.

**Figure 10:**
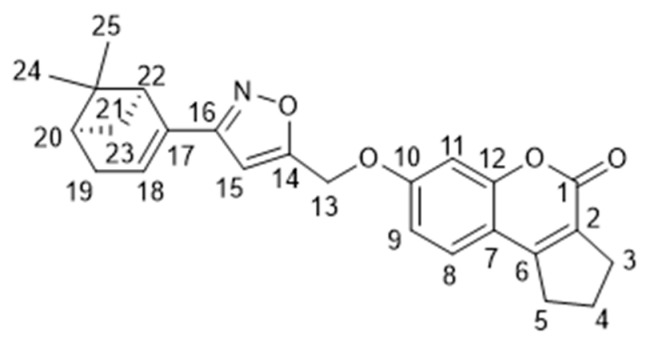
2D structure of compound **15a**, with carbon atoms enumerated for NMR assignment.

7-[3-[(1*S*,5*R*)-6,6-Dimethylbicyclo[3.1.1]hept-2-en-2-yl]isoxazol-5-yl]methoxy-2,3-dihydrocyclopenta[c]chromen-4(1*H*)-one (**15a**) (Figure 10)

25% yield; white solid. m.p. 125.1 °C. ${\left[\alpha \right]}_{589}^{23.4}$ = +37.0 (c = 1.00, CHCl_3_). ^1^H NMR (300 MHz, CDCl_3_) δ 7.34 (d, *J =* 8.3 Hz, 1H, H-8), 6.88–6.91 (m, 1H, H-11), 6.86–6.90 (m, 1H, H-9), 6.43 (s, 1H, H-15), 6.17 (tt, *J =* 3.2, 1.6 Hz, 1H, H-18), 5.14 (s, 2H, H-13), 3.02 (tt, *J =* 8.3, 1.9 Hz, 2H, H-5), 2.95 (td, *J =* 5.7, 1.6 Hz, 1H, H-22), 2.86 (tt, *J =* 7.5, 1.8 Hz, 2H, H-3), 2.49 (dt, *J =* 8.9, 5.7 Hz, 1H, H-23b), 2.39–2.47 (m, 2H, H-19), 2.11–2.21 (m, 3H, H-4, H-20), 1.33 (s, 3H, H-25), 1.19–1.26 (m, 1H, H-23a), 0.82 (s, 3H, H-24). ^13^C NMR (75 MHz, CDCl_3_) δ 165.93 (s, C-14), 162.36 (s, C-16), 160.14 (s, C-1), 159.76 (s, C-10), 155.98 (s, C-6), 155.40 (s, C-12), 137.41 (s, C-17), 127.09 (d, C-8), 125.75 (d, C-18), 125.07 (s, C-2), 113.14 (s, C-7), 112.16 (d, C-9), 101.74 (d, C-15), 100.56 (d, C-11), 61.24 (t, C-13), 42.30 (d, C-22), 40.32 (d, C-20), 37.70 (s, C-21), 32.04 (t, C-5), 31.92 (t, C-23), 31.08 (t, C-19), 30.26 (t, C-3), 25.83 (q, C-25), 22.38 (t, C-4), 20.77 (q, C-24). HRMS *m*/*z*: [M]^+^ Calcd. for C_25_H_25_NO_4_ 403.1778; Found 403.1774.

**Figure 11:**
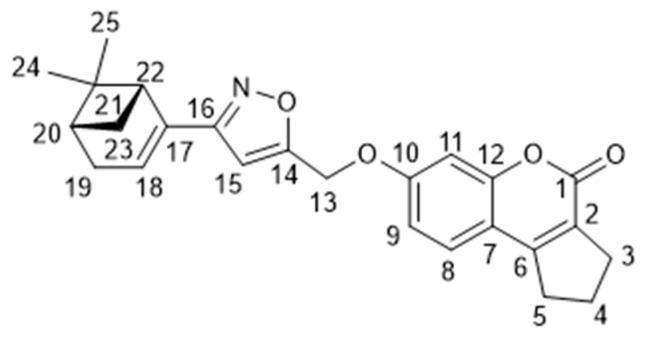
2D structure of compound **15b**, with carbon atoms enumerated for NMR assignment.

7-[3-[(1*R*,5*S*)-6,6-Dimethylbicyclo[3.1.1]hept-2-en-2-yl]isoxazol-5-yl]methoxy-2,3-dihydrocyclopenta[c]chromen-4(1*H*)-one (**15b**) (Figure 11)

23% yield; white solid. m.p. 123.9 °C. ${\left[\alpha \right]}_{589}^{23.4}$ = −26.6 (c = 1.00, CHCl_3_). HRMS *m*/*z*: [M]^+^ Calcd. for C_25_H_25_NO_4_ 403.1778; Found 403.1776. Spectral data are identical to those for compound **15a**.

**Figure 12:**
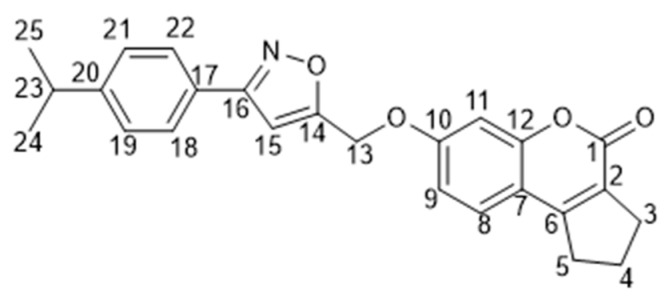
2D structure of compound **15c**, with carbon atoms enumerated for NMR assignment.

7-[3-(4-Isopropylphenyl)isoxazol-5-yl]methoxy-2,3-dihydrocyclopenta[c]chromen-4(1*H*)-one (**15c**) (Figure 12)

32% yield; white solid. m.p. 129.0 °C. ^1^H NMR (500 MHz, CDCl_3_) δ 7.70 (d, *J =* 7.9 Hz, 2H, H-18,22), 7.34 (d, *J =* 8.5 Hz, 1H, H-8), 7.29 (d, *J =* 7.9 Hz, 2H, H-19,21), 6.88–6.94 (m, 2H, H-9,11), 6.65 (s, 1H, H-15), 5.22 (s, 2H, H-13), 3.01 (t, *J =* 7.6 Hz, 2H, H-5), 2.92 (h, *J =* 6.9 Hz, 1H, H-23), 2.86 (t, *J =* 7.5 Hz, 2H, H-3), 2.17 (p, *J =* 7.6 Hz, 2H, H-4), 1.24 (d, *J =* 6.9 Hz, 6H, H-24,25). ^13^C NMR (126 MHz, CDCl_3_) δ 166.86 (s, C-14), 162.34 (s, C-16), 160.12 (s, C-1), 159.66 (s, C-10), 155.99 (s, C-6), 155.34 (s, C-12), 151.14 (s, C-17), 126.90 (d, 2C, C-19,21), 126.67 (d, 2C, C-18,22), 125.83 (s, C-20), 125.76 (d, C-8), 125.06 (s, C-2), 113.15 (s, C-7), 112.12 (d, C-9), 101.74 (d, 2C, C-11,15), 61.26 (t, C-13), 33.88 (d, C-23), 31.88 (t, C-5), 30.23 (t, C-3), 23.66 (q, 2C, C-24,25), 22.33 (t, C-4). HRMS *m*/*z*: [M]^+^ Calcd. for C_25_H_23_NO_4_ 401.1622; Found 401.1620.

**Figure 13:**
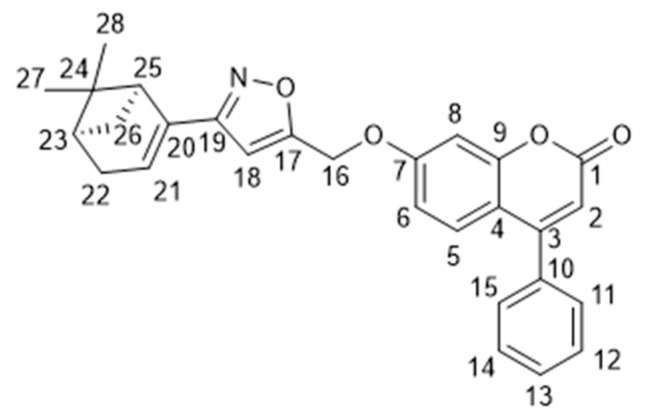
2D structure of compound **16a**, with carbon atoms enumerated for NMR assignment.

7-[3-[(1*S*,5*R*)-6,6-Dimethylbicyclo[3.1.1]hept-2-en-2-yl]isoxazol-5-yl]methoxy-4-phenyl-2*H*-chromen-2-one (**16a**) (Figure 13)

25% yield; yellow amorphous solid. ${\left[\alpha \right]}_{589}^{23.4}$ = +21.8 (c = 1.00, CHCl_3_). ^1^H NMR (400 MHz, CDCl3) δ 7.48–7.56 (m, 3H, H-12,13,14), 7.39–7.46 (m, 3H, H-5,11,15), 6.97 (d, *J =* 2.6 Hz, 1H, H-8), 6.86 (dd, *J =* 8.9, 2.6 Hz, 1H, H-6), 6.47 (s, 1H, H-18), 6.25 (s, 1H, H-2), 6.21 (tt, *J =* 3.3, 1.5 Hz, 1H, H-21), 5.20 (s, 2H, H-16), 2.98 (td, *J =* 5.6, 1.6 Hz, 1H, H-25), 2.52 (td, *J =* 5.7, 3.4 Hz, 1H, H-26b), 2.39–2.49 (m, 2H, H-22), 2.15–2.22 (m, 1H, H-23), 1.36 (s, 3H, H-28), 1.24–1.27 (m, 1H, H-26a), 0.86 (s, 3H, H-27). ^13^C NMR (126 MHz, CDCl_3_) δ 165.69 (s, C-17), 162.33 (s, C-19), 160.83 (s, C-7), 160.50 (s, C-1), 155.58 (s, C-9), 155.47 (s, C-3), 137.33 (s, C-20), 135.13 (s, C-10), 129.52 (d, C-13), 128.70 (d, 2C, C-12,14), 128.18 (d, C-15), 128.14 (d, C-11), 127.12 (d, C-5), 125.46 (d, C-21), 113.19 (s, C-4), 112.26 (d, C-6), 112.24 (d, C-2), 101.99 (d, C-18), 100.60 (d, C-8), 61.20 (t, C-16), 42.24 (d, C-25), 40.26 (d, C-23), 37.66 (s, C-24), 32.00 (t, C-26), 31.03 (t, C-22), 25.78 (q, C-28), 20.73 (q, C-27). HRMS *m*/*z*: [M]+ Calcd. for C_28_H_25_NO_4_ 439.1778; Found 439.1779.

**Figure 14:**
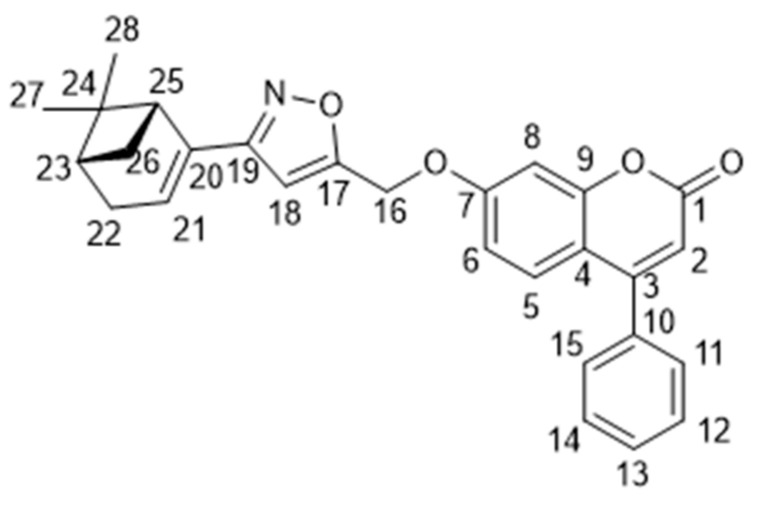
2D structure of compound **16b**, with carbon atoms enumerated for NMR assignment.

7-[3-[(1*R*,5*S*)-6,6-Dimethylbicyclo[3.1.1]hept-2-en-2-yl]isoxazol-5-yl]methoxy-4-phenyl-2*H*-chromen-2-one (**16b**) (Figure 14)

25% yield; yellow amorphous solid. ${\left[\alpha \right]}_{589}^{23.4}$ = −22.0 (c = 1.00, CHCl_3_). HRMS *m*/*z*: [M]^+^ Calcd. for C_28_H_25_NO_4_ 439.1778; Found 439.1772. Spectral data are identical to those for compound **16a**.

**Figure 15:**
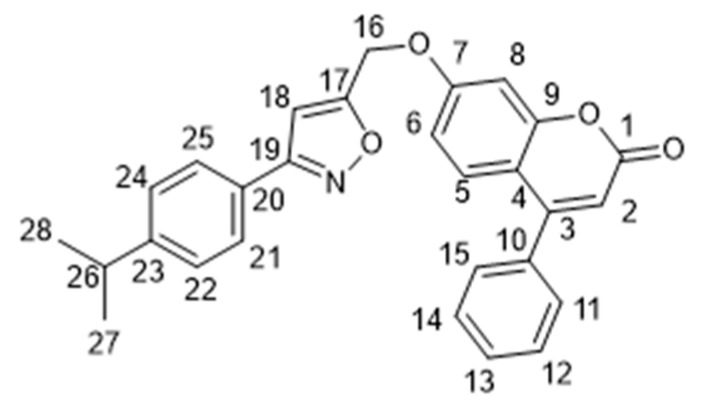
2D structure of compound **16c**, with carbon atoms enumerated for NMR assignment.

7-[3-(4-Isopropylphenyl)isoxazol-5-yl]methoxy-4-phenyl-2*H*-chromen-2-one (**16c**) (Figure 15)

30% isolated yield; yellow solid. m.p. 115.4 °C. ^1^H NMR (500 MHz, CDCl_3_) δ 7.71 (d, *J =* 7.9 Hz, 2H, H-21,25), 7.47–7.53 (m, 3H, H-12,13,14), 7.39–7.44 (m, 3H, H-5,11,15), 7.30 (d, *J =* 7.9 Hz, 2H, H-22,24), 6.98 (d, *J =* 2.5 Hz, 1H, H-8), 6.86 (dd, *J =* 9.0, 2.5 Hz, 1H, H-6), 6.66 (s, 1H, H-18), 6.23 (s, 1H, H-2), 5.25 (s, 2H, H-16), 2.93 (h, *J =* 6.7 Hz, 1H, H-26), 1.25 (d, *J =* 6.7 Hz, 6H, H-27,28). ^13^C NMR (126 MHz, CDCl_3_) δ 166.69 (s, C-17), 162.39 (s, C-19), 160.83 (s, C-7), 160.47 (s, C-1), 155.61 (s, C-9), 155.48 (s, C-3), 151.20 (s, C-23), 135.15 (s, C-10), 129.56 (d, C-13), 128.74 (d, 2C, C-12,14), 128.21 (d, 2C, C-11,15), 127.99 (d, C-5), 126.93 (d, 2C, C-22,24), 126.71 (d, 2C, C-21,25), 125.82 (s, C-20), 113.29 (d, C-2), 112.35 (d, C-6), 112.25 (s, C-4), 102.11 (d, C-18), 101.83 (d, C-8), 61.30 (t, C-16), 33.91 (d, C-26), 23.68 (q, 2C, C-27,28). HRMS *m*/*z*: [M]+ Calcd. for C_28_H_23_NO_4_ 437.1622; Found 437.1625.

**Figure 16:**
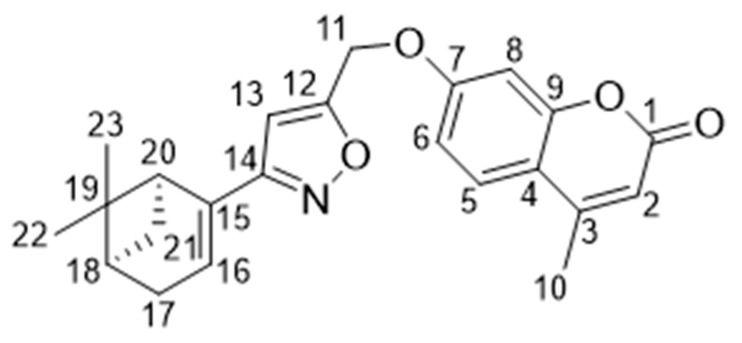
2D structure of compound **17a**, with carbon atoms enumerated for NMR assignment.

7-[3-[(1*S*,5*R*)-6,6-Dimethylbicyclo[3.1.1]hept-2-en-2-yl]isoxazol-5-yl]methoxy-4-methyl-2*H*-chromen-2-one (**17a**) (Figure 16)

26% yield; white solid. m.p. 109.7 °C. ${\left[\alpha \right]}_{589}^{23.4}$ = +28.0 (c = 1.00, CHCl_3_). ^1^H NMR (400 MHz, CDCl_3_) δ 7.50 (d, *J =* 8.8 Hz, 1H, H-5), 6.90 (dd, *J =* 8.8, 2.6 Hz, 1H, H-6), 6.87 (d, *J =* 2.6 Hz, 1H, H-8), 6.43 (s, 1H, H-13), 6.18 (s, 1H, H-2), 6.14 (s, 1H, H-16), 5.16 (s, 2H, H-11), 2.95 (td, *J =* 5.7, 1.5 Hz, 1H, H-20), 2.49 (dt, *J =* 9.0, 5.7 Hz, 1H, H-21b), 2.37–2.49 (m, 2H, H-17), 2.37 (s, 3H, H-10), 2.16 (tt, *J =* 3.4, 2.8 Hz, 1H, H-18), 1.34 (s, 3H, H-23), 1.23 (d, *J =* 9.0 Hz, 1H, H-21a), 0.83 (s, 3H, H-22). ^13^C NMR (101 MHz, CDCl_3_) δ 165.85 (s, C-12), 162.40 (s, C-14), 160.82 (s, C-7), 160.48 (s, C-1), 154.99 (s, C-9), 152.15 (s, C-3), 137.48 (s, C-15), 127.05 (d, C-5), 125.71 (d, C-16), 114.35 (s, C-4), 112.47 (d, C-6), 112.25 (d, C-2), 101.94 (d, C-13), 100.60 (d, C-8), 61.32 (t, C-11), 42.45 (d, C-20), 40.42 (d, C-18), 37.73 (s, C-19), 32.07 (t, C-21), 31.11 (t, C-17), 25.87 (q, C-23), 20.79 (q, C-22), 18.51 (q, C-10). HRMS *m*/*z*: [M]^+^ Calcd. for C_23_H_23_NO_4_ 377.1622; Found 377.1623.

**Figure 17:**
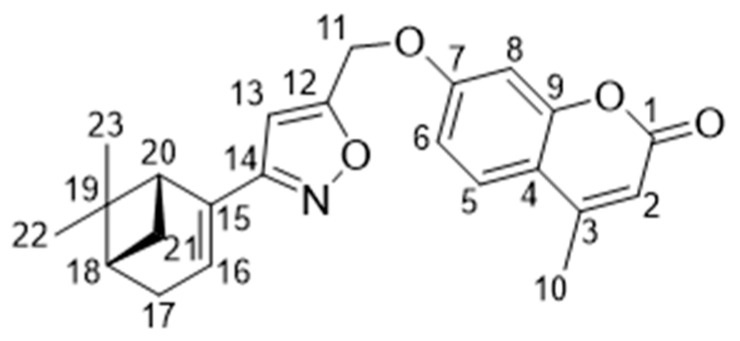
2D structure of compound **17b**, with carbon atoms enumerated for NMR assignment.

7-[3-[(1*R*,5*S*)-6,6-Dimethylbicyclo[3.1.1]hept-2-en-2-yl]isoxazol-5-yl]methoxy-4-methyl-2*H*-chromen-2-one (**17b**) (Figure 17)

47% yield; white solid. m.p. 110.1 °C. ${\left[\alpha \right]}_{589}^{23.4}$ = −22.2 (c = 1.00, CHCl_3_). HRMS *m*/*z*: [M]^+^ Calcd. for C_23_H_23_NO_4_ 377.1622; Found 377.1620. Spectral data are identical to those for compound **17a**.

**Figure 18:**
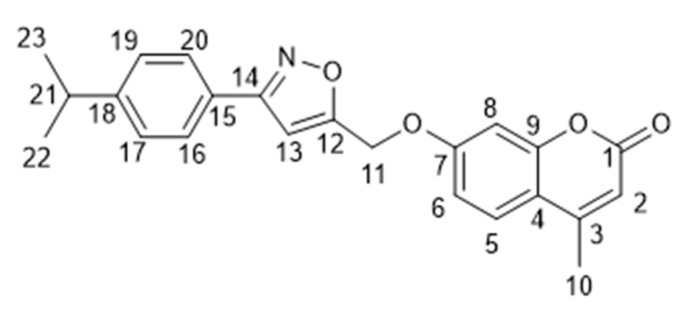
2D structure of compound **17c**, with carbon atoms enumerated for NMR assignment.

7-[3-(4-Isopropylphenyl)isoxazol-5-yl]methoxy-4-methyl-2*H*-chromen-2-one (**17c**) (Figure 18)

30% yield; white solid. m.p. 154.8 °C. ^1^H NMR (400 MHz, CDCl_3_) δ 7.71 (d, *J =* 8.1 Hz, 2H, H-16,20), 7.52 (d, *J =* 8.7 Hz, 1H, H-5), 7.30 (d, *J =* 8.0 Hz, 2H, H-17,19), 6.88–6.96 (m, 2H, H-6, H-8), 6.65 (s, 1H, H-13), 6.15 (s, 1H, H-2), 5.24 (s, 2H, H-11), 2.92 (h, *J =* 6.9 Hz, 1H, H-21), 2.39 (s, 3H, H-10), 1.25 (d, *J =* 6.9 Hz, 6H, H-22,23). ^13^C NMR (101 MHz, CDCl_3_) δ 166.76 (s, C-12), 162.42 (s, C-14), 160.91 (s, C-7), 160.37 (s, C-1), 154.95 (s, C-9), 152.25 (s, C-3), 151.23 (s, C-15), 126.96 (d, 2C, C-17,19), 126.73 (d, 2C, C-16,20), 125.86 (s, C-18), 125.77 (d, C-5), 114.39 (s, C-4), 112.50 (d, C-6), 112.26 (d, C-2), 101.92 (d, C-13), 101.81 (d, C-8), 61.33 (t, C-11), 33.94 (d, C-21), 23.70 (q, 2C, C-22,23), 18.60 (q, C-10). HRMS *m*/*z*: [M]+ Calcd. for C_23_H_21_NO_4_ 375.1465; Found 375.1463.

#### 3.1.3. Synthesis of β-Ketoester **25**

Acetophenone **24** (33.29 mmol) was added dropwise to a mixture of sodium hydride (99.87 mmol), previously washed three times with toluene, and diethyl carbonate (133.16 mmol) in toluene (50 mL) under an argon atmosphere. The mixture was refluxed for 4 h and then poured into ice water. Glacial acetic acid (5 mL) was added to the mixture, and the mixture was extracted three times with ethyl acetate. The collected organic phase was washed with a saturated solution of sodium bicarbonate, saturated brine, and water; then dried over anhydrous Na_2_SO_4_ and distilled on a rotary evaporator; and then purified by column chromatography with methylene chloride. The yield of the target compound was 74%. The NMR spectra correspond to data from the literature [68].

#### 3.1.4. Synthesis of 7-Hydroxycoumarin **18e**

A total of 5.24 g (24.75 mmol) of resorcinol was added to 5.5 g (24.75 mmol) of ethyl 3-(4-methoxyphenyl)-3-oxopropanoate **25** in 12.5 mL of ethanol with stirring and cooling to 0–5 °C. After resorcinol dissolved, 5 mL of H_2_SO_4_ (conc.) was added. The reaction mixture was stirred overnight. After the solution thickened, 100 mL of cold water was added, and the mixture was filtered. The mixture was recrystallized from ethanol. The yield of the target compound was 63%. The spectral data correspond to the literature [69].

#### 3.1.5. Synthesis of 7-Hydroxycoumarin Propargyl Ether **13e**

A round-bottomed flask equipped with a reflux condenser was charged with 2.5 mmol of hydroxycoumarin 18e, 5 mmol of propargyl bromide, and 5 mmol of K_2_CO_3_. The mixture was dissolved in 20 mL of ethanol and stirred under reflux. After 5 h, heating was stopped, the solvent was evaporated, ice water was added to the flask, the mixture was filtered, and the undissolved precipitate was collected. The yield of the target compound was 80%.

**Figure 19:**
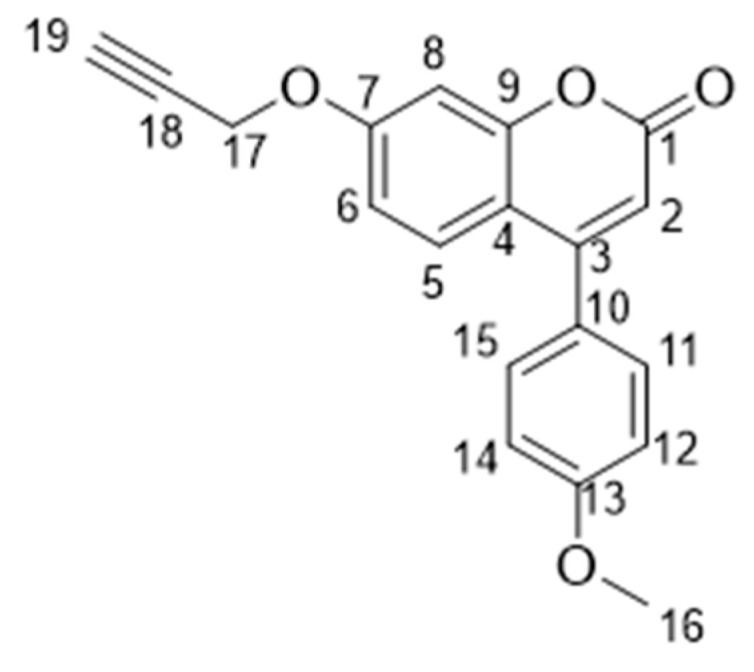
2D structure of compound **13e**, with carbon atoms enumerated for NMR assignment.

4-(4-Methoxyphenyl)-7-(prop-2-yn-1-yloxy)-2*H*-chromen-2-one (**13e**) (Figure 19)

Light brown solid. m.p. 186.0 °C. ^1^H NMR (400 MHz, CDCl_3_) δ 7.46 (d, *J =* 8.9 Hz, 1H, H-5), 7.37 (d, *J =* 8.5 Hz, 2H, H-11,15), 7.01 (d, *J =* 8.5 Hz, 2H, H-12,14), 6.98 (d, *J =* 2.5 Hz, 1H, H-8), 6.85 (dd, *J =* 8.9, 2.5 Hz, 1H, H-6), 6.19 (s, 1H, H-2), 4.75 (d, *J =* 2.5 Hz, 2H, H-17), 3.86 (s, 3H, H-16), 2.56 (t, *J =* 2.5 Hz, 1H, H-19). ^13^C NMR (101 MHz, CDCl_3_) δ 161.14 (s, C-7), 160.65 (s, C-1), 160.23 (s, C-9), 155.63 (s, C-3), 155.25 (s, C-10), 129.75 (d, 2C, C-11,15), 127.98 (d, 2C, C-12,14), 127.53 (s, C-13), 114.16 (d, C-5), 113.20 (s, C-4), 112.55 (d, C-6), 111.67 (d, C-2), 102.20 (d, C-8), 77.24 (d, C-19), 76.42 (s, C-18), 56.04 (t, C-17), 55.32 (q, C-16). HRMS *m*/*z*: [M]^+^ Calcd. for C_19_H_14_O_4_ 306.0887; Found 306.0886.

#### 3.1.6. General Procedure for the Synthesis of Triazole-Containing Monoterpene–Coumarin Conjugates

Copper(II) sulfate pentahydrate (0.18 mmol) and sodium ascorbate (0.18 mmol) were sequentially added to a solution of coumarin ester (0.72 mmol) in a mixture of methylene chloride, tert-butanol, and water (4:2:1, 15 mL). The mixture was stirred for 15 min until a bright-yellow color appeared. The corresponding azide (1.08 mmol) was then added, and the mixture was heated to 45 °C, with stirring continued until the starting ester was completely consumed (a process monitored by decolorization of the solution and TLC). After completion of the reaction, the mixture was extracted with ethyl ether, and the organic phase was washed with water, dried over sodium sulfate, filtered, and evaporated. The product was purified by column chromatography on SiO_2_ using a hexane–ethyl ether gradient with a gradual increase in EtOAc content from 0% to 50%.

**Figure 20:**
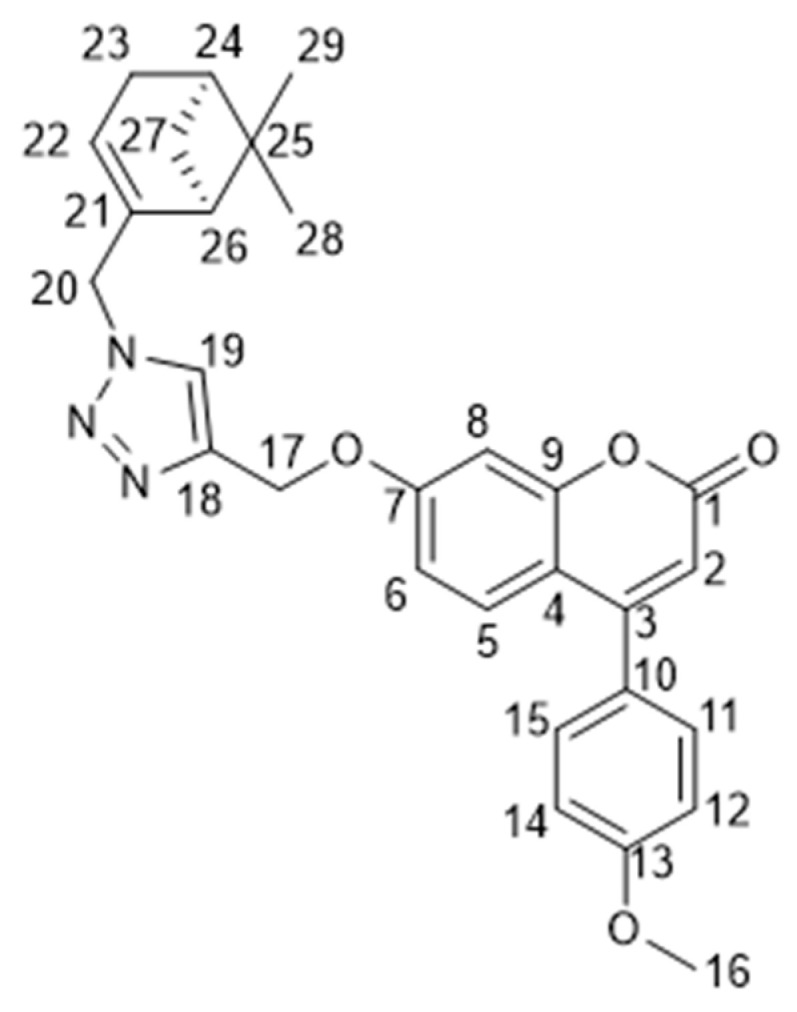
2D structure of compound **26a**, with carbon atoms enumerated for NMR assignment.

7-[1-{[(1*S*,5*R*)-6,6-Dimethylbicyclo[3.1.1]hept-2-en-2-yl]methyl}-1*H*-1,2,3-triazol-4-yl]methoxy-4-(4-methoxyphenyl)-2*H*-chromen-2-one (**26a**) (Figure 20)

57% yield; pale-yellow amorphous solid. ${\left[\alpha \right]}_{589}^{23.4}$ = +9.00 (c = 1.00, CHCl_3_). ^1^H NMR (400 MHz, CDCl_3_) δ 7.58 (s, 1H, H-19), 7.42 (d, *J =* 8.9 Hz, 1H, H-5), 7.34 (dt, *J =* 8.8, 3.0 Hz, 2H, H-11,15), 6.98 (dt, *J =* 9.0, 3.0 Hz, 2H, H-12,14), 6.93 (d, *J =* 2.5 Hz, 1H, H-8), 6.84 (dd, *J =* 8.9, 2.5 Hz, 1H, H-6), 6.15 (s, 1H, H-2), 5.58 (hept, *J =* 1.5 Hz, 1H, H-22), 5.24 (s, 2H, H-17), 4.84 (q, *J =* 1.5 Hz, 2H, H-20), 3.85 (s, 3H, H-16), 2.32 (dt, *J =* 8.8, 5.6 Hz, 1H, H-27b), 2.24 (qh, *J =* 8.8, 1.5 Hz, 2H, H-23), 2.06 (hept, *J =* 2.9, 1.6 Hz, 1H, H-24), 1.97 (td, *J =* 5.6, 1.5 Hz, 1H, H-26), 1.15 (s, 3H, H-29), 1.07 (d, *J =* 8.8 Hz, 1H, H-27a), 0.66 (s, 3H, H-28). ^13^C NMR (101 MHz, CDCl_3_) δ 161.07 (s, C-7), 160.98 (s, C-1), 160.62 (s, C-9), 155.66 (s, C-3), 155.21 (s, C-13), 142.94 (s, C-18), 141.72 (s, C-21), 129.68 (d, 2C, C-11,15), 127.97 (d, 2C, C-12,14), 127.52 (s, C-10), 123.10 (d, C-22), 122.59 (d, C-19), 114.13 (d, C-5), 112.90 (s, C-4), 112.24 (d, C-6), 111.42 (d, C-2), 102.26 (d, C-8), 62.21 (t, C-17), 55.27 (q, C-16), 55.23 (t, C-20), 43.20 (d, C-26), 40.19 (d, C-24), 37.78 (s, C-25), 31.44 (t, C-27), 31.07 (t, C-23), 25.65 (q, C-29), 20.70 (q, C-28). HRMS *m*/*z*: [M]^+^ Calcd. for C_29_H_29_N_3_O_4_ 483.2153; Found 483.2147.

**Figure 21:**
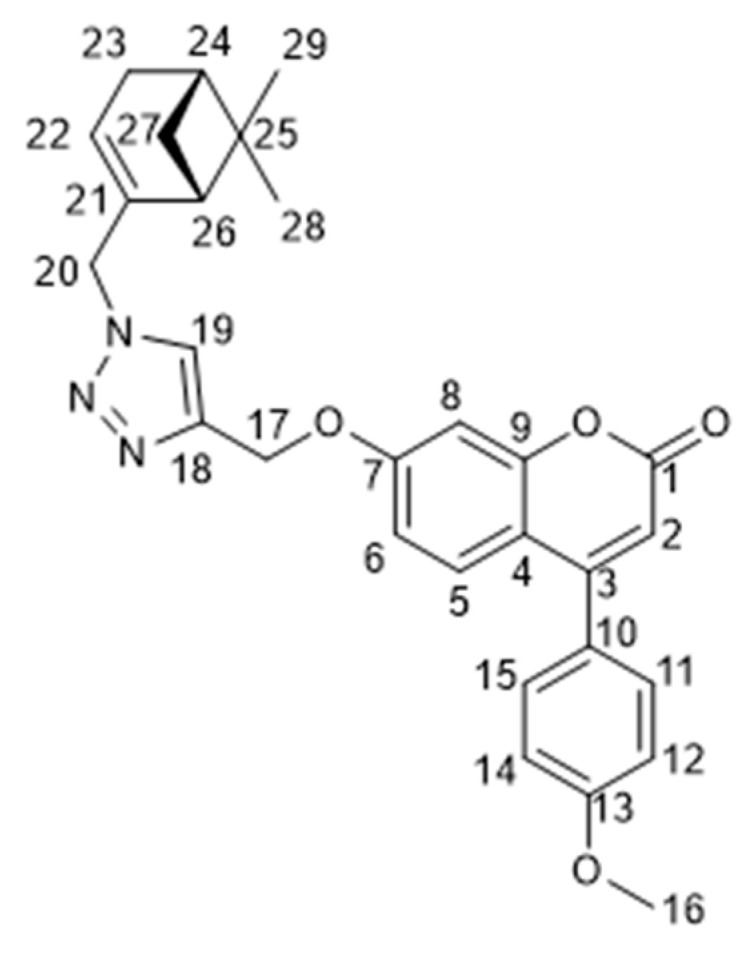
2D structure of compound **26b**, with carbon atoms enumerated for NMR assignment.

7-[1-{[(1*R*,5*S*)-6,6-Dimethylbicyclo[3.1.1]hept-2-en-2-yl]methyl}-1*H*-1,2,3-triazol-4-yl]methoxy-4-(4-methoxyphenyl)-2*H*-chromen-2-one (**26b**) (Figure 21)

33% yield; pale-yellow amorphous solid. ${\left[\alpha \right]}_{589}^{23.4}$ = −9.00 (c = 1.00, CHCl_3_). HRMS *m*/*z*: [M]^+^ Calcd. for C_29_H_29_N_3_O_4_ 483.2153; Found 483.2147. Spectral data are identical to those for compound **26a**.

**Figure 22:**
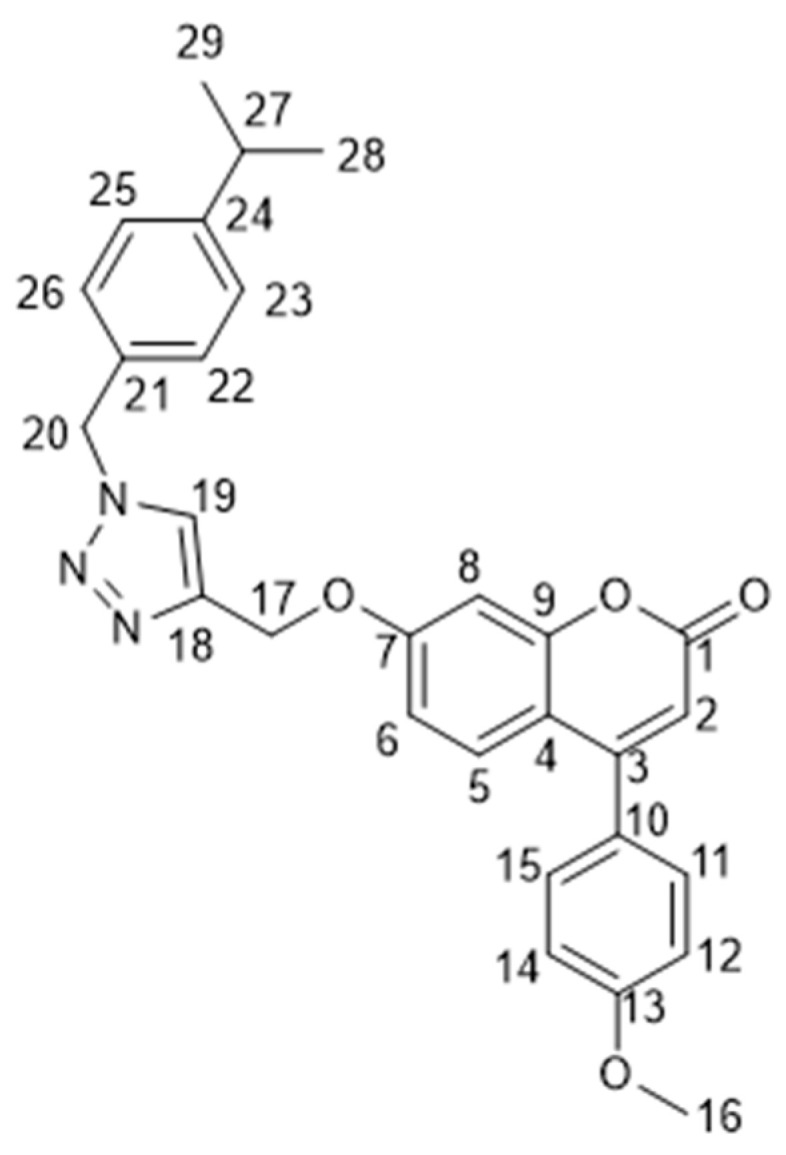
2D structure of compound **26c**, with carbon atoms enumerated for NMR assignment.

7-[1-(4-Isopropylbenzyl)-1*H*-1,2,3-triazol-4-yl]methoxy-4-(4-methoxyphenyl)-2*H*-chromen-2-one (**26c**) (Figure 22)

56% yield; solid. m.p. 49.6 °C. ^1^H NMR (400 MHz, CDCl_3_) δ 7.55 (s, 1H, H-19), 7.42 (d, *J =* 8.8 Hz, 1H, H-5), 7.33–7.38 (m, 2H, H-11,15), 7.18–7.23 (m, 4H, H-22,23,25,26), 6.97–7.02 (m, 2H, H-12,14), 6.94 (d, *J =* 2.6 Hz, 1H, H-8), 6.84 (dd, *J =* 8.8, 2.6 Hz, 1H, H-6), 6.16 (s, 1H, H-2), 5.48 (s, 2H, H-20), 5.20 (s, 2H, H-17), 3.86 (s, 3H, H-16), 2.88 (p, *J =* 6.9 Hz, 1H, H-27), 1.21 (d, *J =* 6.9 Hz, 6H, H-28,29). ^13^C NMR (101 MHz, CDCl_3_) δ 161.18 (s, C-7), 161.11 (s, C-1), 160.72 (s, C-9), 155.76 (s, C-3), 155.31 (s, C-10), 149.73 (s, C-24), 143.17 (s, C-18), 131.54 (s, C-21), 129.78 (d, 2C, C-11,15), 128.19 (d, 2C, C-23,25), 128.07 (d, 2C, C-12,14), 127.63 (s, C-13), 127.17 (d, 2C, C-22,26), 122.79 (d, C-19), 114.22 (d, C-5), 113.04 (s, C-4), 112.29 (d, C-6), 111.55 (d, C-2), 102.25 (d, C-8), 62.26 (t, C-17), 55.35 (q, C-16), 54.05 (t, C-20), 33.77 (d, C-27), 23.79 (q, 2C, C-28,29). HRMS *m*/*z*: [M]^+^ Calcd. for C_29_H_27_N_3_O_4_ 481.1996; Found 481.2001.

##### 7-[1-(3,7-Dimethylocta-2,6-dien-1-yl)-1*H*-1,2,3-triazol-4-yl]methoxy-4-(4-methoxyphenyl)-2*H*-chromen-2-one (**26d**)

51% overall yield (purified by column chromatography, *Z*:*E* ratio = 1:2).

**Figure 23:**
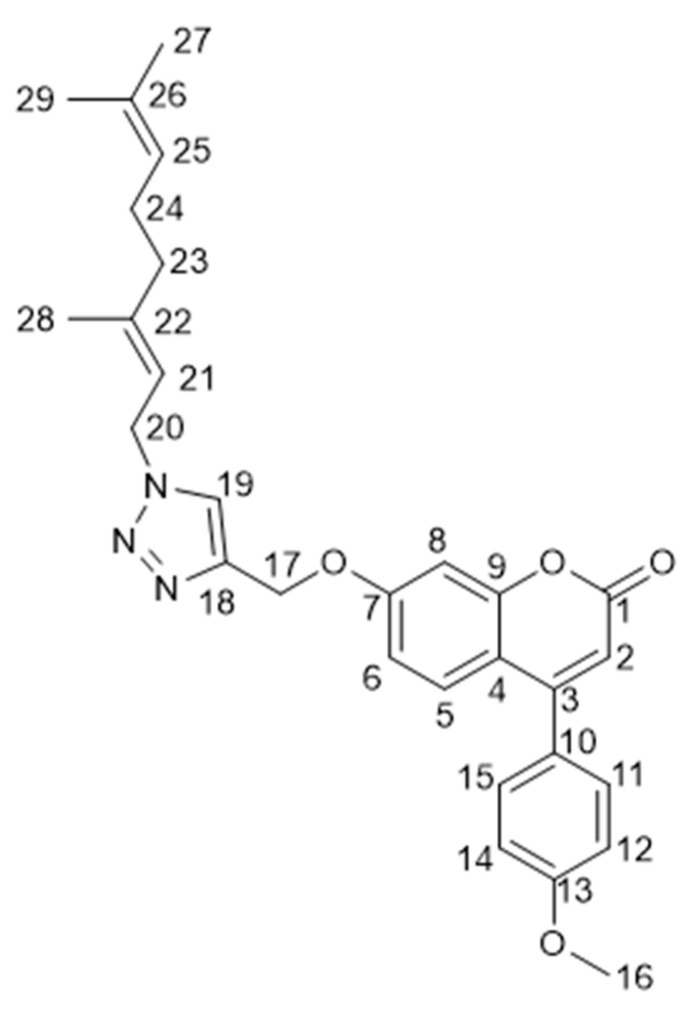
2D structure of compound **26d**(*E*), with carbon atoms enumerated for NMR assignment.

**26d**(*E*): Pale-yellow amorphous solid (Figure 23). ^1^H NMR (400 MHz, CDCl_3_) δ 7.60 (s, 1H, H-19), 7.44 (d, *J =* 8.9 Hz, 1H, H-5), 7.36 (d, *J =* 8.4 Hz, 2H, H-11,15), 7.00 (d, *J =* 8.4 Hz, 2H, H-12,14), 6.97 (s, 1H, H-8), 6.87 (d, *J =* 8.9 Hz, 1H, H-6), 6.17 (s, 1H, H-2), 5.42 (t, *J =* 7.4 Hz, 1H, H-21), 5.23 (s, 2H, H-17), 5.05 (t, *J =* 7.3 Hz, 1H, H-25), 4.94 (d, *J =* 7.4 Hz, 2H, H-20), 3.86 (s, 3H, H-16), 2.18 (t, *J =* 7.3 Hz, 2H, H-23), 2.11 (q, *J =* 7.3 Hz, 2H, H-24), 1.79 (s, 3H, H-28), 1.65 (s, 3H, H-29), 1.58 (s, 3H, H-27). ^13^C NMR (101 MHz, CDCl_3_) δ 161.16 (s, C-7), 161.09 (s, C-1), 160.64 (s, C-9), 155.71 (s, C-3), 155.26 (s, C-13), 143.38 (s, C-22), 142.75 (s, C-18), 132.64 (s, C-26), 129.74 (d, 2C, C-11,15), 128.03 (d, 2C, C-12,14), 127.57 (s, C-10), 122.96 (d, C-25), 122.18 (d, C-19), 117.40 (d, C-21), 114.15 (d, C-5), 112.96 (s, C-4), 112.20 (d, C-6), 111.48 (d, C-2), 102.20 (d, C-8), 62.25 (t, C-17), 55.30 (q, C-16), 47.75 (t, C-20), 31.95 (t, C-23), 26.09 (t, C-24), 25.60 (q, C-29), 23.28 (q, C-28), 17.58 (q, C-27). HRMS *m*/*z*: [M]^+^ Calcd. for C_29_H_31_N_3_O_4_ 485.2307; Found 485.2309.

**Figure 24:**
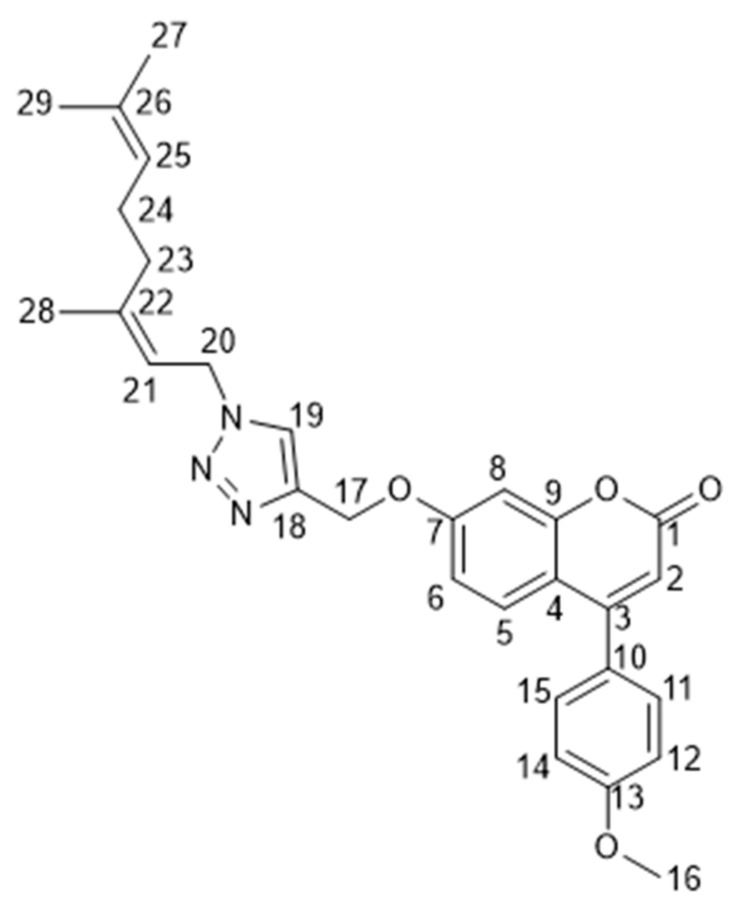
2D structure of compound **26d**(*Z*), with carbon atoms enumerated for NMR assignment.

**26d**(*Z*): Pale-yellow amorphous solid (Figure 24). ^1^H NMR (400 MHz, CDCl_3_) δ 7.59 (s, 1H, H-19), 7.44 (d, *J =* 9.0 Hz, 1H, H-5), 7.36 (d, *J =* 8.8 Hz, 2H, H-11,15), 7.00 (d, *J =* 8.8 Hz, 2H, H-12,14), 6.96 (s, 1H, H-8), 6.87 (d, *J =* 9.0 Hz, 1H, H-6), 6.17 (s, 1H, H-2), 5.41 (t, *J =* 7.4 Hz, 1H, H-21), 5.22 (s, 2H, H-17), 5.00–5.05 (m, 1H, H-25), 4.96 (d, *J =* 7.3 Hz, 2H, H-20), 3.86 (s, 3H, H-16), 2.09 (s, 4H, H-23,24), 1.76 (s, 3H, H-27), 1.64 (s, 3H, H-28), 1.56 (s, 3H, H-29). ^13^C NMR (101 MHz, CDCl_3_) δ 161.13 (s, C-7), 161.07 (s, C-1), 160.62 (s, C-9), 155.70 (s, C-3), 155.24 (s, C-13), 143.56 (s, C-22), 142.70 (s, C-18), 132.06 (s, C-26), 129.72 (d, 2C, C-11,15), 128.01 (d, 2C, C-12,14), 127.55 (s, C-10), 123.25 (d, C-25), 122.17 (d, C-19), 116.55 (d, C-21), 114.13 (d, C-5), 112.94 (s, C-4), 112.19 (d, C-6), 111.46 (d, C-2), 102.17 (d, C-8), 62.23 (t, C-17), 55.29 (q, C-16), 47.88 (t, C-20), 39.22 (t, C-23), 25.89 (t, C-24), 25.55 (q, C-29), 17.58 (q, C-27), 16.33 (q, C-28). HRMS *m*/*z*: [M]^+^ Calcd. for C_29_H_31_N_3_O_4_ 485.2307; Found 485.2309.

**Figure 25:**
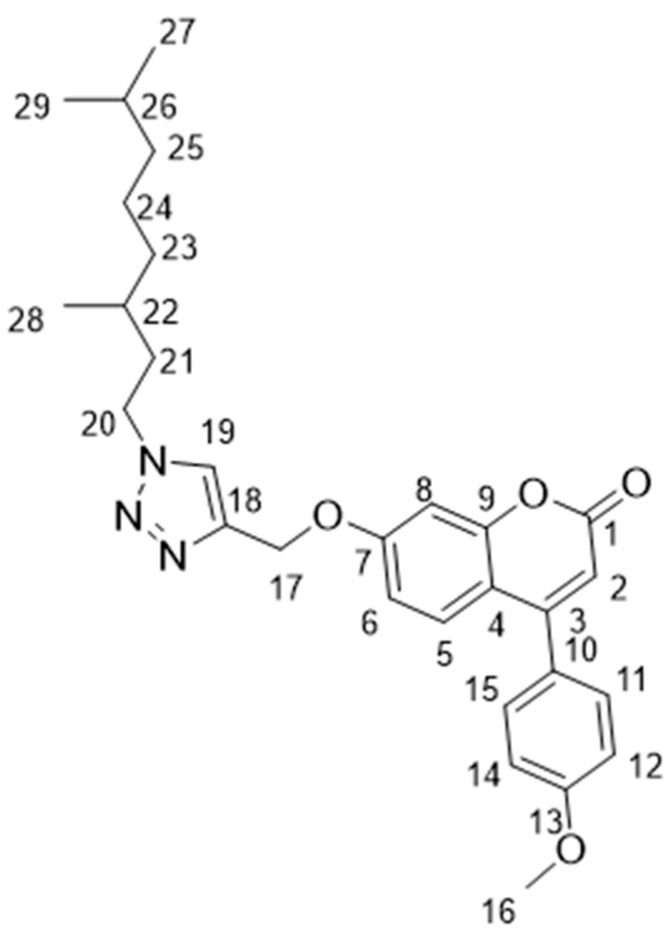
2D structure of compound **26e**, with carbon atoms enumerated for NMR assignment.

7-[1-(3,7-Dimethyloctyl)-1*H*-1,2,3-triazol-4-yl]methoxy-4-(4-methoxyphenyl)-2*H*-chromen-2-one (**26e**) (Figure 25)

73% yield; orange liquid. ^1^H NMR (400 MHz, CDCl3) δ 7.67 (s, 1H, H-19), 7.47 (d, *J =* 8.9 Hz, 1H, H-5), 7.36–7.42 (m, 2H, H-11,15), 7.00–7.06 (m, 2H, H-12,14), 6.99 (d, *J =* 2.5 Hz, 1H, H-8), 6.90 (dd, *J =* 9.0, 2.5 Hz, 1H, H-6), 6.20 (s, 1H, H-2), 5.27 (s, 2H, H-17), 4.33–4.49 (m, 2H, H-20), 3.89 (s, 3H, H-16), 1.87–2.04 (m, 1H, H-21b), 1.64–1.83 (m, 1H, H-21a), 1.40–1.57 (m, 2H, H-22,26), 1.11–1.34 (m, 6H, H-23,24,25), 0.96 (d, *J =* 6.7 Hz, 3H, H-28), 0.86 (d, *J =* 6.7 Hz, 6H, H-27,29). ^13^C NMR (101 MHz, CDCl_3_) δ 161.14 (s, C-7), 161.05 (s, C-1), 160.63 (s, C-9), 155.70 (s, C-3), 155.25 (s, C-13), 142.75 (s, C-18), 129.72 (d, 2C, C-11,15), 128.02 (d, 2C, C-12,14), 127.54 (s, C-10), 122.52 (d, C-19), 114.14 (d, C-5), 112.95 (s, C-4), 112.22 (d, C-6), 111.46 (d, C-2), 102.17 (d, C-8), 62.21 (t, C-17), 55.29 (q, C-16), 48.61 (t, C-20), 38.93 (t, C-21), 37.17 (t, C-23), 36.69 (t, C-25), 30.19 (d, C-22), 27.75 (d, C-26), 24.35 (t, C-24), 22.50 (q, C-29), 22.41 (q, C-27), 19.12 (q, C-28). HRMS *m*/*z*: [M]^+^ Calcd. for C_29_H_35_N_3_O_4_ 489.2622; Found 489.2625.

### 3.2. Molecular Modeling

Molecular modelling, docking and binding energy calculations were performed using Maestro 2025-1 [70]. All ligands were modelled and optimized using LigPrep (2025-1, Schrödinger LLC, New York, NY) and MacroModel (2025-1, Schrödinger LLC, New York, NY) [71] according to the OPLS4 (2025-1, Schrödinger LLC, New York, NY) [72] forcefield parameters. Molecular descriptors were calculated and oral bioavailability radars were plotted on SwissADME (www.swissadme.ch) [64]. Crystallographic human TDP1 structures were downloaded from the RCSB Protein DataBank [73] (see Table S1) and prepared using the protein preparation module of Maestro [74] in interactive mode. In this process, redundant molecules were removed, missing atoms were added, bond orders and partial charges were assigned, H bonds were set, ionization and tautomeric states were assigned, and restrained minimization was performed. Receptor grids were generated using Maestro, where docking sites were defined as a cube of 27,000 Å^3^ with central coordinates provided in Table S1. Ligands were docked at 100 runs per ligand using the generated grids with Glide [75] in extra-precision mode [76]. Free binding energy (ΔG) values were calculated using the MM-GBSA panel of Maestro and Prime [77] for the selected ligand–receptor complexes, allowing sidechain flexibility for the residues within 4 Å of the docked ligand. Poses were visually evaluated using Maestro.

### 3.3. Biology

#### 3.3.1. Real-Time Detection of TDP1 Activity

16-Mer DNA oligonucleotide, as a biosensor (5′-[FAM] AAC GTC AGGGTC TTC C [BHQ]-3′), was synthesized in the Laboratory of Nucleic Acid Chemistry at the Institute of Chemical Biology and Fundamental Medicine (Novosibirsk, Russia). Real-time fluorescence detection of TDP1 enzyme activity was carried out as described [59]. The recombinant TDP1 was purified as described [78], using plasmid pET 16B-TDP1, kindly provided by Dr. K.W. Caldecott (University of Sussex, Brighton, UK). To the reaction mixture (200 µL) containing 50 nM oligonucleotide in buffer (50 mM Tris-HCl, pH 8.0, 50 mM NaCl, and 7 mM β-mercaptoethanol), we added varied concentrations of the tested compounds and purified TDP1 in a final concentration of 1.5 nM. The reaction was initiated by adding the enzyme. Fluorescence intensity (Ex485/Em520 nm) was measured using POLARstar OPTIMA fluorimeter (BMG LABTECH, GmbH, Ortenberg, Germany) every 1 min for 7 min. The half maximal inhibitory concentrations (IC_50_) were determined using a six-point concentration response curve and calculated using MARS Data Analysis 2.0 (BMG LABTECH). At least three independent experiments were carried out to obtain the IC_50_ values.

#### 3.3.2. Cytotoxicity Assay

The cytotoxicity of the compounds to HeLa (human cervical cancer) and A549 (human lung cancer) cell lines was determined using standard colorimetric MTT test [79]. Cell lines were obtained from the Russian Cell Culture Collection (RCCC) Institute of Cytology RAS (St. Petersburg, Russia). Cells were seeded in 96-well plates (5000 cells per well) and cultured in DMEM medium (Invitrogen, Carlsbad, CA, USA) supplemented with 10% fetal bovine serum (FBS) (Invitrogen), penicillin (100 units/mL), and streptomycin (100 µg/mL) at 37 °C and 5% CO_2_ in a humid atmosphere. At 30–50% confluence, the tested compounds were added to the medium. All measurements were repeated three times.

To determine the cytotoxicity of TDP1 inhibitors, the cells were allowed to attach for 24 h and treated with compounds with concentrations ranging from 1 to 100 µM for 72 h at 37 °C. Control wells contained 1% DMSO.

To study the effect of the compounds on the cytotoxicity of topotecan (United States Pharmacopeia, Rockville, MD, USA), an aqueous solution of topotecan was used at concentrations from 0.1 to 0.75 µM for HeLa cells and from 0.2 to 2.5 µM for A549 cells against the background of 10 µM of the compounds. Cells treated with compounds alone without topotecan were used as controls.

## 4. Conclusions

Two representative libraries of new conjugates were obtained: one containing a 1,2,3-triazole linker (obtained via an azide–alkyne cycloaddition reaction) and one containing an isoxazole linker (obtained via a [3 + 2] cycloaddition reaction of nitrile oxides). The nature of the heterocyclic bridge was found to play a key role in enzyme binding. Conjugates with an isoxazole linker (compounds **14a**–**17e**) exhibited comparatively higher inhibitory activity (IC_50_ < 1.0 μM for the leaders **15a**, **16a**, and **16b**) compared to their triazole-containing analogs. Molecular modeling pointed to the importance of the coumarin ring, which was predicted to target the catalytic site of TDP1. Comparative HPLC retention time analysis revealed that the new conjugates are less lipophilic than their precursors, an important factor for the further development of drug candidates. The identified lead compounds have low intrinsic cytotoxicity but at the same time effectively enhance the effect of topotecan on HeLa tumor cells.

## 5. Patents

Some of the compounds and approaches discussed in this work are related to Russian Federation Patent RU 2863125 C1, “7-Hydroxycoumarin derivatives conjugated with monoterpene residues through an isoxazole linker as TDP1 enzyme inhibitors and components of antitumor chemotherapy.”

## Figures and Tables

**Table 1:** Structure–anti-TDP1 activity relationship for conjugates **14a**–**17e** linked via an isoxazole linker. IC_50_ is expressed in µM. Color scale ranges from green (lowest values) to orange (highest values). Each cell represents a conjugate comprising the coumarin moiety shown in the corresponding column, isoxazole linker shown in the top left corner, and the monoterpene moiety shown in the corresponding row. Compound identifiers combine the column number and row letter (e.g., 14* and *a combine to form **14a**). Comparison drug: furamidine **2** (IC_50_ = 1.2 ± 0.3 µM).

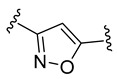	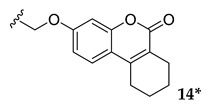	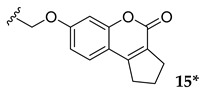	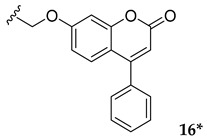	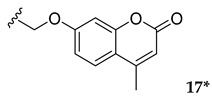
** 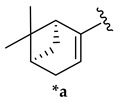 **	1.6 ± 0.4	0.9 ± 0.2	0.9 ± 0.5	2.7 ± 0.5
** 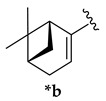 **	1.1 ± 0.4	1.2 ± 0.2	0.8 ± 0.01	2.3 ± 0.6
** 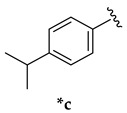 **	1.5 ± 0.2	9 ± 2	1.2 ± 0.3	3.2 ± 1.0
** 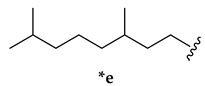 **	0.8 ± 0.5	1.6 ± 0.4	2.1 ± 0.2	3.1 ± 0.7

**Table 2:** Structure–anti-TDP1 activity relationships for conjugates **20a**–**23e** and **26a**–**e**, linked via a triazole linker. IC_50_ values are expressed in µM. Color scale ranges from green (lowest values) to red (highest values). Each cell represents a conjugate comprising the coumarin moiety shown in the corresponding column, triazole linker shown in the top left corner, and the monoterpene moiety shown in the corresponding row. Compound identifiers combine the column number and row letter (e.g., 20* and *a combine to form **20a**). The comparison drug is furamidine **2** (IC_50_ = 1.2 ± 0.3 µM).

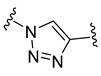	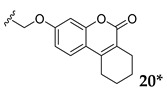	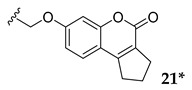	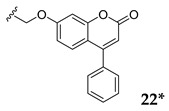	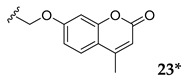	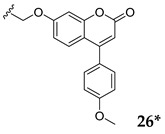
** 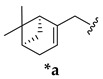 **	13.0 ± 1	14.3 ± 0.2	2.0 ± 0.2	>50	2.7 ± 0.3
** 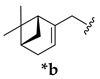 **	4.9 ± 0.7	4.7 ± 0.5	3.2 ± 0.4	23.3 ± 0.4	2.2 ± 0.2
** 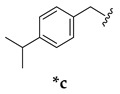 **	2.7 ± 0.4	4.5 ± 0.2	1.1 ± 0.3	12.0 ± 0.3	1.9 ± 0.2
** 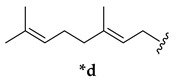 **	5.1 ± 0.2	3.2 ± 0.4	2.8 ± 0.4	13.5 ± 0.4	2 ± 0.2
** 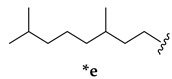 **	1.8 ± 0.1	1.7 ± 0.1	1.5 ± 0.4	5 ± 1	1.7 ± 0.4

**Table 3:** Toxicity of compounds **21d**, **26e**, and **16b** relative to HeLa and A549 cell lines.

Compound	CC_50_ (HeLa), µM	CC_50_ (A549 Wild Type), µM
**21d**	24 ± 4	68 ± 1
**21d + TPC**	0.32 ± 0.05	
**26e**	>100	>100
**26e + TPC**	0.31 ± 0.05	
**16b**	>100	>100
**16b + TPC**	0.24 ± 0.04	
**TPC**	0.4 ± 0.06	2.5 ± 0.5

## Data Availability

The original contributions presented in the study are included in the article/Supplementary Materials; further inquiries can be directed to the corresponding author.

## Supplementary Materials

The following supporting information can be downloaded at https://www.mdpi.com/article/10.3390/ijms27146421/s1. HRMS, ^1^H, and ^13^C spectra of the compounds; data on docking studies—protein ligand complexes used, R^2^ values, and docking scores; calculated bioavailability radars of the compounds; 2D interaction diagrams of compounds **14b**, **14d**, **15a**, **16a**, **16b**, and **16c, 22a**–**c, 23a**–**c** at the human TDP1 active site; and HPLC retention time diagrams of compounds varying only by the heterocyclic linker. References [80,81,82,83,84,85,86,87,88,89] are cited in the supplementary materials.
