# Supplementary material for: Synthesis and Biological Evaluation of TDP1 Inhibitors Based on Coumarin and Monoterpenoid Fragments Conjoined by Heterocyclic Moieties
Source: Int J Mol Sci. 2026 Jul 19;27(14):6421. doi: 10.3390/ijms27146421 (PMC13409999; doi:10.3390/ijms27146421)

Supplementary Information

Synthesis and biological evaluation of TDP1 inhibitors based on coumarin and monoterpenoid fragments conjoined by heterocyclic moieties

1. NMR ^{1}H and ^{13}C spectra of the compounds 26a-26e; 14a-c, 15a-c, 16a-c, 17a-c, 13e.
2. HRMS spectra of the compounds 26a-26e; 14a-c, 15a-c, 16a-c, 17a-c, 13e.
3. ^{1}H NMR spectra (ppm from 8.4 to 5.3) of the 12b dimerization recorded with a 3-minute delay at 200 MHz resolution.
4. Crystallographic structures of human TDP1 used in this study and validation of the docking process
5. Prediction of druglikeness
6. Electrostatic and van der Waals interactions of the compounds with TDP1 as predicted through molecular docking
7. HPLC Retention times of compounds varying in the heterocyclic linker only.

1. NMR ^{1}H and ^{13}C spectra of the compounds 26a-26e; 14a-c, 15a-c, 16a-c, 17a-c, 13e.

Figure S1. ^{1}H spectra of Compound **26a**

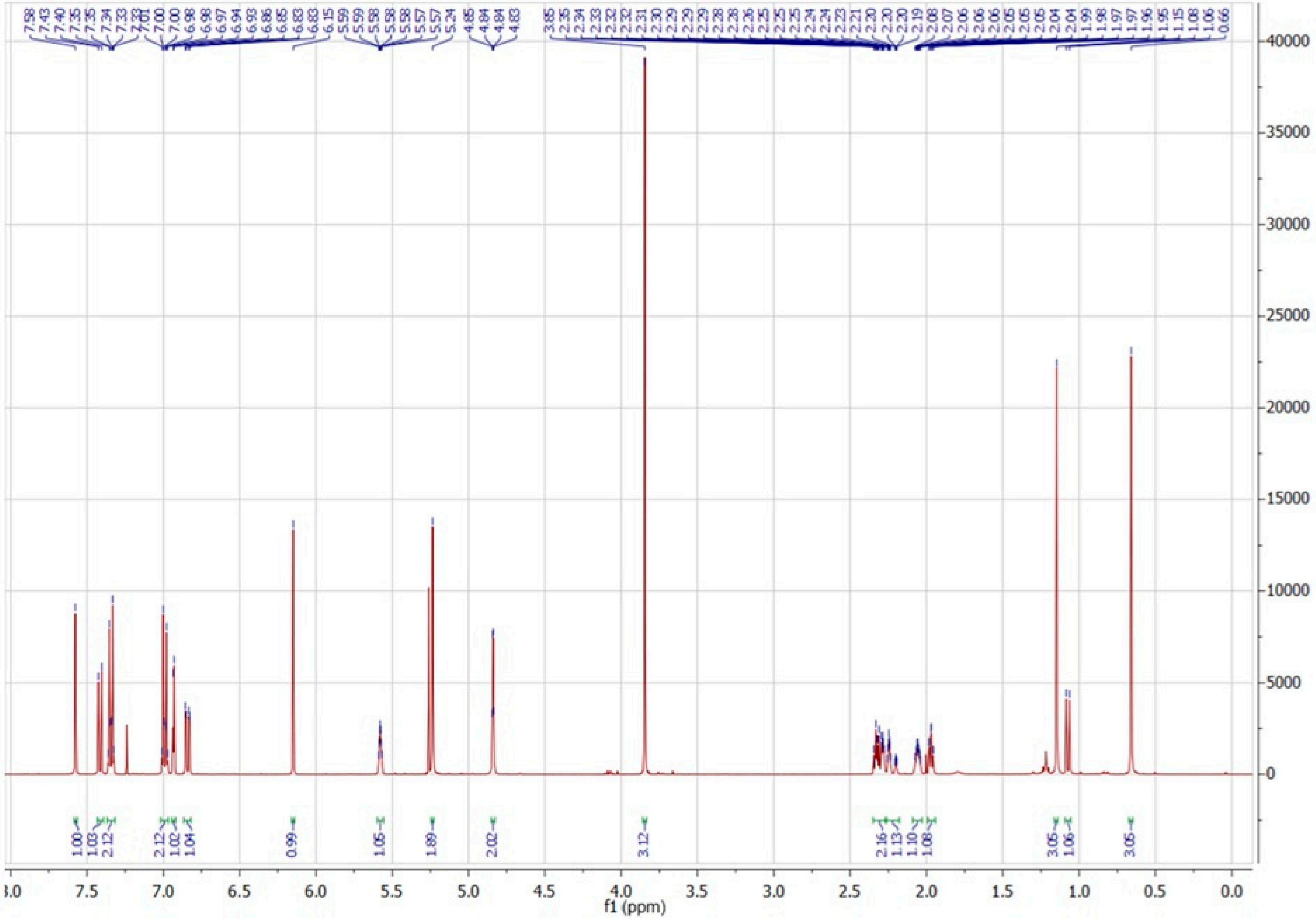

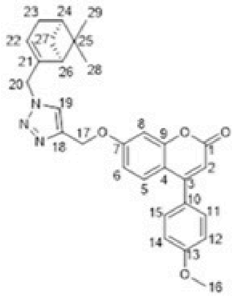

^{1}H NMR (400 MHz, $CDCl_3$) δ 7.58 (s, 1H, H-19), 7.42 (d, *J* = 8.9 Hz, 1H, H-5), 7.34 (dt, *J* = 8.8, 3.0 Hz, 2H, H-11,15), 6.98 (dt, *J* = 9.0, 3.0 Hz, 2H, H-12,14), 6.93 (d, *J* = 2.5 Hz, 1H, H-8), 6.84 (dd, *J* = 8.9, 2.5 Hz, 1H, H-6), 6.15 (s, 1H, H-2), 5.58 (hept, *J* = 1.5 Hz, 1H, H-22), 5.24 (s, 2H, H-17), 4.84 (q, *J* = 1.5 Hz, 2H, H-20), 3.85 (s, 3H, H-16), 2.32 (dt, *J* = 8.8, 5.6 Hz, 1H, H-27b), 2.24 (qh, *J* = 8.8, 1.5 Hz, 2H, H-23), 2.06 (hept, *J* = 2.9, 1.6 Hz, 1H, H-24), 1.97 (td, *J* = 5.6, 1.5 Hz, 1H, H-26), 1.15 (s, 3H, H-29), 1.07 (d, *J* = 8.8 Hz, 1H, H-27a), 0.66 (s, 3H, H-28).

Figure S2. ^{13}C spectra of Compound **26a**.

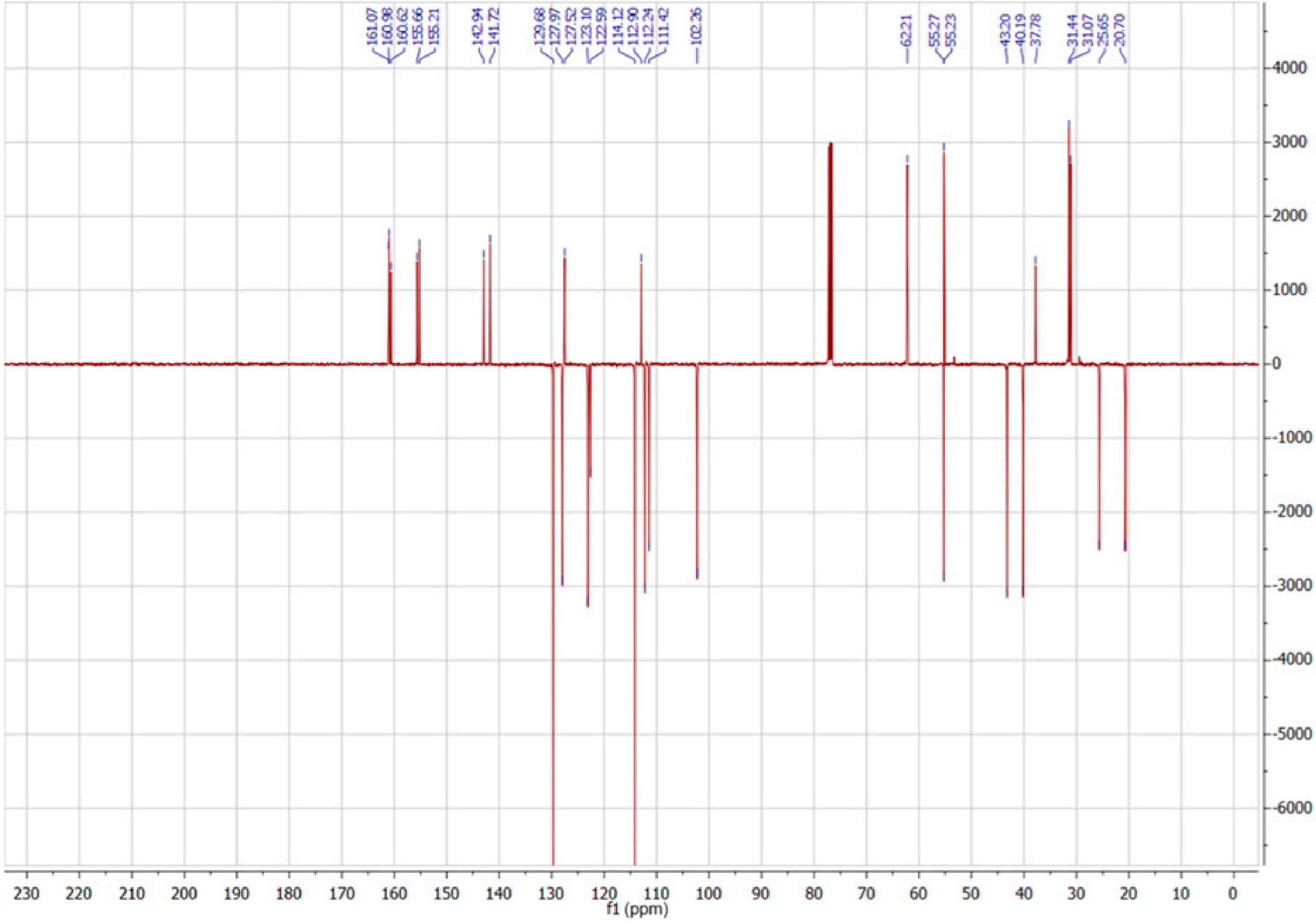

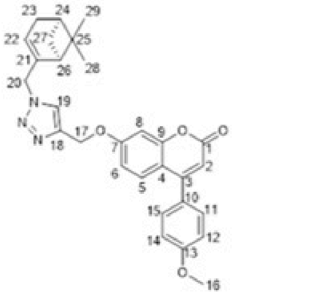

^{13}C NMR (101 MHz, $CDCl_3$) δ 161.07 (s, C-7), 160.98 (s, C-1), 160.62 (s, C-9), 155.66 (s, C-3), 155.21 (s, C-13), 142.94 (s, C-18), 141.72 (s, C-21), 129.68 (d, 2C, C-11,15), 127.97 (d, 2C, C-12,14), 127.52 (s, C-10), 123.10 (d, C-22), 122.59 (d, C-19), 114.13 (d, C-5), 112.90 (s, C-4), 112.24 (d, C-6), 111.42 (d, C-2), 102.26 (d, C-8), 62.21 (t, C-17), 55.27 (q, C-16), 55.23 (t, C-20), 43.20 (d, C-26), 40.19 (d, C-24), 37.78 (s, C-25), 31.44 (t, C-27), 31.07 (t, C-23), 25.65 (q, C-29), 20.70 (q, C-28).

Figure S3. ^{1}H spectra of Compound **26c**

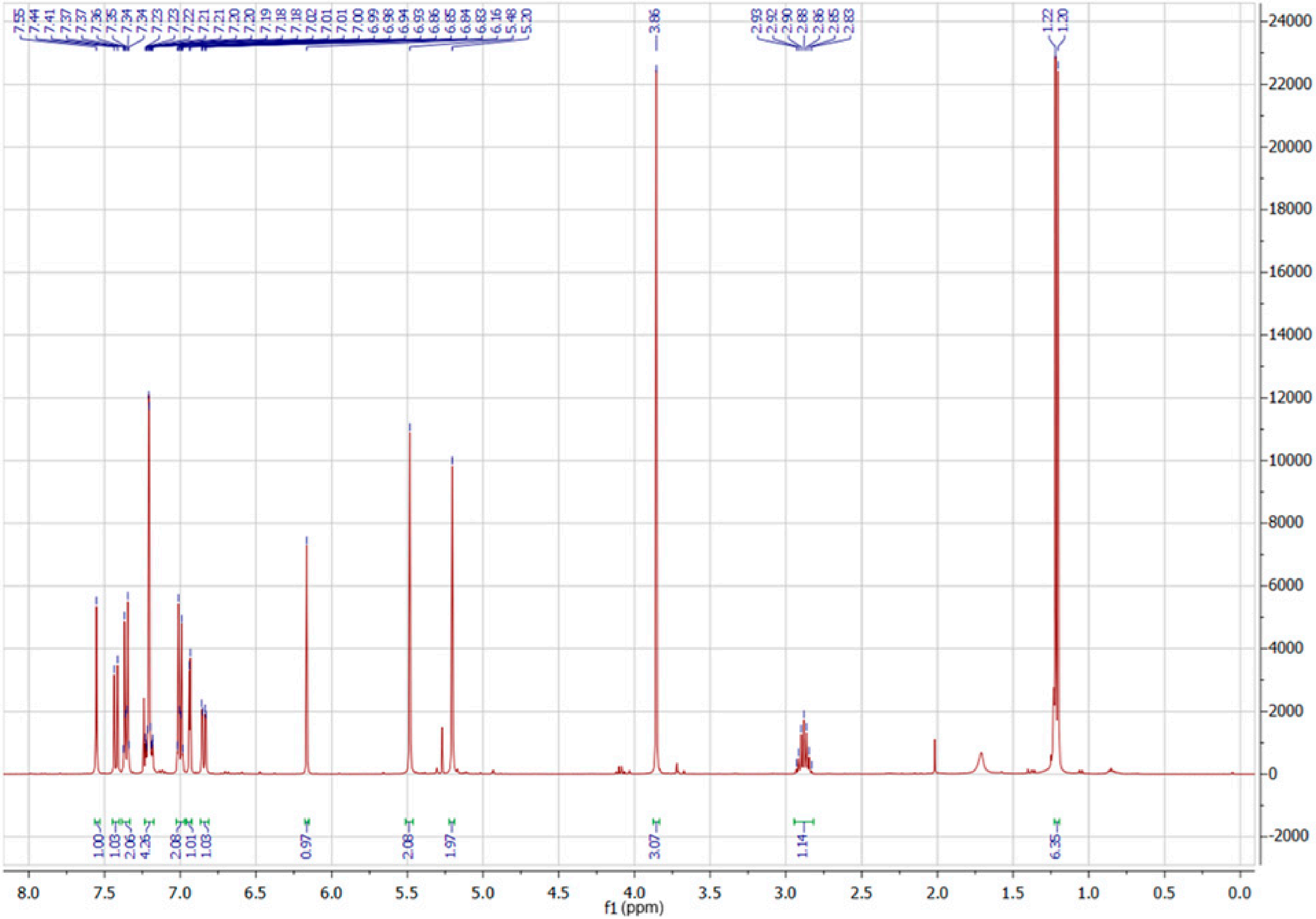

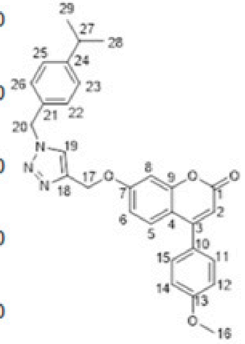

^{1}H NMR (400 MHz, $CDCl_3$) δ 7.55 (s, 1H, H-19), 7.42 (d, *J* = 8.8 Hz, 1H, H-5), 7.33 – 7.38 (m, 2H, H-11,15), 7.18 – 7.23 (m, 4H, H-22,23,25,26), 6.97 – 7.02 (m, 2H, H-12,14), 6.94 (d, *J* = 2.6 Hz, 1H, H-8), 6.84 (dd, *J* = 8.8, 2.6 Hz, 1H, H-6), 6.16 (s, 1H, H-2), 5.48 (s, 2H, H-20), 5.20 (s, 2H, H-17), 3.86 (s, 3H, H-16), 2.88 (p, *J* = 6.9 Hz, 1H, H-27), 1.21 (d, *J* = 6.9 Hz, 6H, H-28,29).

Figure S4. ^{13}C spectra of Compound **26c**

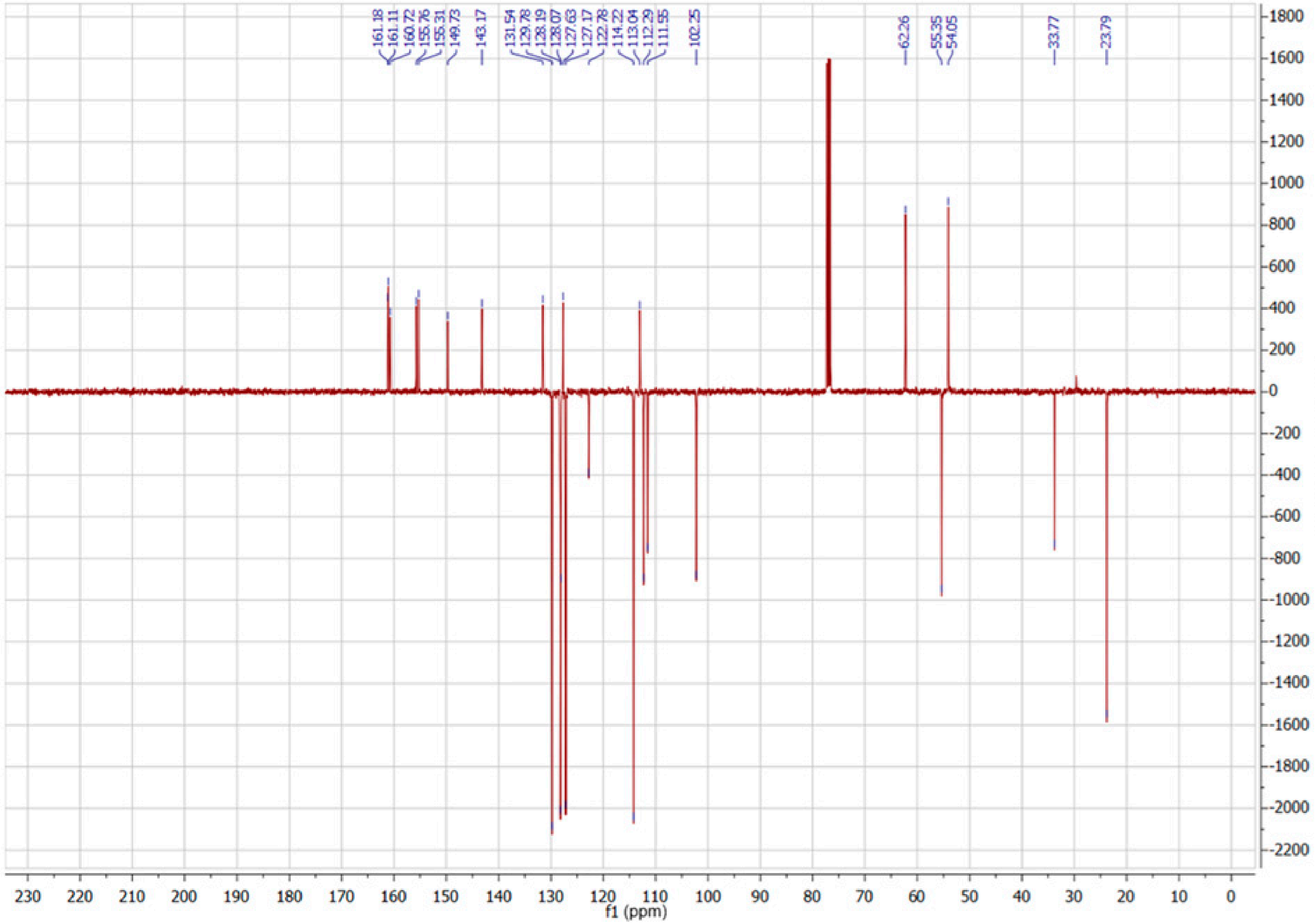

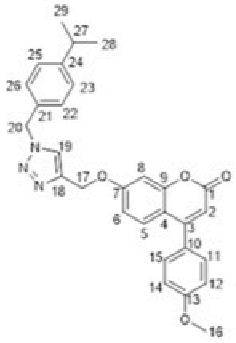

^{13}C NMR (101 MHz, $CDCl_3$) δ 161.18 (s, C-7), 161.11 (s, C-1), 160.72 (s, C-9), 155.76 (s, C-3), 155.31 (s, C-10), 149.73 (s, C-24), 143.17 (s, C-18), 131.54 (s, C-21), 129.78 (d, 2C, C-11,15), 128.19 (d, 2C, C-23,25), 128.07 (d, 2C, C-12,14), 127.63 (s, C-13), 127.17 (d, 2C, C-22,26), 122.79 (d, C-19), 114.22 (d, C-5), 113.04 (s, C-4), 112.29 (d, C-6), 111.55 (d, C-2), 102.25 (d, C-8), 62.26 (t, C-17), 55.35 (q, C-16), 54.05 (t, C-20), 33.77 (d, C-27), 23.79 (q, 2C, C-28,29).

Figure S5. ^{1}H spectra of Compound **26d-*E***

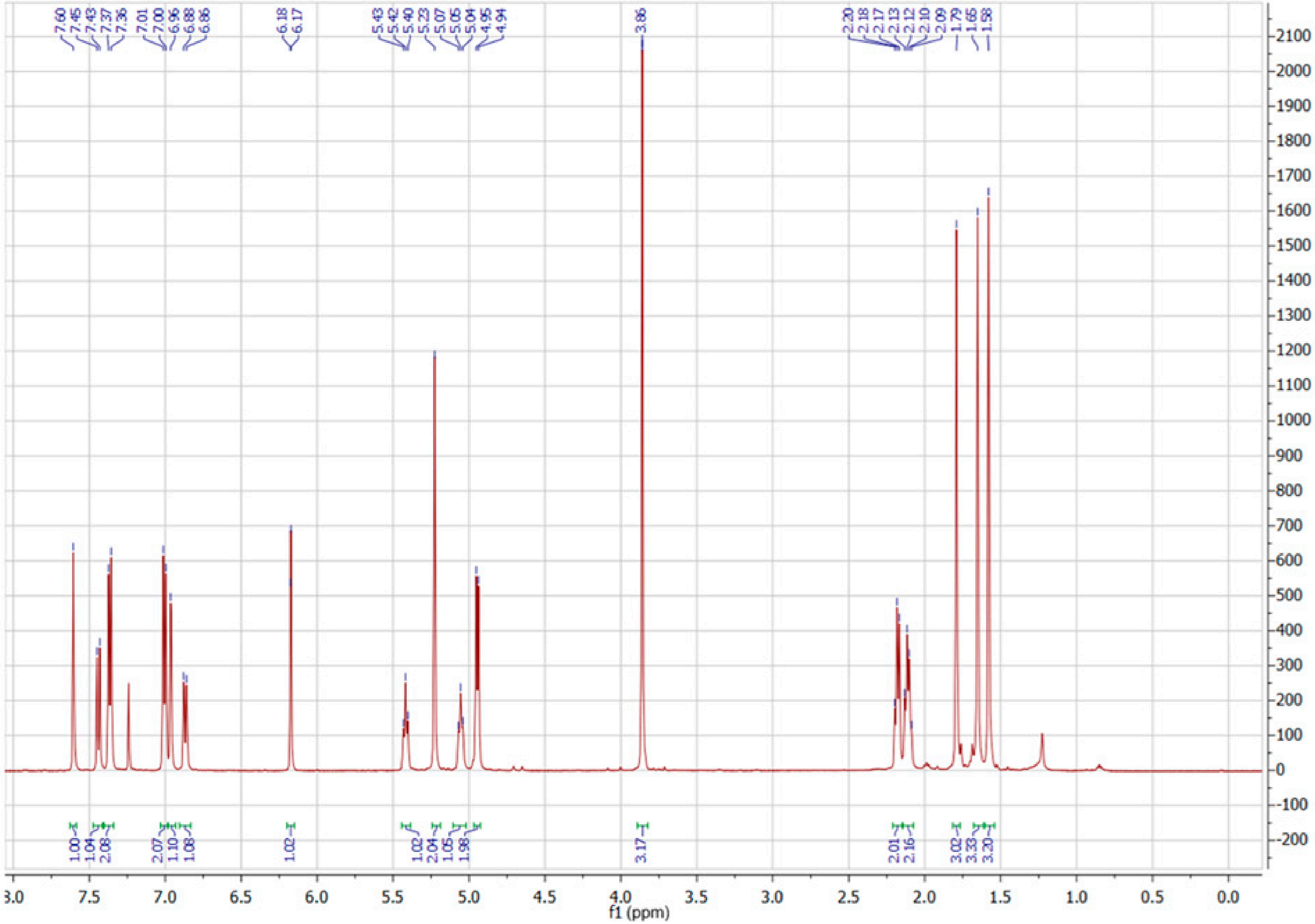

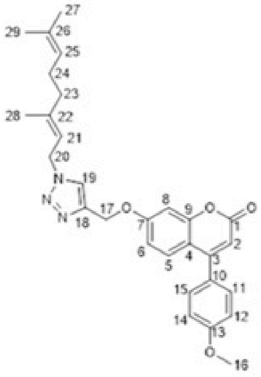

^{1}H NMR (400 MHz, $CDCl_3$) δ 7.60 (s, 1H, H-19), 7.44 (d, J = 8.9 Hz, 1H, H-5), 7.36 (d, J = 8.4 Hz, 2H, H-11,15), 7.00 (d, J = 8.4 Hz, 2H, H-12,14), 6.97 (s, 1H, H-8), 6.87 (d, J = 8.9 Hz, 1H, H-6), 6.17 (s, 1H, H-2), 5.42 (t, J = 7.4 Hz, 1H, H-21), 5.23 (s, 2H, H-17), 5.05 (t, J = 7.3 Hz, 1H, H-25), 4.94 (d, J = 7.4 Hz, 2H, H-20), 3.86 (s, 3H, H-16), 2.18 (t, J = 7.3 Hz, 2H, H-23), 2.11 (q, J = 7.3 Hz, 2H, H-24), 1.79 (s, 3H, H-28), 1.65 (s, 3H, H-29), 1.58 (s, 3H, H-27).

Figure S6. ^{13}C spectra of Compound **26d-*E***

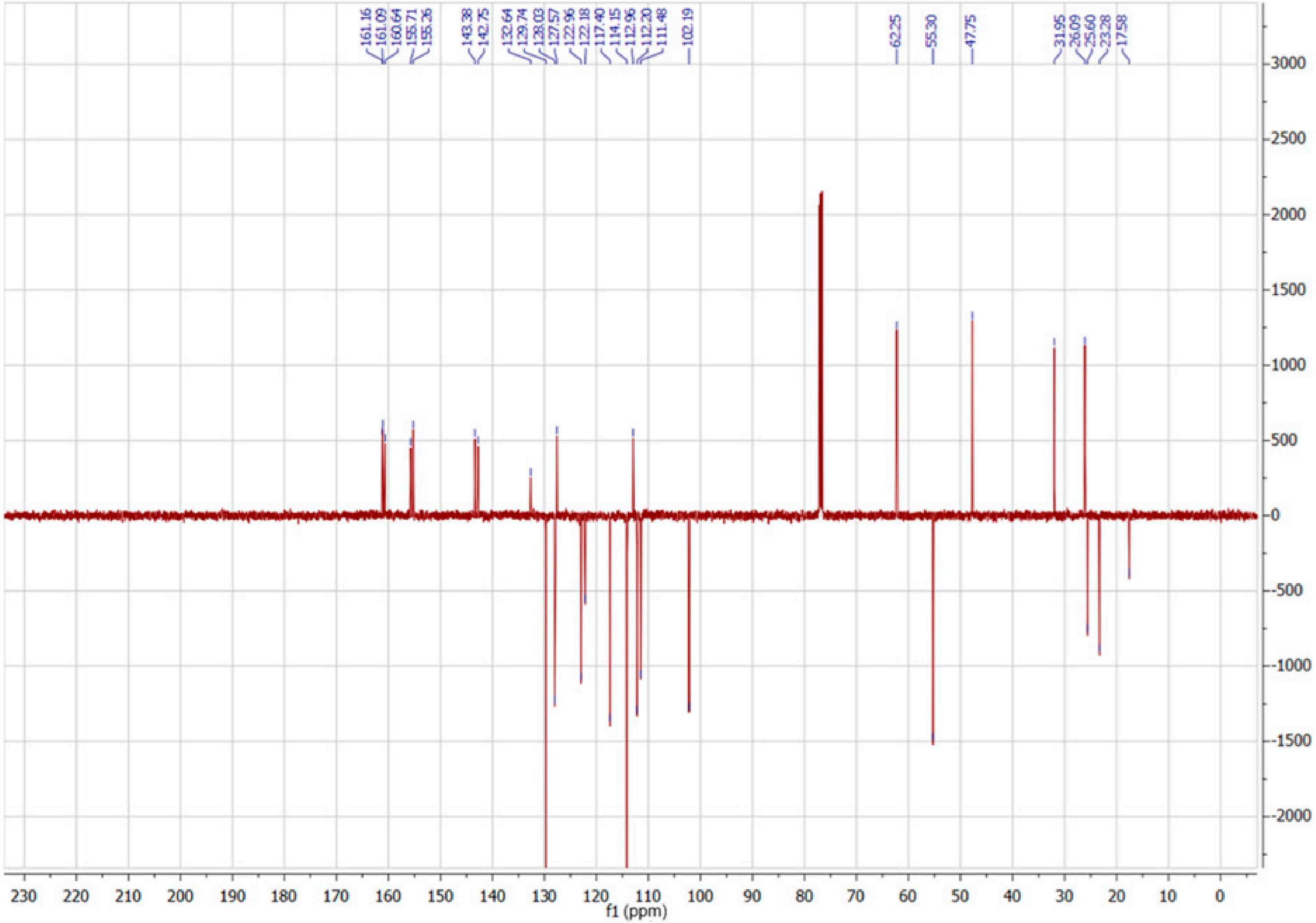

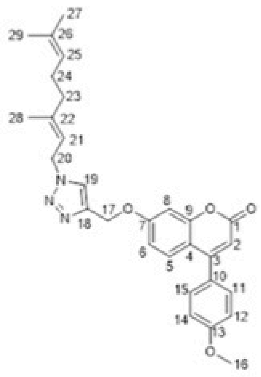

^{13}C NMR (101 MHz, $CDCl_3$) δ 161.16 (s, C-7), 161.09 (s, C-1), 160.64 (s, C-9), 155.71 (s, C-3), 155.26 (s, C-13), 143.38 (s, C-22), 142.75 (s, C-18), 132.64 (s, C-26), 129.74 (d, 2C, C-11,15), 128.03 (d, 2C, C-12,14), 127.57 (s, C-10), 122.96 (d, C-25), 122.18 (d, C-19), 117.40 (d, C-21), 114.15 (d, C-5), 112.96 (s, C-4), 112.20 (d, C-6), 111.48 (d, C-2), 102.20 (d, C-8), 62.25 (t, C-17), 55.30 (q, C-16), 47.75 (t, C-20), 31.95 (t, C-23), 26.09 (t, C-24), 25.60 (q, C-29), 23.28 (q, C-28), 17.58 (q, C-27).

Figure S7. ^{1}H spectra of Compound **26d-*Z***

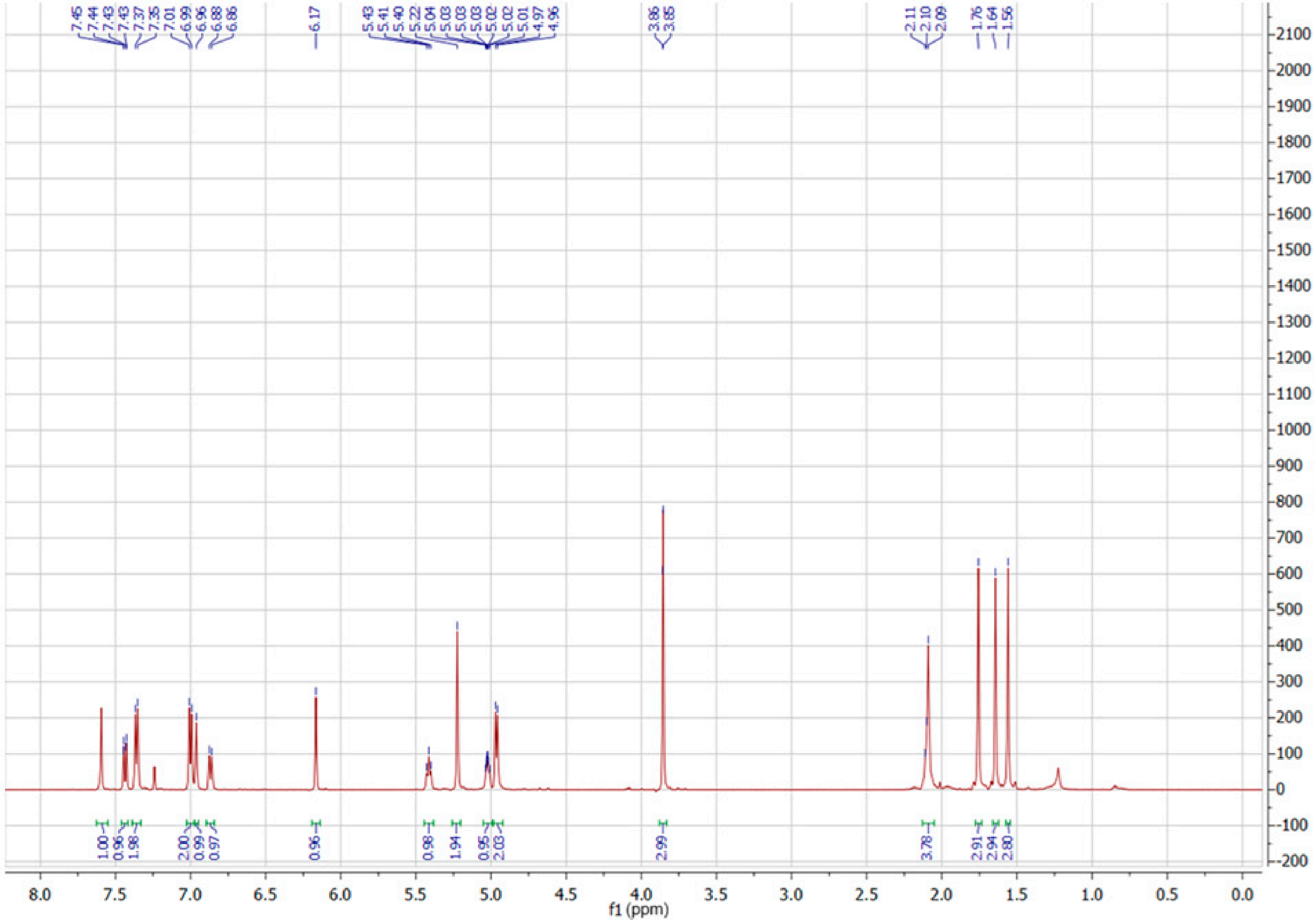

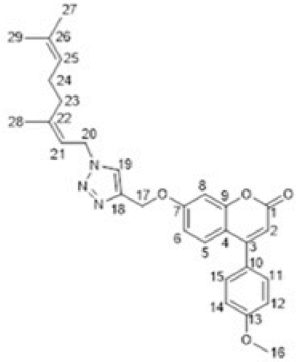

^{1}H NMR (400 MHz, $CDCl_3$) δ 7.59 (s, 1H, H-19), 7.44 (d, *J* = 9.0 Hz, 1H, H-5), 7.36 (d, *J* = 8.8 Hz, 2H, H-11,15), 7.00 (d, *J* = 8.8 Hz, 2H, H-12,14), 6.96 (s, 1H, H-8), 6.87 (d, *J* = 9.0 Hz, 1H, H-6), 6.17 (s, 1H, H-2), 5.41 (t, *J* = 7.4 Hz, 1H, H-21), 5.22 (s, 2H, H-17), 5.00 – 5.05 (m, 1H, H-25), 4.96 (d, *J* = 7.3 Hz, 2H, H-20), 3.86 (s, 3H, H-16), 2.09 (s, 4H, H-23,24), 1.76 (s, 3H, H-27), 1.64 (s, 3H, H-28), 1.56 (s, 3H, H-29).

Figure S8. ^{13}C spectra of Compound **26d-*Z***

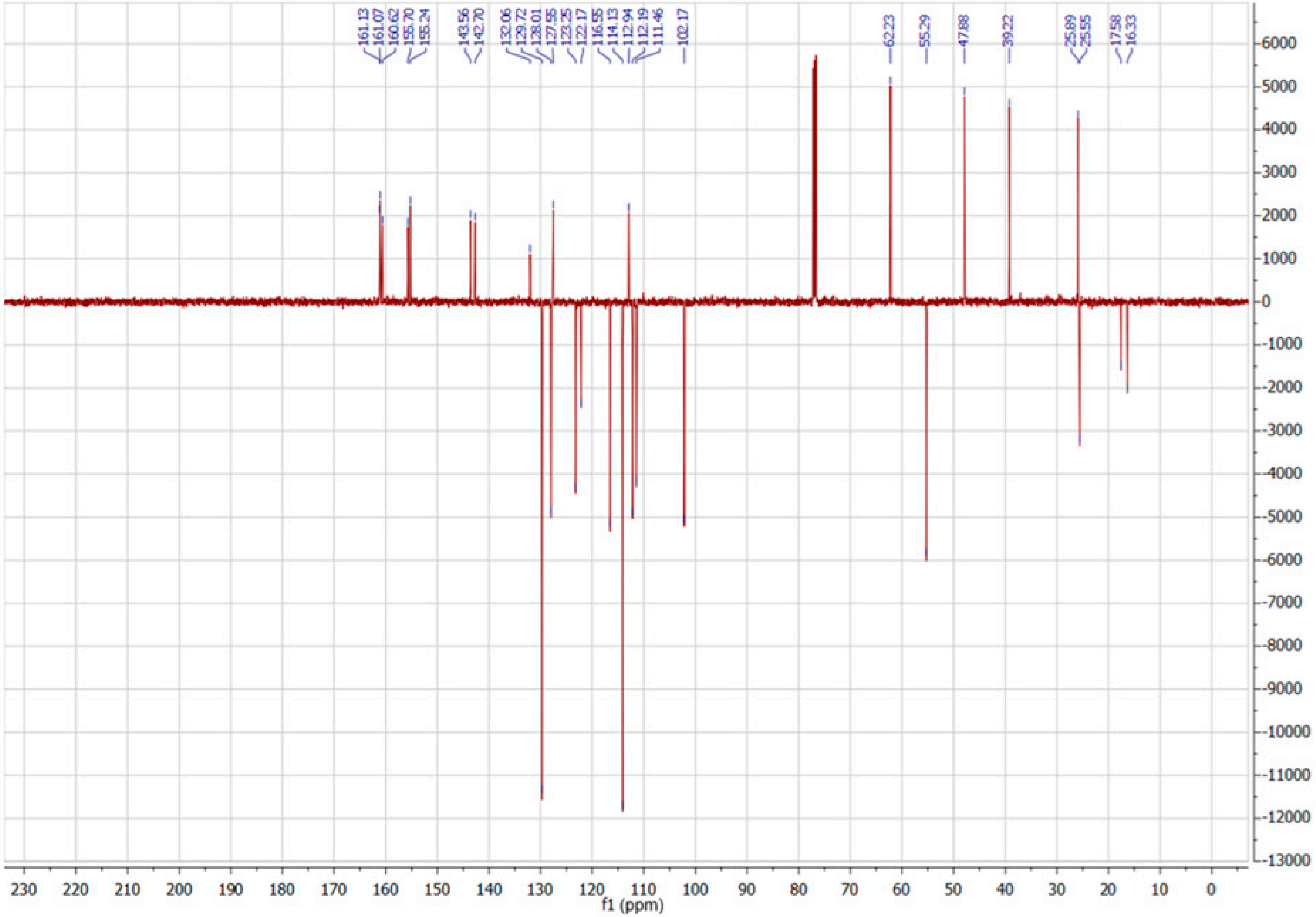

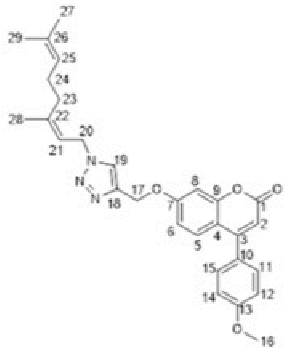

^{13}C NMR (101 MHz, $CDCl_3$) δ 161.13 (s, C-7), 161.07 (s, C-1), 160.62 (s, C-9), 155.70 (s, C-3), 155.24 (s, C-13), 143.56 (s, C-22), 142.70 (s, C-18), 132.06 (s, C-26), 129.72 (d, 2C, C-11,15), 128.01 (d, 2C, C-12,14), 127.55 (s, C-10), 123.25 (d, C-25), 122.17 (d, C-19), 116.55 (d, C-21), 114.13 (d, C-5), 112.94 (s, C-4), 112.19 (d, C-6), 111.46 (d, C-2), 102.17 (d, C-8), 62.23 (t, C-17), 55.29 (q, C-16), 47.88 (t, C-20), 39.22 (t, C-23), 25.89 (t, C-24), 25.55 (q, C-29), 17.58 (q, C-27), 16.33 (q, C-28).

Figure S9. ^{1}H spectra of Compound **26e**

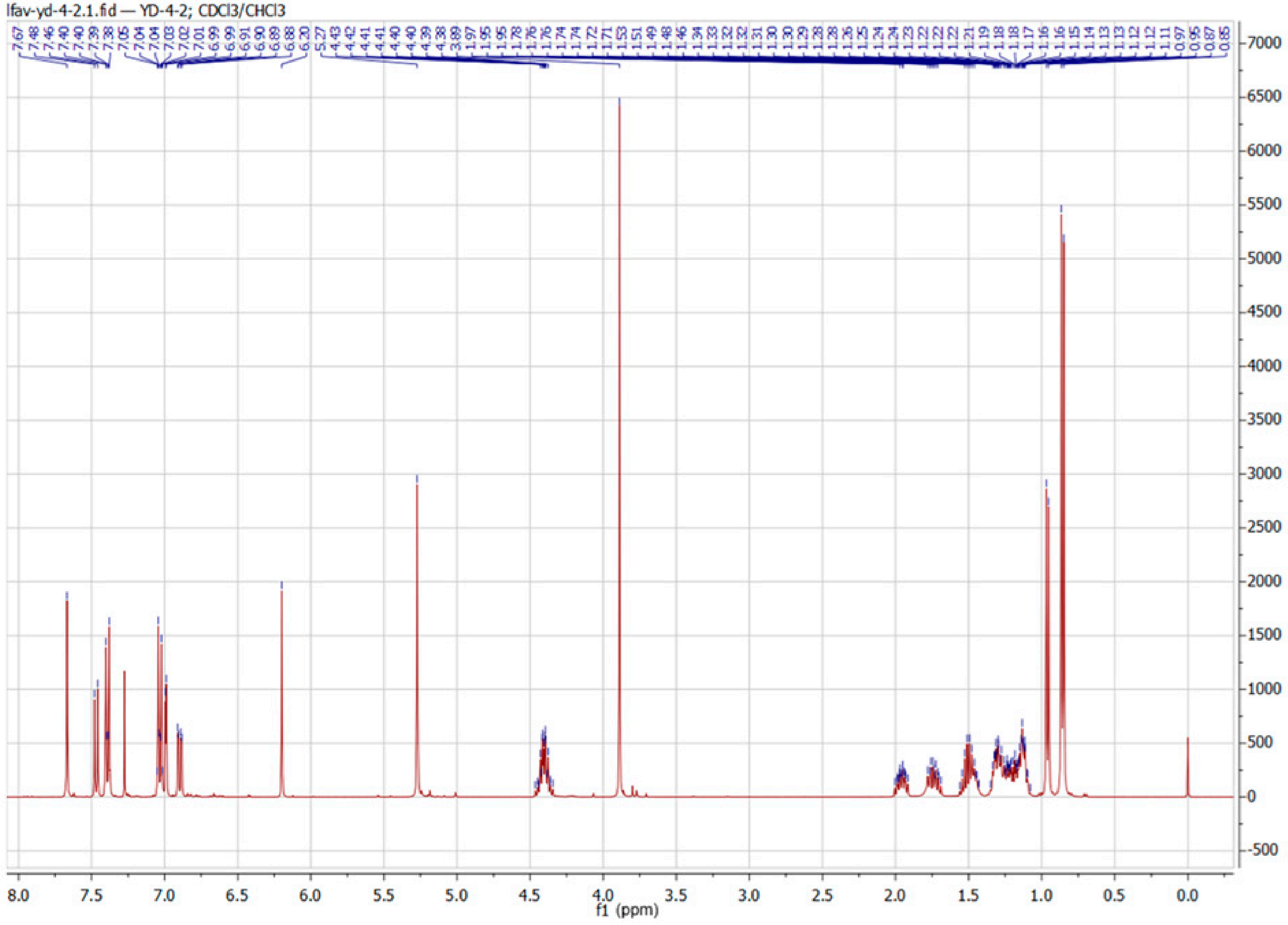

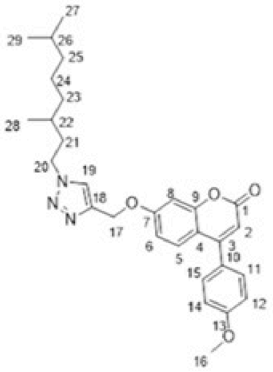

^{1}H NMR (400 MHz, $CDCl_3$) δ 7.67 (s, 1H, H-19), 7.47 (d, *J* = 8.9 Hz, 1H, H-5), 7.36 – 7.42 (m, 2H, H-11,15), 7.00 – 7.06 (m, 2H, H-12,14), 6.99 (d, *J* = 2.5 Hz, 1H, H-8), 6.90 (dd, *J* = 9.0, 2.5 Hz, 1H, H-6), 6.20 (s, 1H, H-2), 5.27 (s, 2H, H-17), 4.33 – 4.49 (m, 2H, H-20), 3.89 (s, 3H, H-16), 1.87 – 2.04 (m, 1H, H-21b), 1.64 – 1.83 (m, 1H, H-21a), 1.40 – 1.57 (m, 2H, H-22,26), 1.11 – 1.34 (m, 6H, H-23,24,25), 0.96 (d, *J* = 6.7 Hz, 3H, H-28), 0.86 (d, *J* = 6.7 Hz, 6H, H-27,29).

Figure S10. ^{13}C spectra of Compound **26e**

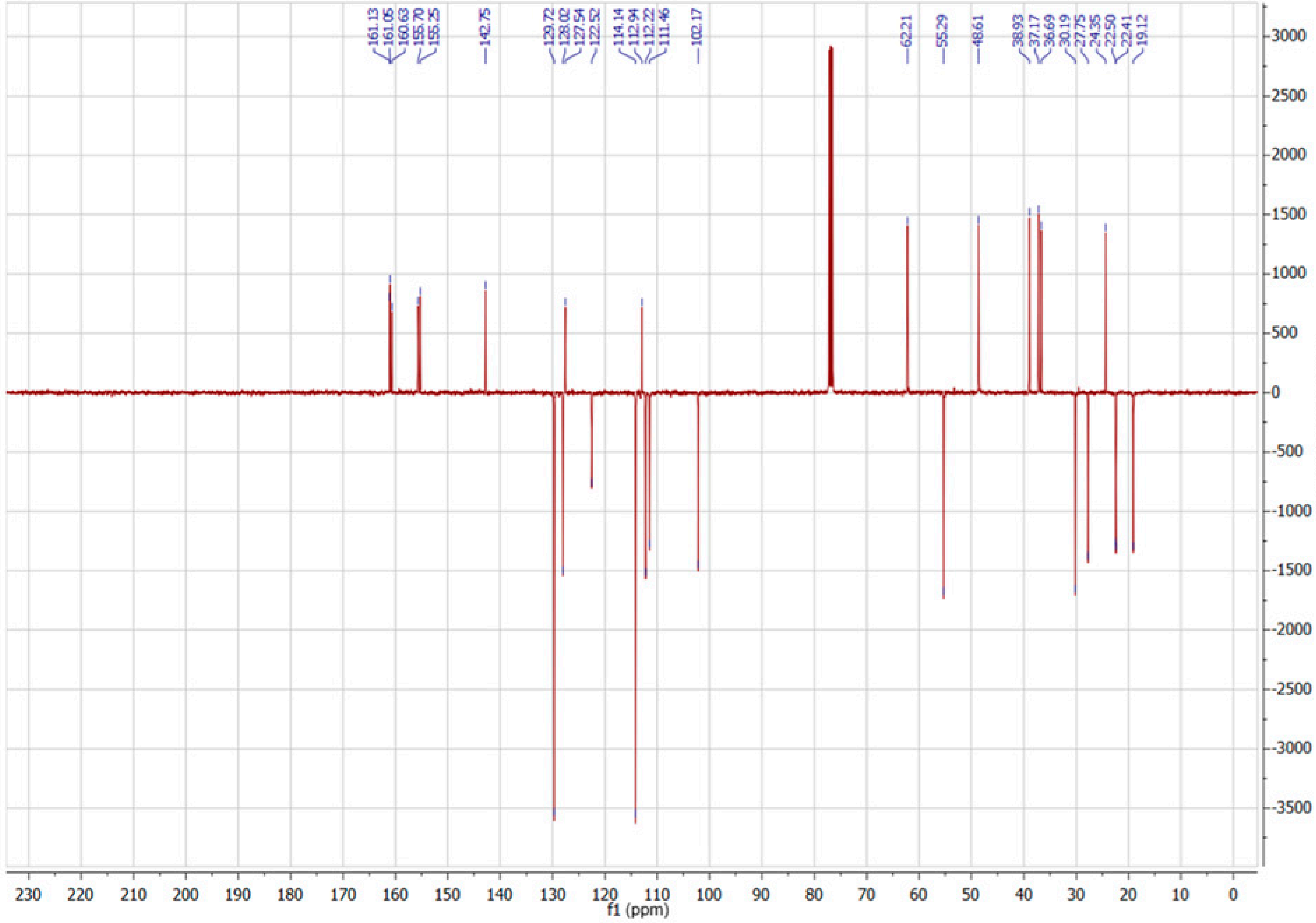

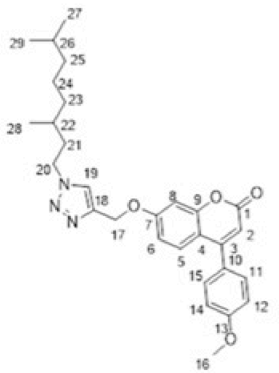

^{13}C NMR (101 MHz, $CDCl_3$) δ 161.14 (s, C-7), 161.05 (s, C-1), 160.63 (s, C-9), 155.70 (s, C-3), 155.25 (s, C-13), 142.75 (s, C-18), 129.72 (d, 2C, C-11,15), 128.02 (d, 2C, C-12,14), 127.54 (s, C-10), 122.52 (d, C-19), 114.14 (d, C-5), 112.95 (s, C-4), 112.22 (d, C-6), 111.46 (d, C-2), 102.17 (d, C-8), 62.21 (t, C-17), 55.29 (q, C-16), 48.61 (t, C-20), 38.93 (t, C-21), 37.17 (t, C-23), 36.69 (t, C-25), 30.19 (d, C-22), 27.75 (d, C-26), 24.35 (t, C-24), 22.50 (q, C-29), 22.41 (q, C-27), 19.12 (q, C-28).

Figure S11. ^{1}H spectra of Compound **14a**

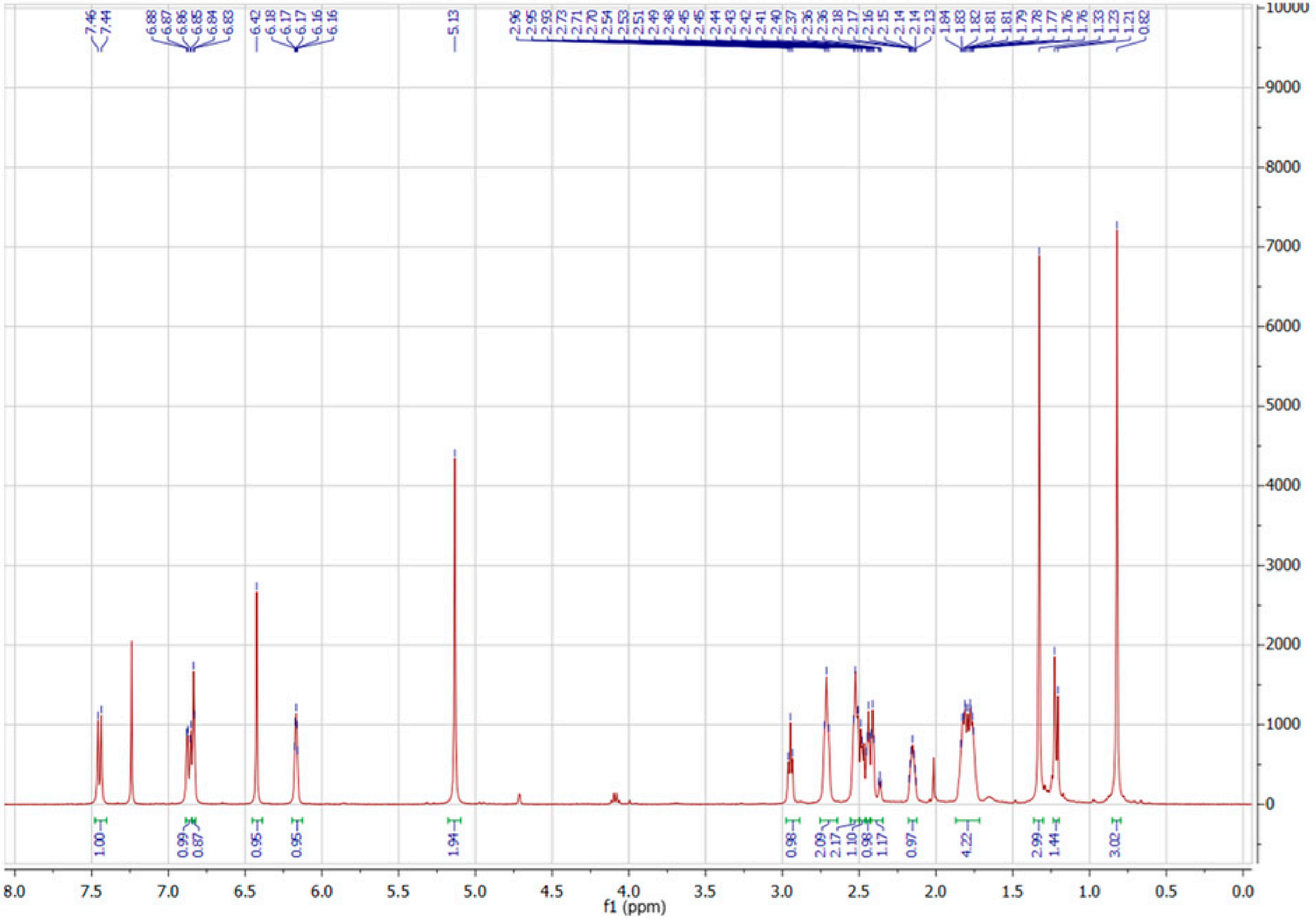

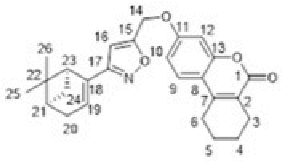

^{1}H NMR (400 MHz, $CDCl_3$) δ 7.45 (d, *J* = 9.0 Hz, 1H, H-9), 6.87 (dd, *J* = 9.0, 2.6 Hz, 1H, H-10), 6.83 (d, *J* = 2.6 Hz, 1H, H-12), 6.42 (s, 1H, H-16), 6.14 – 6.20 (m, 1H, H-19), 5.13 (s, 2H, H-14), 2.95 (t, *J* = 5.7 Hz, 1H, H-23), 2.71 (t, *J* = 6.5 Hz, 2H, H-6), 2.53 (t, *J* = 6.5 Hz, 2H, H-3), 2.44 – 2.51 (m, 1H, H-24b), 2.35 – 2.50 (m, 2H, H-20), 2.13 – 2.17 (m, 1H, H-21), 1.71 – 1.87 (m, 4H, H-4, H-5), 1.33 (s, 3H, H-26), 1.22 (d, *J* = 8.7 Hz, 1H, H-24a), 0.82 (s, 3H, H-25).

Figure S12. ^{13}C spectra of Compound **14a**

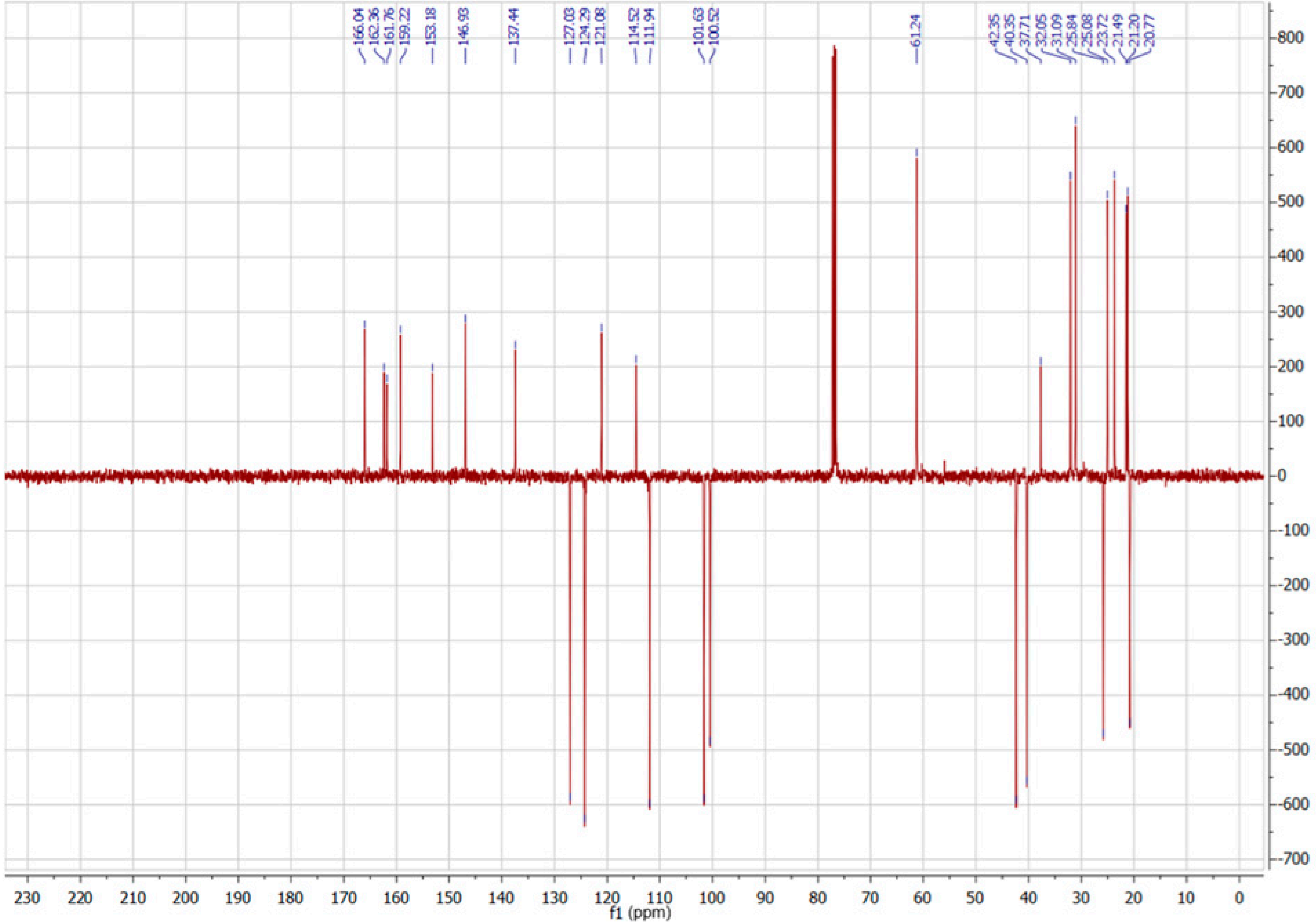

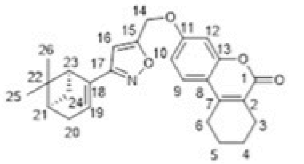

^{13}C NMR (101 MHz, $CDCl_3$) δ 166.04 (s, C-15), 162.36 (s, C-17), 161.77 (s, C-1), 159.22 (s, C-11), 153.18 (s, C-13), 146.93 (s, C-7), 137.44 (s, C-18), 127.03 (d, C-9), 124.29 (d, C-19), 121.08 (s, C-2), 114.52 (s, C-8), 111.94 (d, C-10), 101.63 (d, C-16), 100.52 (d, C-12), 61.24 (t, C-14), 42.35 (d, C-23), 40.35 (d, C-21), 37.71 (s, C-22), 32.05 (t, C-24), 31.09 (t, C-20), 25.84 (q, C-26), 25.08 (t, C-6), 23.72 (t, C-3), 21.49 (t, C-5), 21.20 (t, C-4), 20.77 (q, C-25).

Figure S13. ^{1}H spectra of Compound **14c**

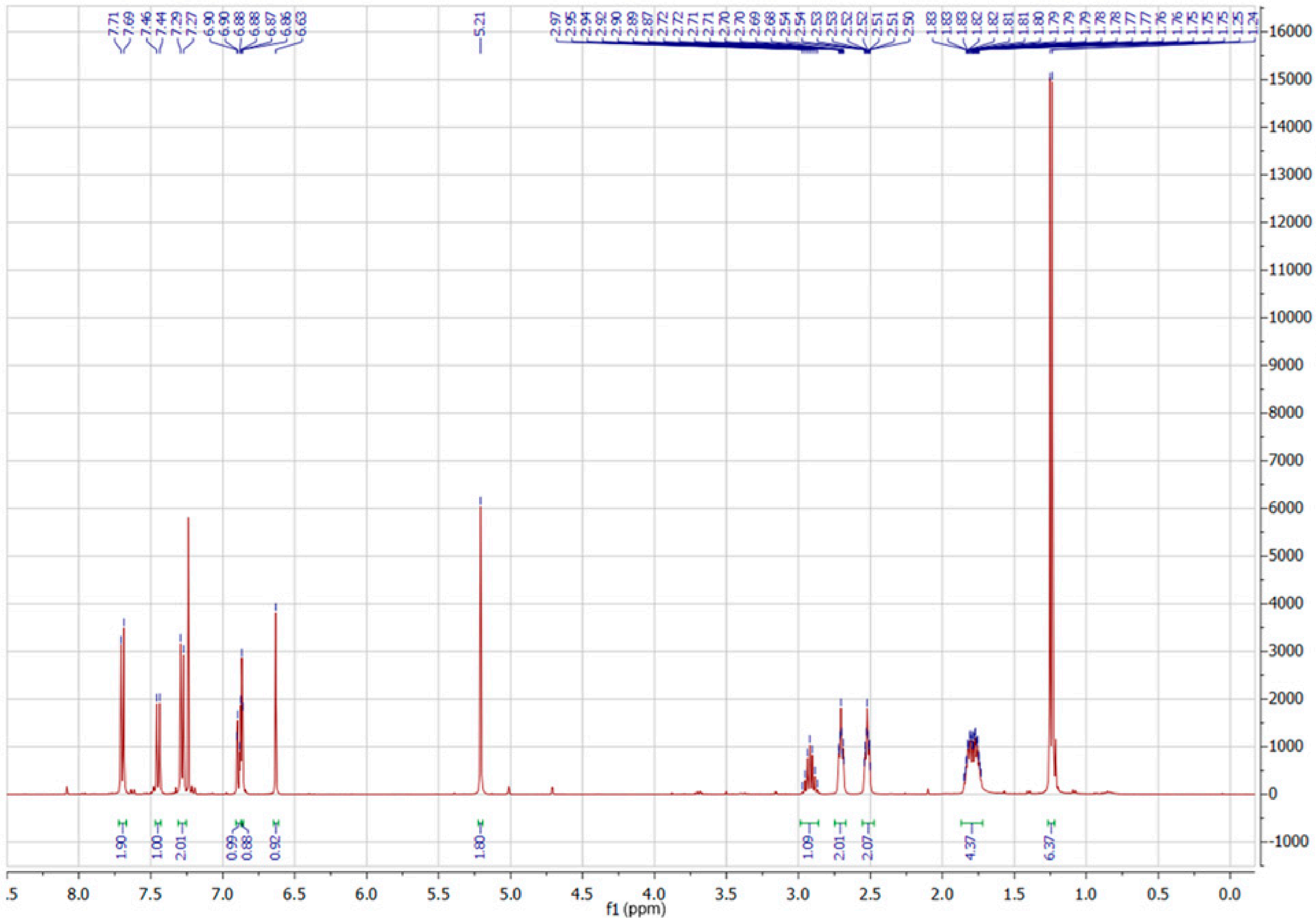

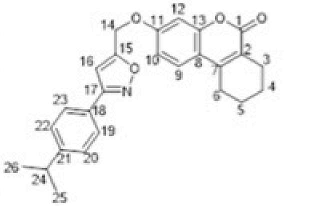

^{1}H NMR (400 MHz, $CDCl_3$) δ 7.70 (dt, *J* = 8.2, 1.7 Hz, 2H, H-19,23), 7.45 (d, *J* = 8.6 Hz, 1H, H-9), 7.28 (dt, *J* = 8.2, 1.7 Hz, 2H, H-20,22), 6.89 (dd, *J* = 8.6, 2.4 Hz, 1H, H-10), 6.86 (d, *J* = 2.4 Hz, 1H, H-12), 6.63 (s, 1H, H-16), 5.21 (s, 2H, H-14), 2.92 (h, *J* = 6.9 Hz, 1H, H-24), 2.71 (tt, *J* = 6.1, 2.0 Hz, 2H, H-6), 2.52 (tt, *J* = 6.1, 2.0 Hz, 2H, H-3), 1.70 – 1.88 (m, 4H, H-4, H-5), 1.24 (d, *J* = 6.9 Hz, 6H, H-25,26).

Figure S14. ^{13}C spectra of Compound **14c**

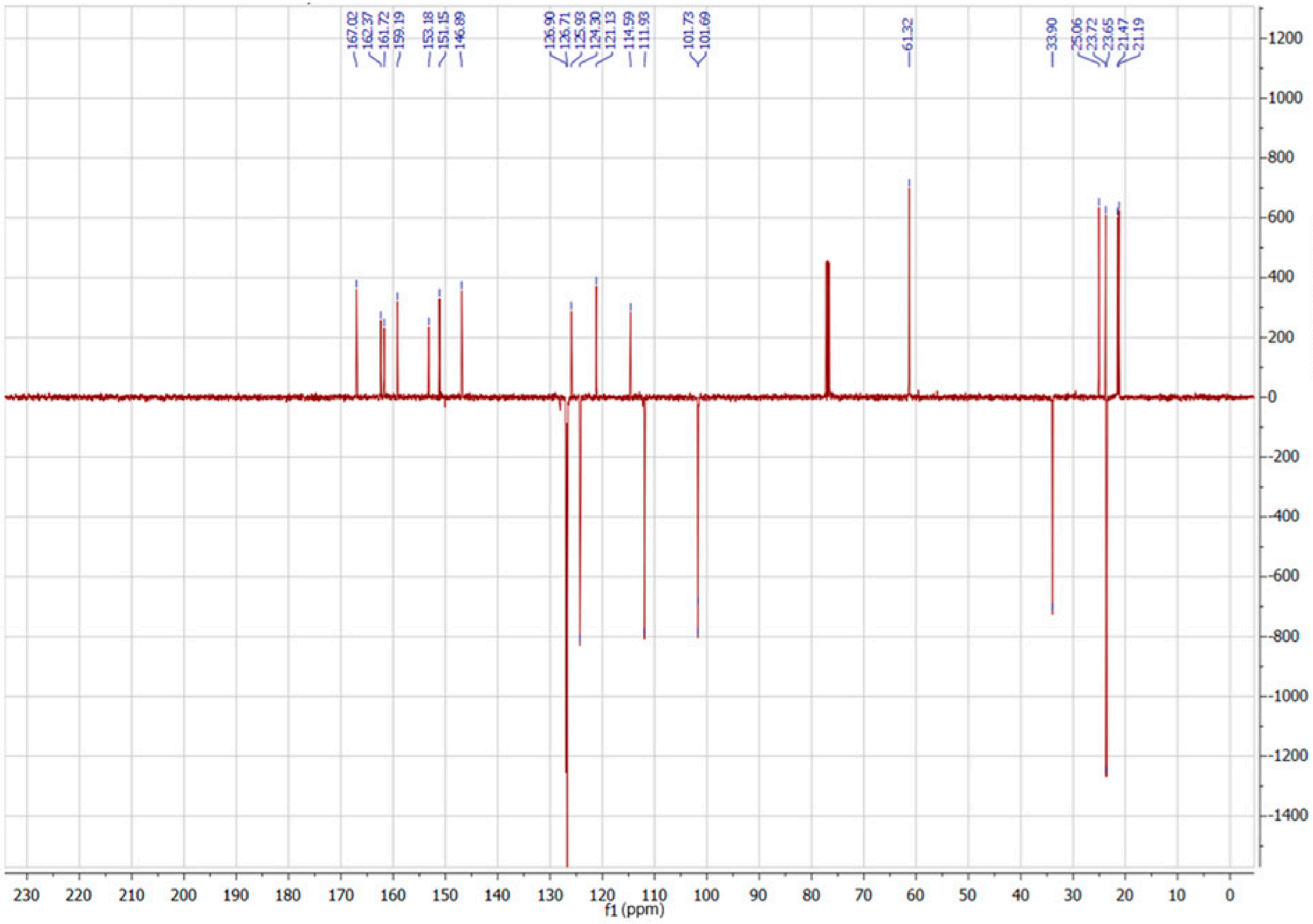

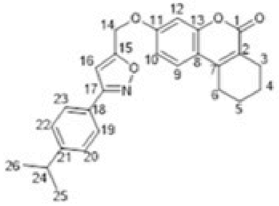

^{13}C NMR (101 MHz, $CDCl_3$) δ 167.02 (s, C-15), 162.37 (s, C-17), 161.72 (s, C-1), 159.19 (s, C-11), 153.18 (s, C-13), 151.15 (s, C-18), 146.89 (s, C-7), 126.90 (d, 2C, C-20,22), 126.71 (d, 2C, C-19,23), 125.93 (s, C-21), 124.30 (d, C-9), 121.13 (s, C-2), 114.59 (s, C-8), 111.93 (d, C-10), 101.73 (d, C-16), 101.69 (d, C-12), 61.32 (t, C-14), 33.90 (d, C-24), 25.06 (t, C-6), 23.72 (t, C-3), 23.65 (q, 2C, C-25,26), 21.47 (t, C-5), 21.19 (t, C-4).

Figure S15. ^{1}H spectra of Compound **15a**

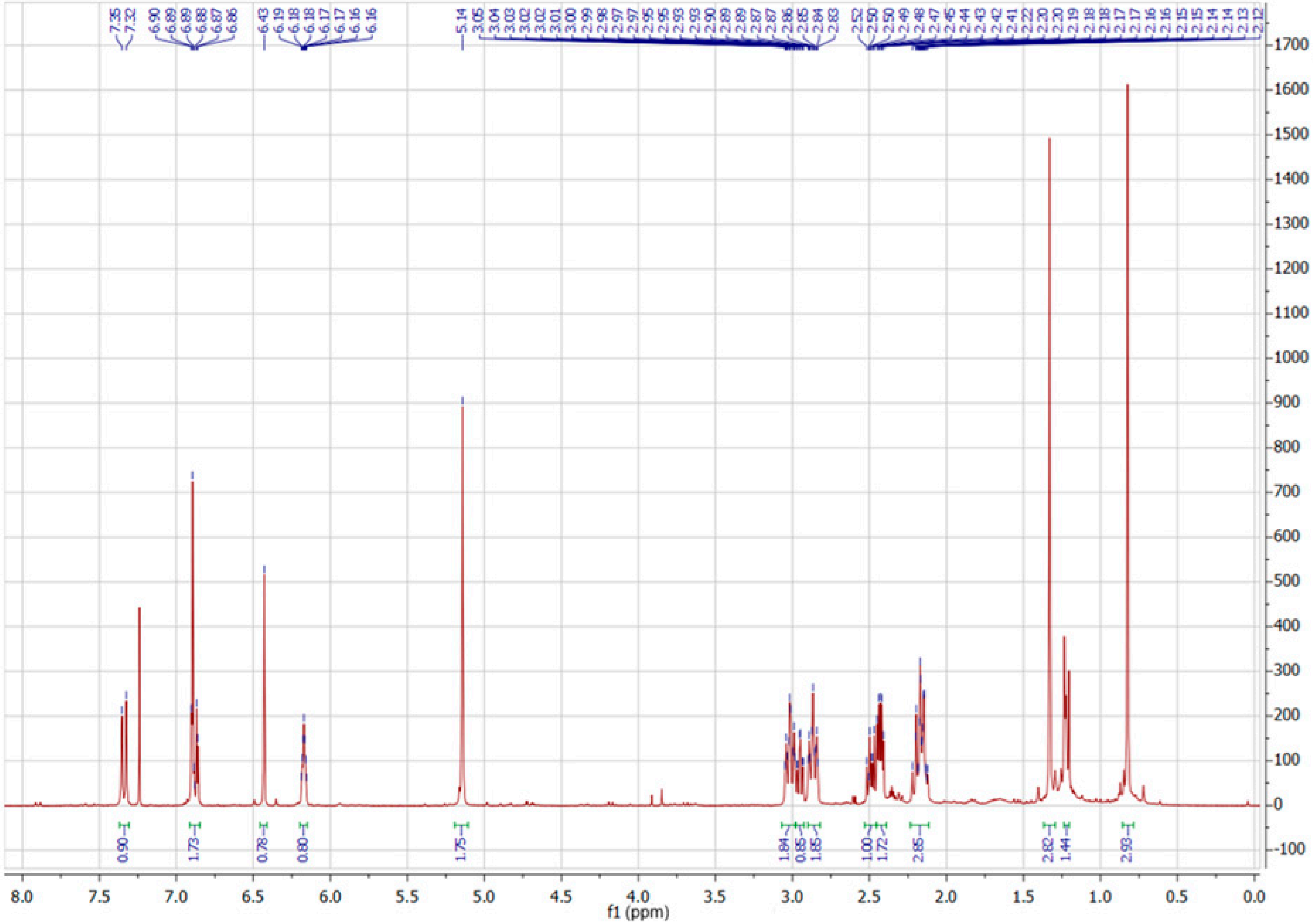

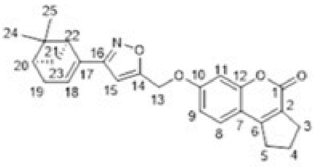

^{1}H NMR (300 MHz, $CDCl_3$) δ 7.34 (d, J = 8.3 Hz, 1H, H-8), 6.88 – 6.91 (m, 1H, H-11), 6.86 – 6.90 (m, 1H, H-9), 6.43 (s, 1H, H-15), 6.17 (tt, J = 3.2, 1.6 Hz, 1H, H-18), 5.14 (s, 2H, H-13), 3.02 (tt, J = 8.3, 1.9 Hz, 2H, H-5), 2.95 (td, J = 5.7, 1.6 Hz, 1H, H-22), 2.86 (tt, J = 7.5, 1.8 Hz, 2H, H-3), 2.49 (dt, J = 8.9, 5.7 Hz, 1H, H-23b), 2.39 – 2.47 (m, 2H, H-19), 2.11 – 2.21 (m, 3H, H-4, H-20), 1.33 (s, 3H, H-25), 1.19 – 1.26 (m, 1H, H-23a), 0.82 (s, 3H, H-24).

Figure S16. ^{13}C spectra of Compound **15a**

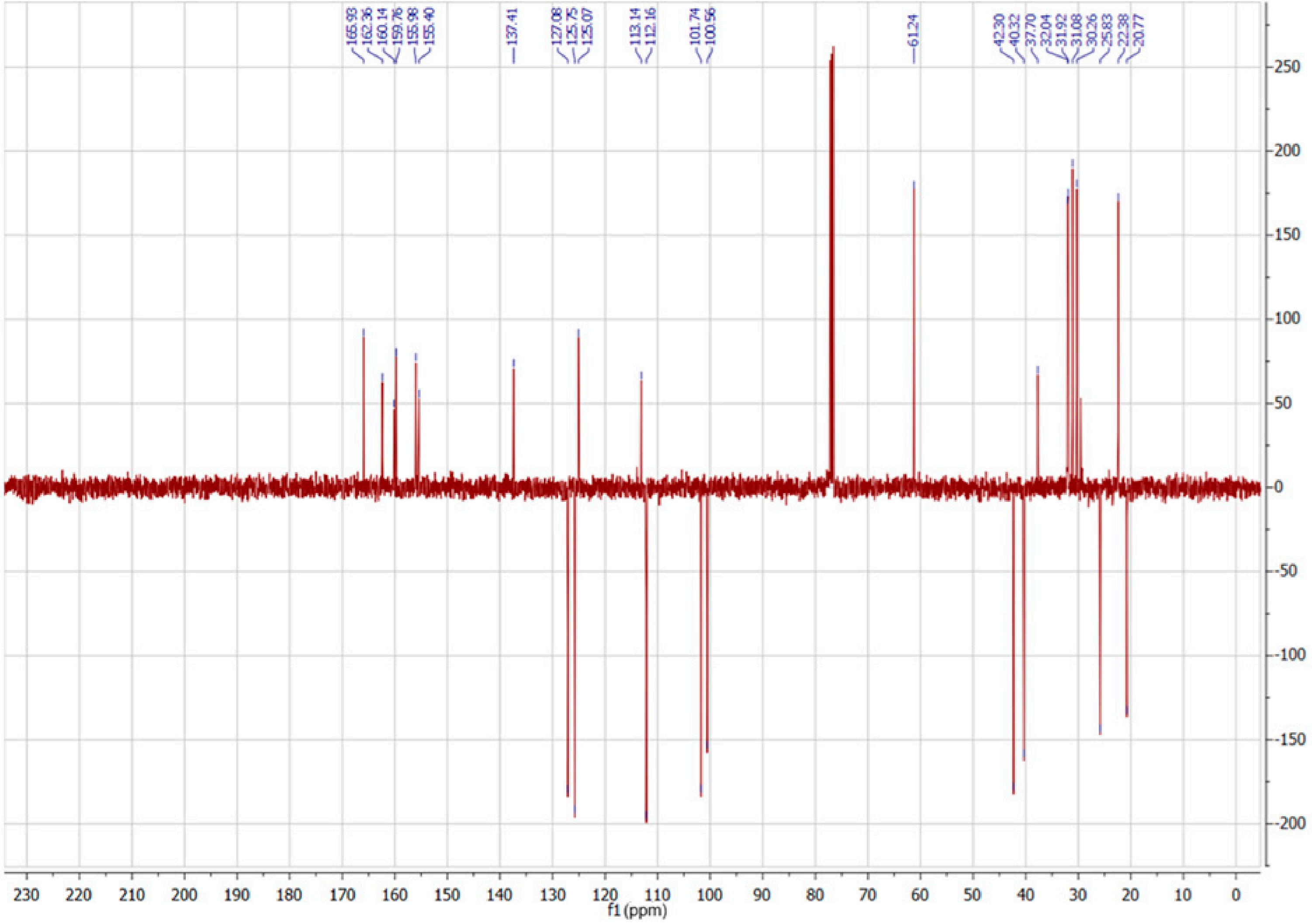

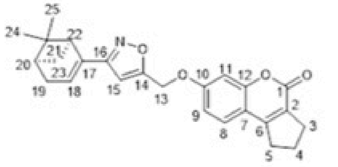

^{13}C NMR (75 MHz, $CDCl_3$) δ 165.93 (s, C-14), 162.36 (s, C-16), 160.14 (s, C-1), 159.76 (s, C-10), 155.98 (s, C-6), 155.40 (s, C-12), 137.41 (s, C-17), 127.09 (d, C-8), 125.75 (d, C-18), 125.07 (s, C-2), 113.14 (s, C-7), 112.16 (d, C-9), 101.74 (d, C-15), 100.56 (d, C-11), 61.24 (t, C-13), 42.30 (d, C-22), 40.32 (d, C-20), 37.70 (s, C-21), 32.04 (t, C-5), 31.92 (t, C-23), 31.08 (t, C-19), 30.26 (t, C-3), 25.83 (q, C-25), 22.38 (t, C-4), 20.77 (q, C-24).

Figure S17. ^{1}H spectra of Compound **15c**

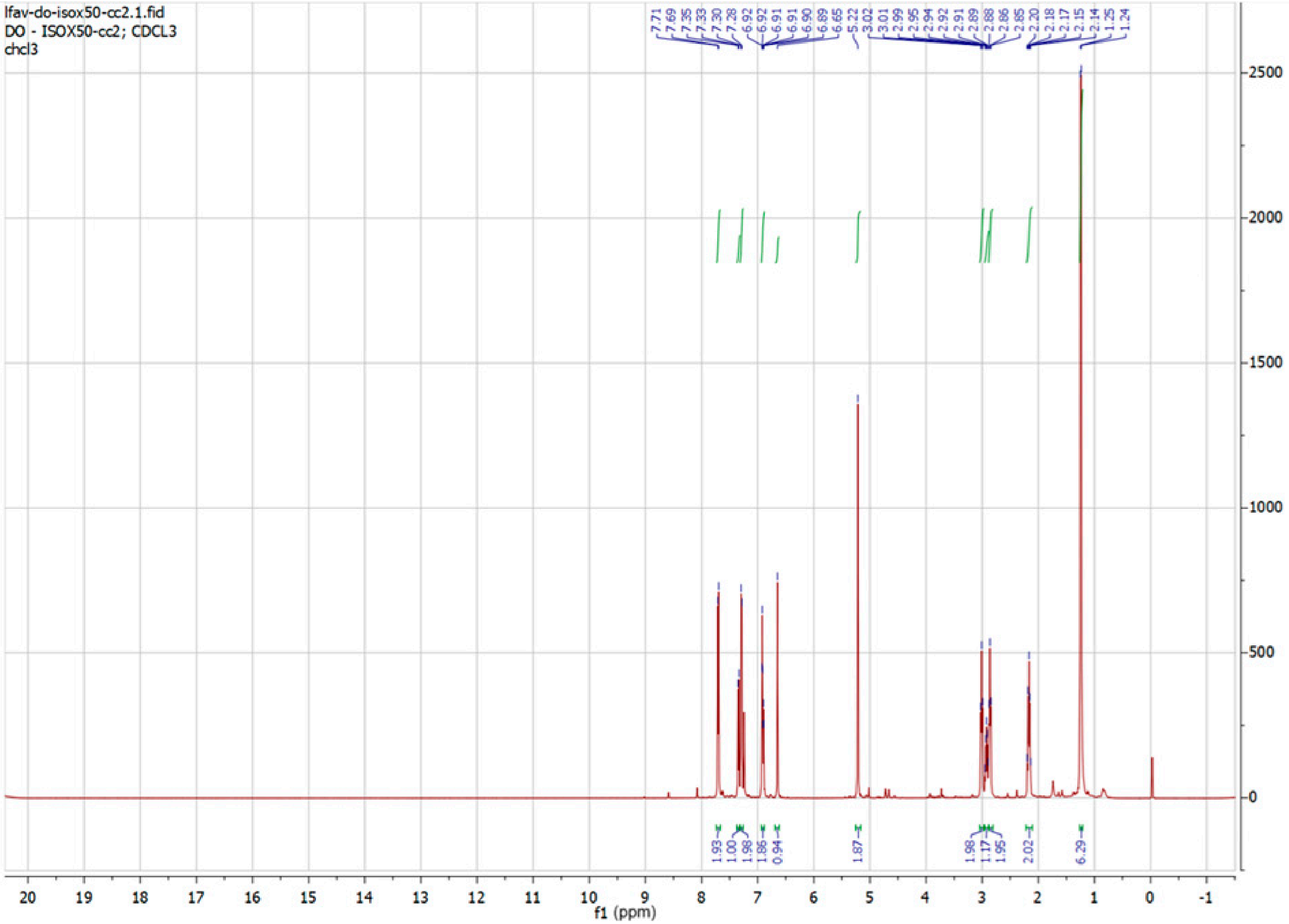

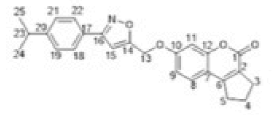

^{1}H NMR (500 MHz, $CDCl_3$) δ 7.70 (d, *J* = 7.9 Hz, 2H, H-18,22), 7.34 (d, *J* = 8.5 Hz, 1H, H-8), 7.29 (d, *J* = 7.9 Hz, 2H, H-19,21), 6.88 – 6.94 (m, 2H, H-9,11), 6.65 (s, 1H, H-15), 5.22 (s, 2H, H-13), 3.01 (t, *J* = 7.6 Hz, 2H, H-5), 2.92 (h, *J* = 6.9 Hz, 1H, H-23), 2.86 (t, *J* = 7.5 Hz, 2H, H-3), 2.17 (p, *J* = 7.6 Hz, 2H, H-4), 1.24 (d, *J* = 6.9 Hz, 6H, H-24,25).

Figure S18. ^{13}C spectra of Compound **15c**

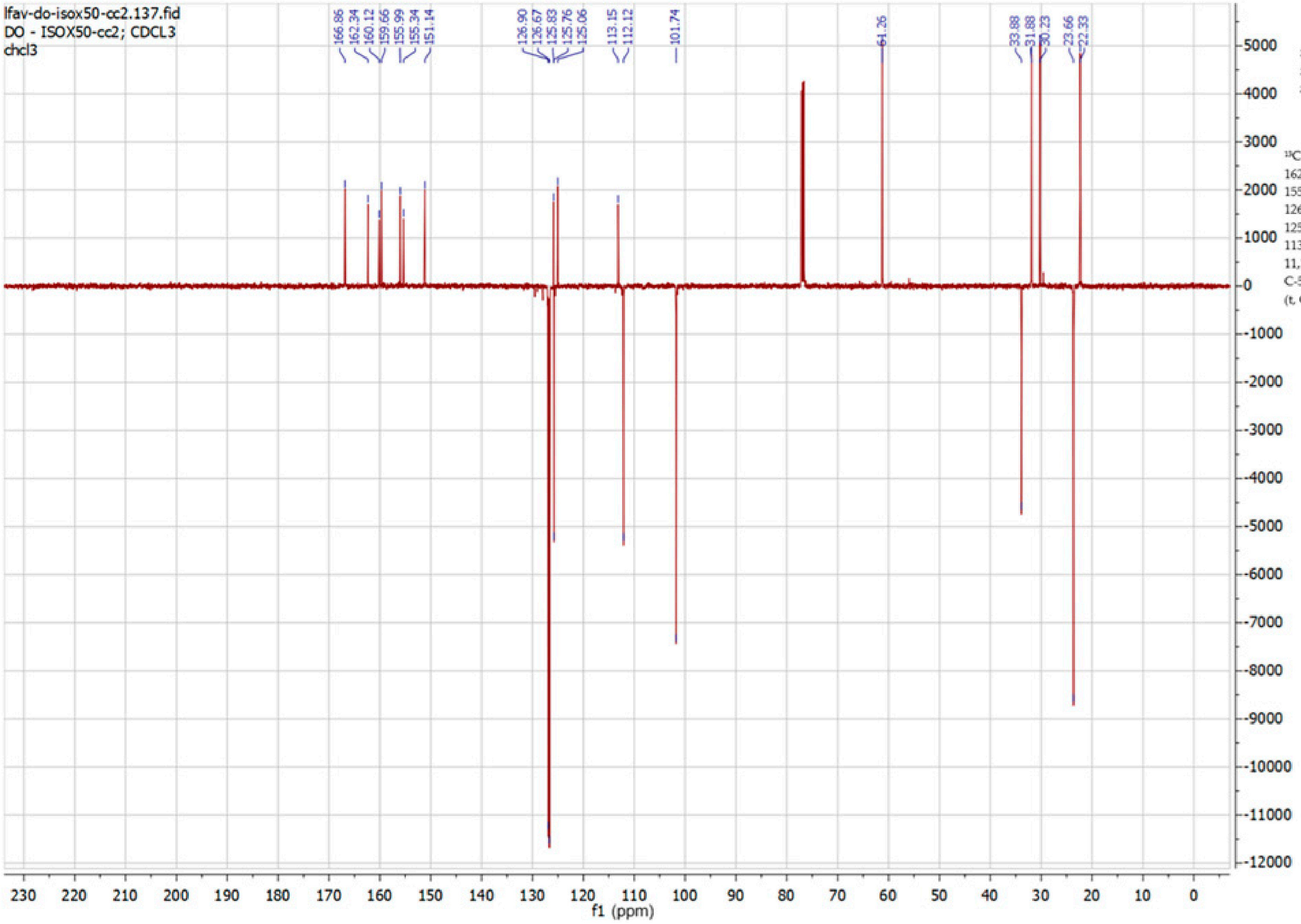

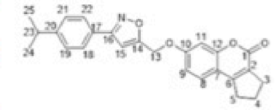

^{13}C NMR (126 MHz, $CDCl_3$) δ 166.86 (s, C-14), 162.34 (s, C-16), 160.12 (s, C-1), 159.66 (s, C-10), 155.99 (s, C-6), 155.34 (s, C-12), 151.14 (s, C-17), 126.90 (d, 2C, C-19,21), 126.67 (d, 2C, C-18,22), 125.83 (s, C-20), 125.76 (d, C-8), 125.06 (s, C-2), 113.15 (s, C-7), 112.12 (d, C-9), 101.74 (d, 2C, C-11,15), 61.26 (t, C-13), 33.88 (d, C-23), 31.88 (t, C-5), 30.23 (t, C-3), 23.66 (q, 2C, C-24,25), 22.33 (t, C-4).

Figure S19. ^{1}H spectra of Compound **16a**

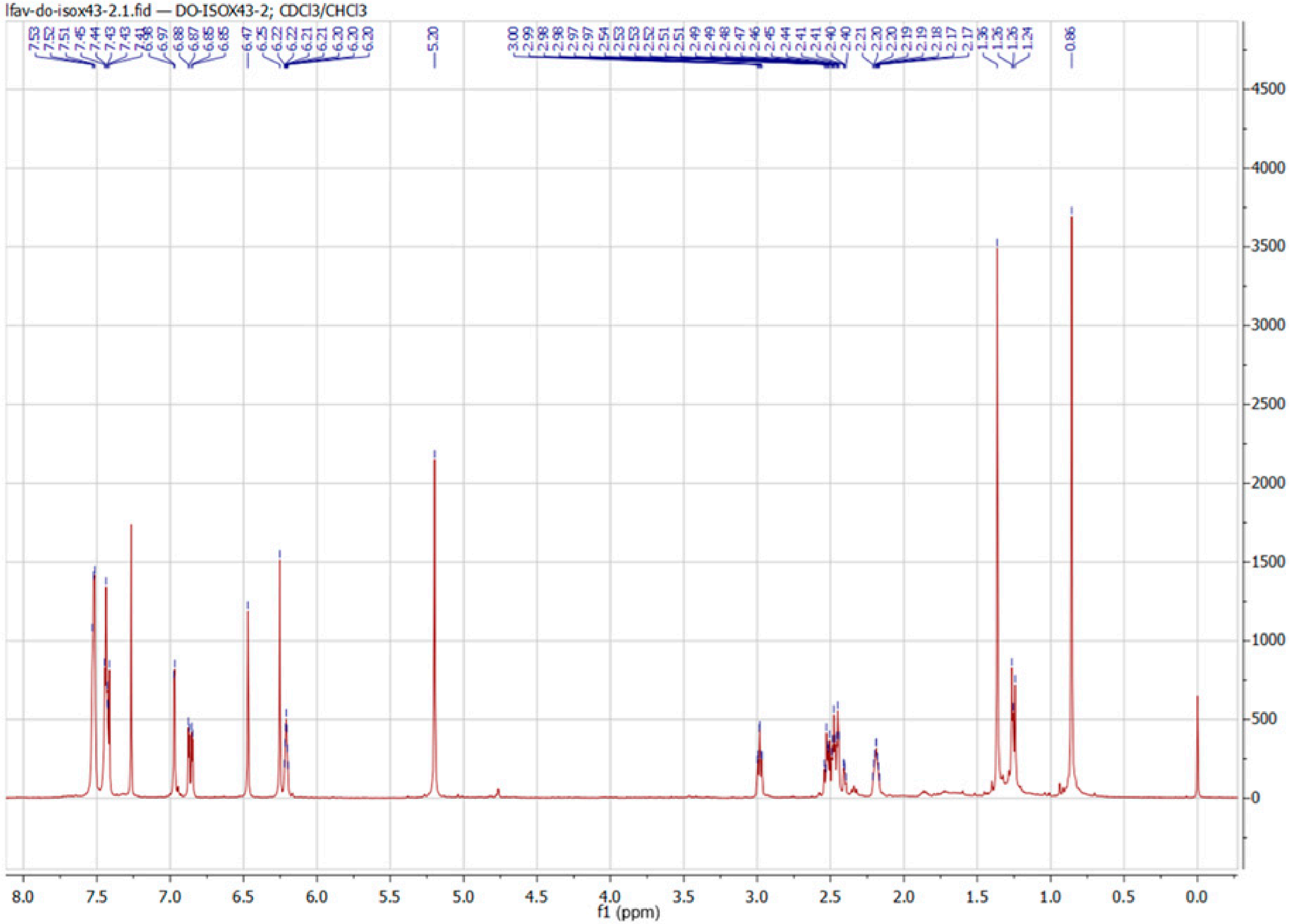

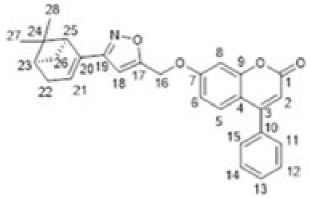

^{1}H NMR (400 MHz, $CDCl_3$) δ 7.48 – 7.56 (m, 3H, H-12,13,14), 7.39 – 7.46 (m, 3H, H-5,11,15), 6.97 (d, *J* = 2.6 Hz, 1H, H-8), 6.86 (dd, *J* = 8.9, 2.6 Hz, 1H, H-6), 6.47 (s, 1H, H-18), 6.25 (s, 1H, H-2), 6.21 (tt, *J* = 3.3, 1.5 Hz, 1H, H-21), 5.20 (s, 2H, H-16), 2.98 (td, *J* = 5.6, 1.6 Hz, 1H, H-25), 2.52 (td, *J* = 5.7, 3.4 Hz, 1H, H-26b), 2.39 – 2.49 (m, 2H, H-22), 2.15 – 2.22 (m, 1H, H-23), 1.36 (s, 3H, H-28), 1.24 – 1.27 (m, 1H, H-26a), 0.86 (s, 3H, H-27).

Figure S20. ^{13}C spectra of Compound **16a**

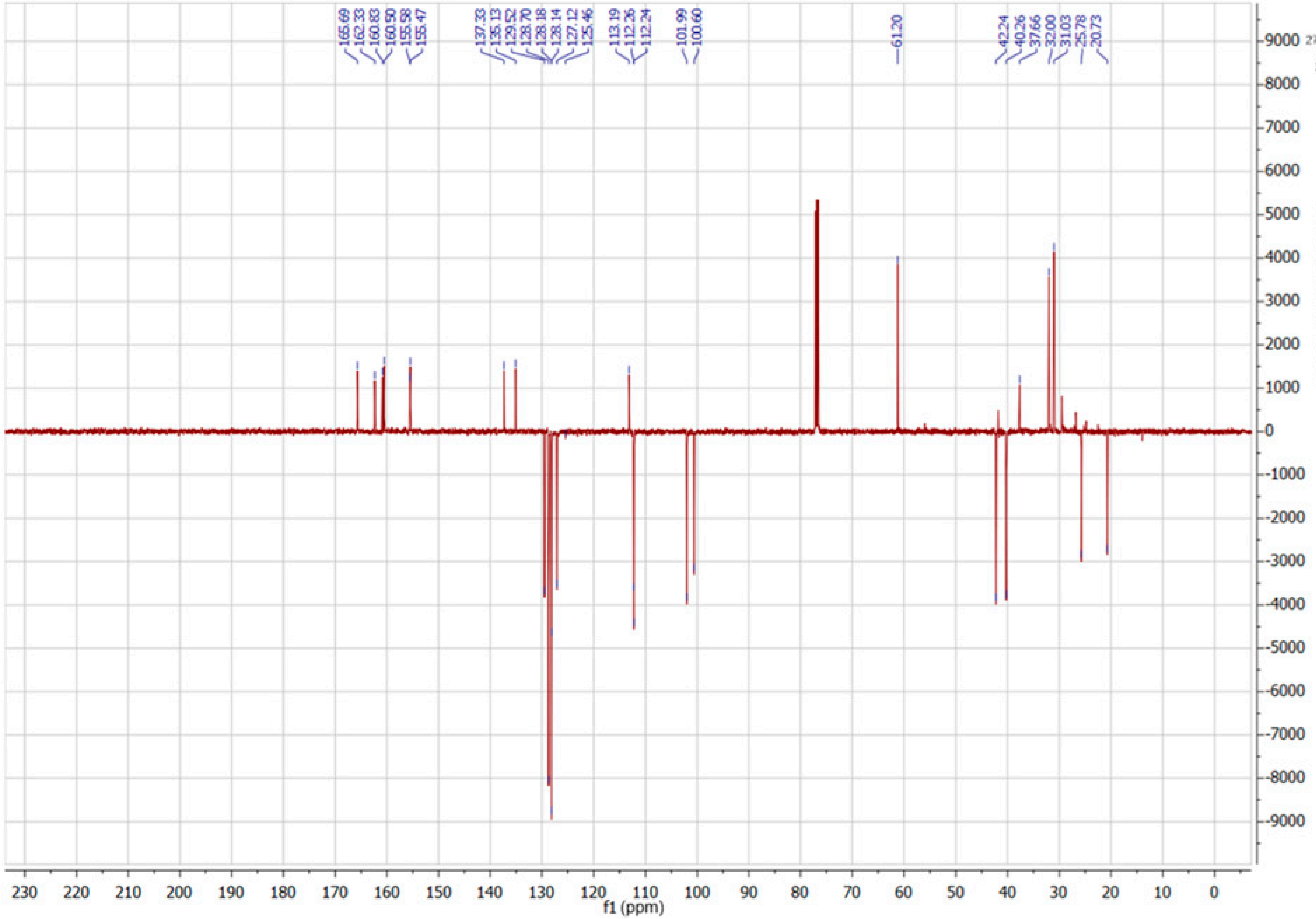

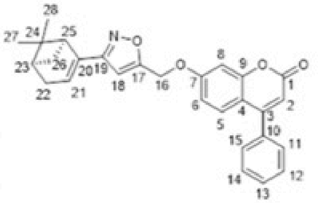

^{13}C NMR (126 MHz, $CDCl_3$) δ 165.69 (s, C-17), 162.33 (s, C-19), 160.83 (s, C-7), 160.50 (s, C-1), 155.58 (s, C-9), 155.47 (s, C-3), 137.33 (s, C-20), 135.13 (s, C-10), 129.52 (d, C-13), 128.70 (d, 2C, C-12,14), 128.18 (d, C-15), 128.14 (d, C-11), 127.12 (d, C-5), 125.46 (d, C-21), 113.19 (s, C-4), 112.26 (d, C-6), 112.24 (d, C-2), 101.99 (d, C-18), 100.60 (d, C-8), 61.20 (t, C-16), 42.24 (d, C-25), 40.26 (d, C-23), 37.66 (s, C-24), 32.00 (t, C-26), 31.03 (t, C-22), 25.78 (q, C-28), 20.73 (q, C-27).

Figure S21. ^{1}H spectra of Compound **16c**

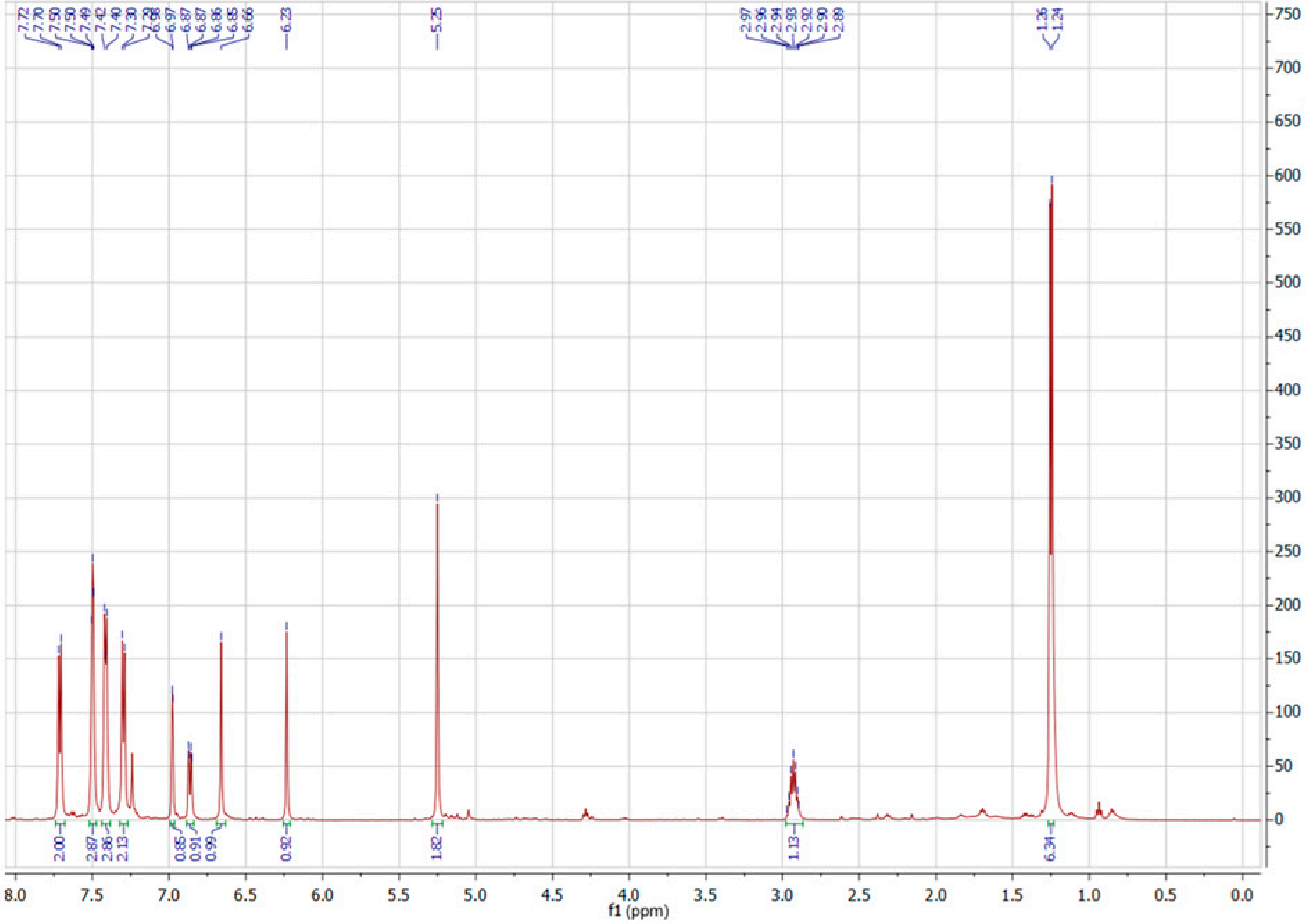

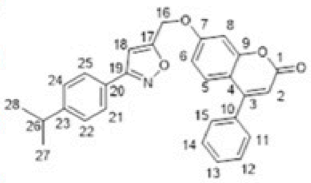

^{1}H NMR (500 MHz, $CDCl_3$) δ 7.71 (d, *J* = 7.9 Hz, 2H, H-21,25), 7.47 – 7.53 (m, 3H, H-12,13,14), 7.39 – 7.44 (m, 3H, H-5,11,15), 7.30 (d, *J* = 7.9 Hz, 2H, H-22,24), 6.98 (d, *J* = 2.5 Hz, 1H, H-8), 6.86 (dd, *J* = 9.0, 2.5 Hz, 1H, H-6), 6.66 (s, 1H, H-18), 6.23 (s, 1H, H-2), 5.25 (s, 2H, H-16), 2.93 (h, *J* = 6.7 Hz, 1H, H-26), 1.25 (d, *J* = 6.7 Hz, 6H, H-27,28).

Figure S22. ^{13}C spectra of Compound **16c**

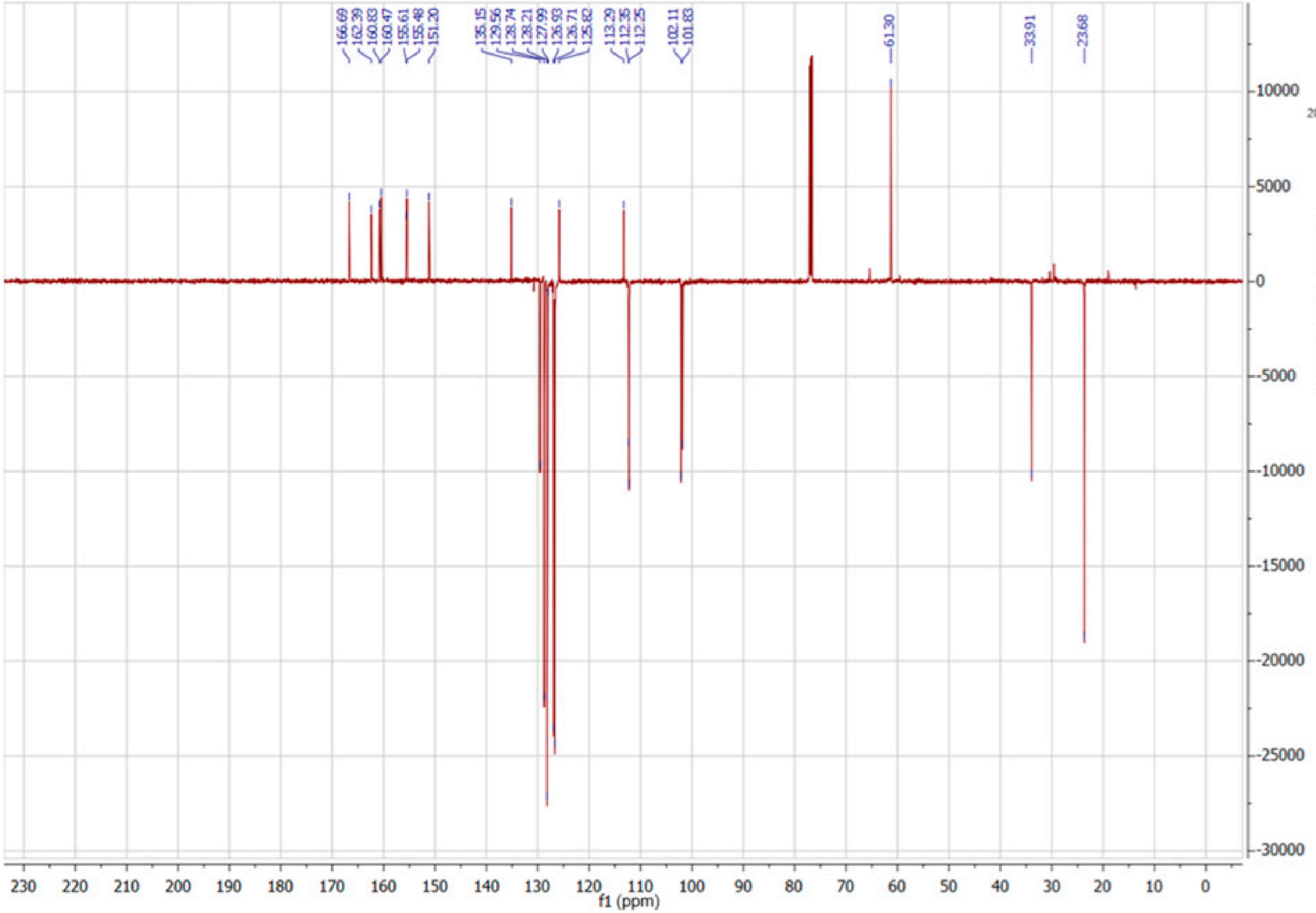

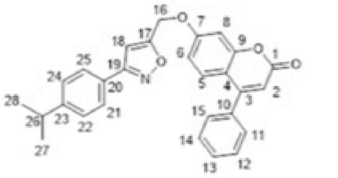

^{13}C NMR (126 MHz, $CDCl_3$) δ 166.69 (s, C-17), 162.39 (s, C-19), 160.83 (s, C-7), 160.47 (s, C-1), 155.61 (s, C-9), 155.48 (s, C-3), 151.20 (s, C-23), 135.15 (s, C-10), 129.56 (d, C-13), 128.74 (d, 2C, C-12,14), 128.21 (d, 2C, C-11,15), 127.99 (d, C-5), 126.93 (d, 2C, C-22,24), 126.71 (d, 2C, C-21,25), 125.82 (s, C-20), 113.29 (d, C-2), 112.35 (d, C-6), 112.25 (s, C-4), 102.11 (d, C-18), 101.83 (d, C-8), 61.30 (t, C-16), 33.91 (d, C-26), 23.68 (q, 2C, C-27,28).

Figure S23. ^{1}H spectra of Compound **17a**

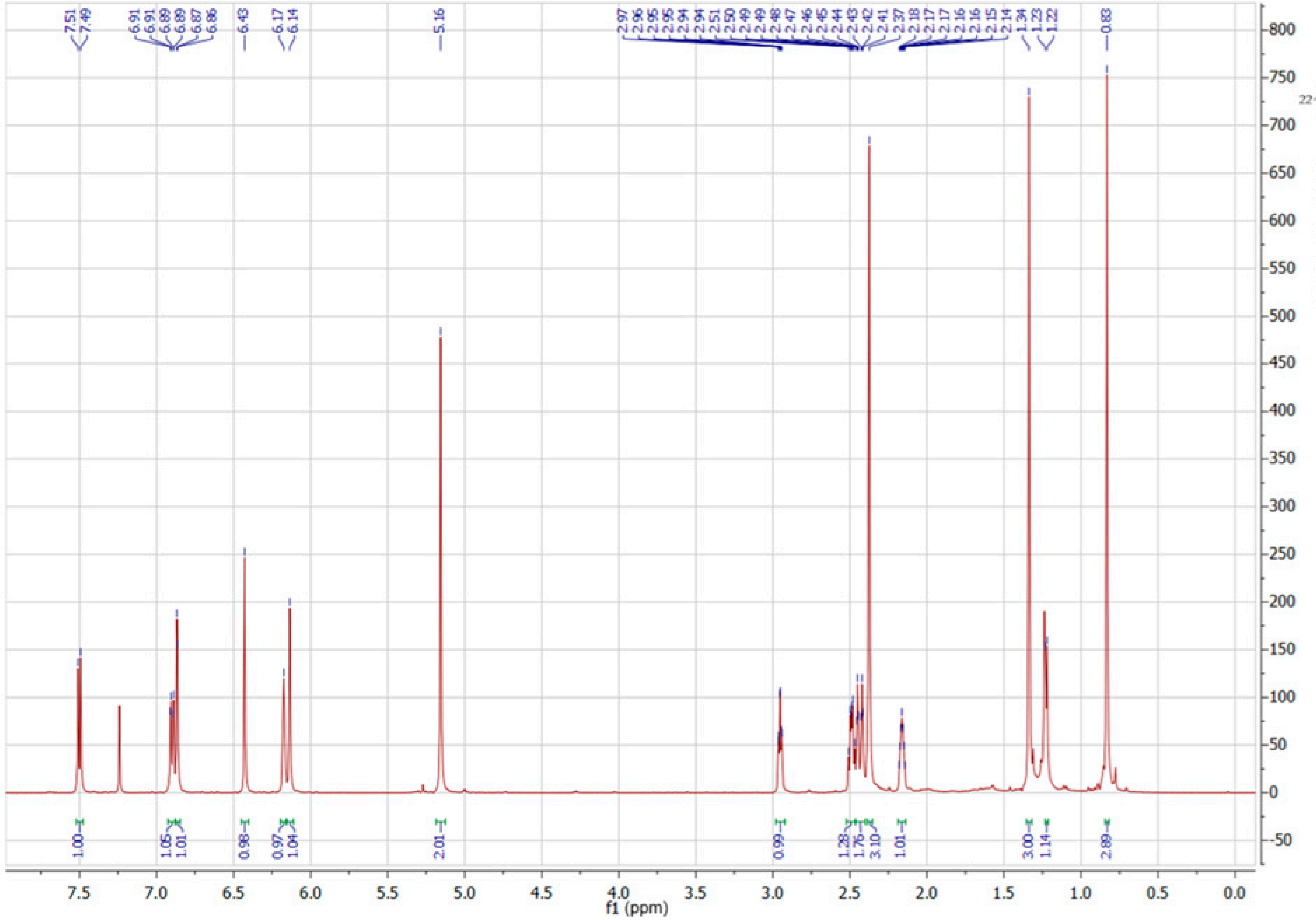

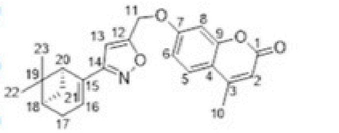

^{1}H NMR (400 MHz, $CDCl_3$) δ 7.50 (d, J = 8.8 Hz, 1H, H-5), 6.90 (dd, J = 8.8, 2.6 Hz, 1H, H-6), 6.87 (d, J = 2.6 Hz, 1H, H-8), 6.43 (s, 1H, H-13), 6.18 (s, 1H, H-2), 6.14 (s, 1H, H-16), 5.16 (s, 2H, H-11), 2.95 (td, J = 5.7, 1.5 Hz, 1H, H-20), 2.49 (dt, J = 9.0, 5.7 Hz, 1H, H-21b), 2.37 – 2.49 (m, 2H, H-17), 2.37 (s, 3H, H-10), 2.16 (tt, J = 3.4, 2.8 Hz, 1H, H-18), 1.34 (s, 3H, H-23), 1.23 (d, J = 9.0 Hz, 1H, H-21a), 0.83 (s, 3H, H-22).

Figure S24. ^{13}C spectra of Compound **17a**

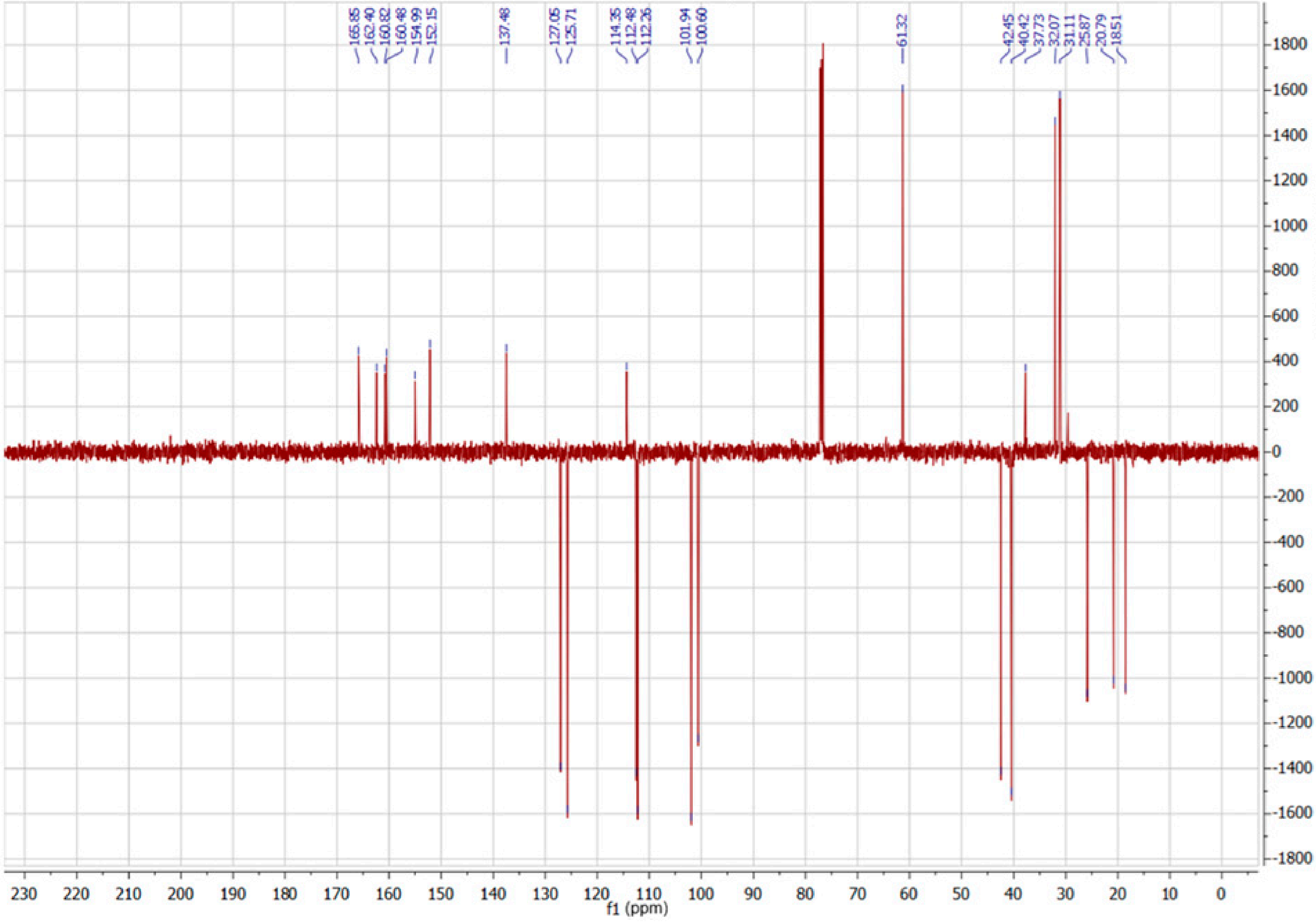

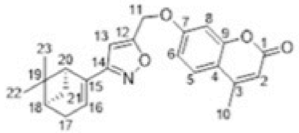

^{13}C NMR (101 MHz, $CDCl_3$) δ 165.85 (s, C-12), 162.40 (s, C-14), 160.82 (s, C-7), 160.48 (s, C-1), 154.99 (s, C-9), 152.15 (s, C-3), 137.48 (s, C-15), 127.05 (d, C-5), 125.71 (d, C-16), 114.35 (s, C-4), 112.47 (d, C-6), 112.25 (d, C-2), 101.94 (d, C-13), 100.60 (d, C-8), 61.32 (t, C-11), 42.45 (d, C-20), 40.42 (d, C-18), 37.73 (s, C-19), 32.07 (t, C-21), 31.11 (t, C-17), 25.87 (q, C-23), 20.79 (q, C-22), 18.51 (q, C-10).

Figure S25. ^{1}H spectra of Compound **17c**

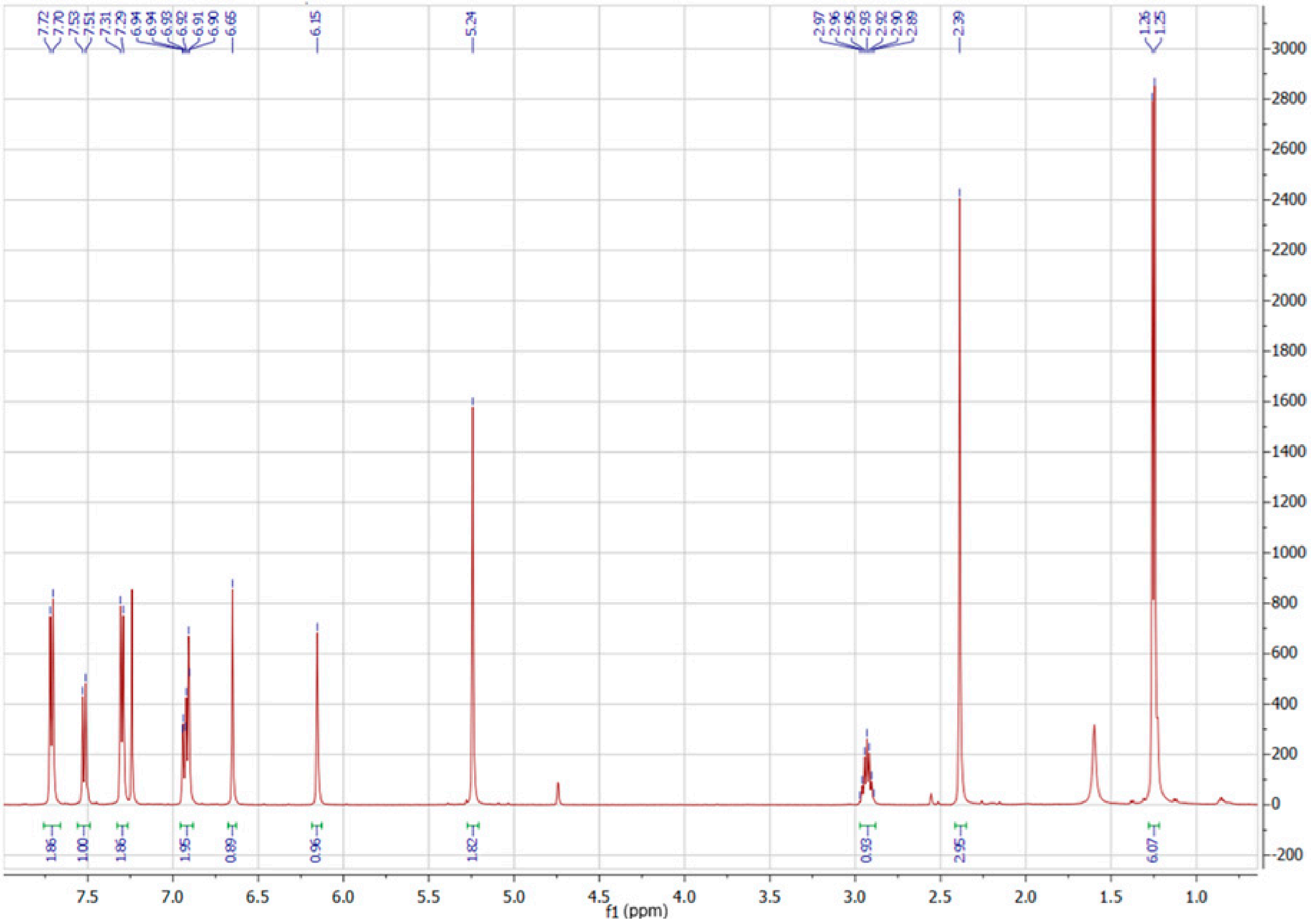

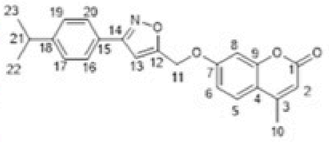

^{1}H NMR (400 MHz, $CDCl_3$) δ 7.71 (d, *J* = 8.1 Hz, 2H, H-16,20), 7.52 (d, *J* = 8.7 Hz, 1H, H-5), 7.30 (d, *J* = 8.0 Hz, 2H, H-17,19), 6.88 – 6.96 (m, 2H, H-6, H-8), 6.65 (s, 1H, H-13), 6.15 (s, 1H, H-2), 5.24 (s, 2H, H-11), 2.92 (h, *J* = 6.9 Hz, 1H, H-21), 2.39 (s, 3H, H-10), 1.25 (d, *J* = 6.9 Hz, 6H, H-22,23).

Figure S26. ^{13}C spectra of Compound **17c**

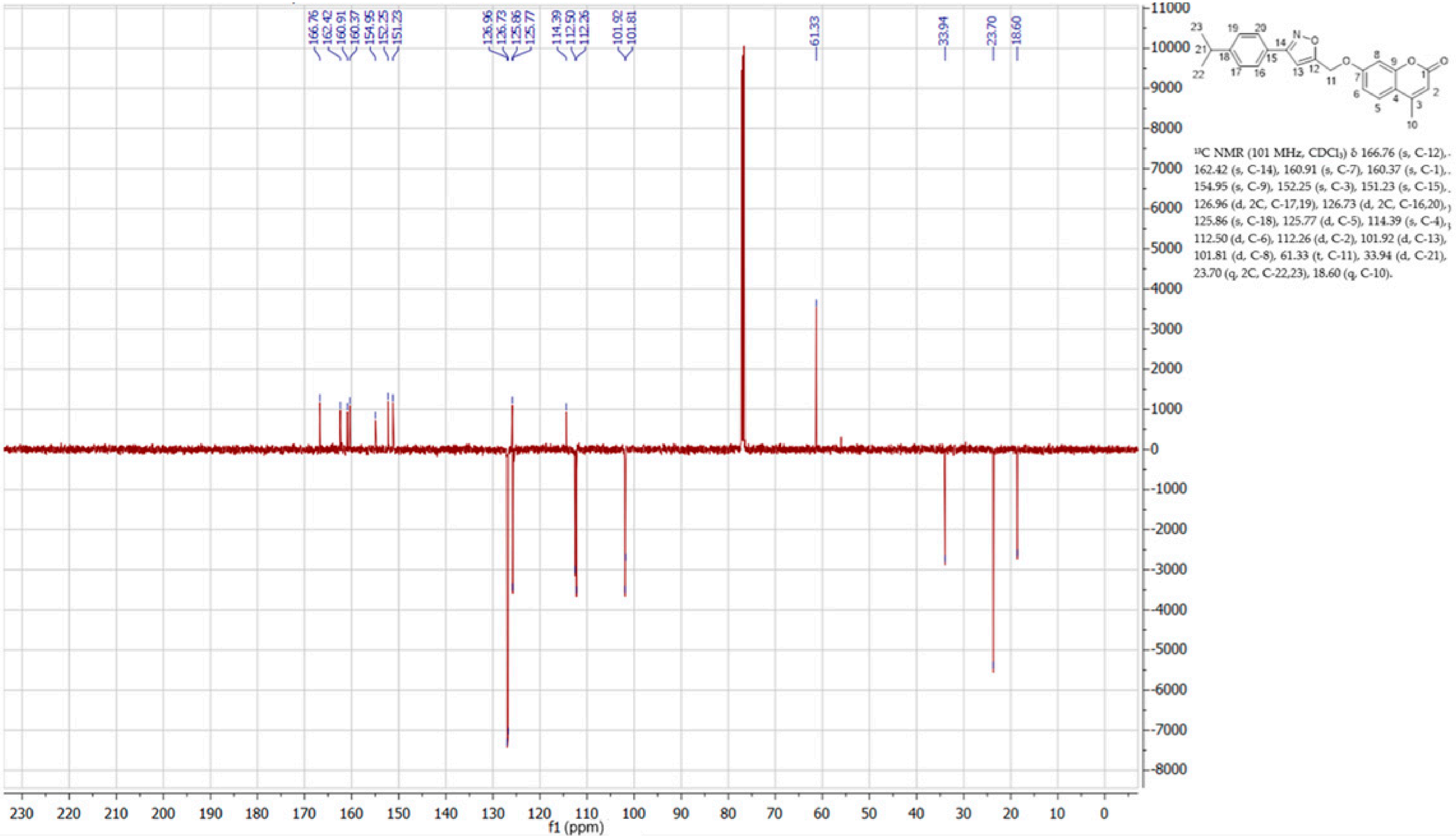

Figure S27. ^{1}H spectra of Compound **13e**

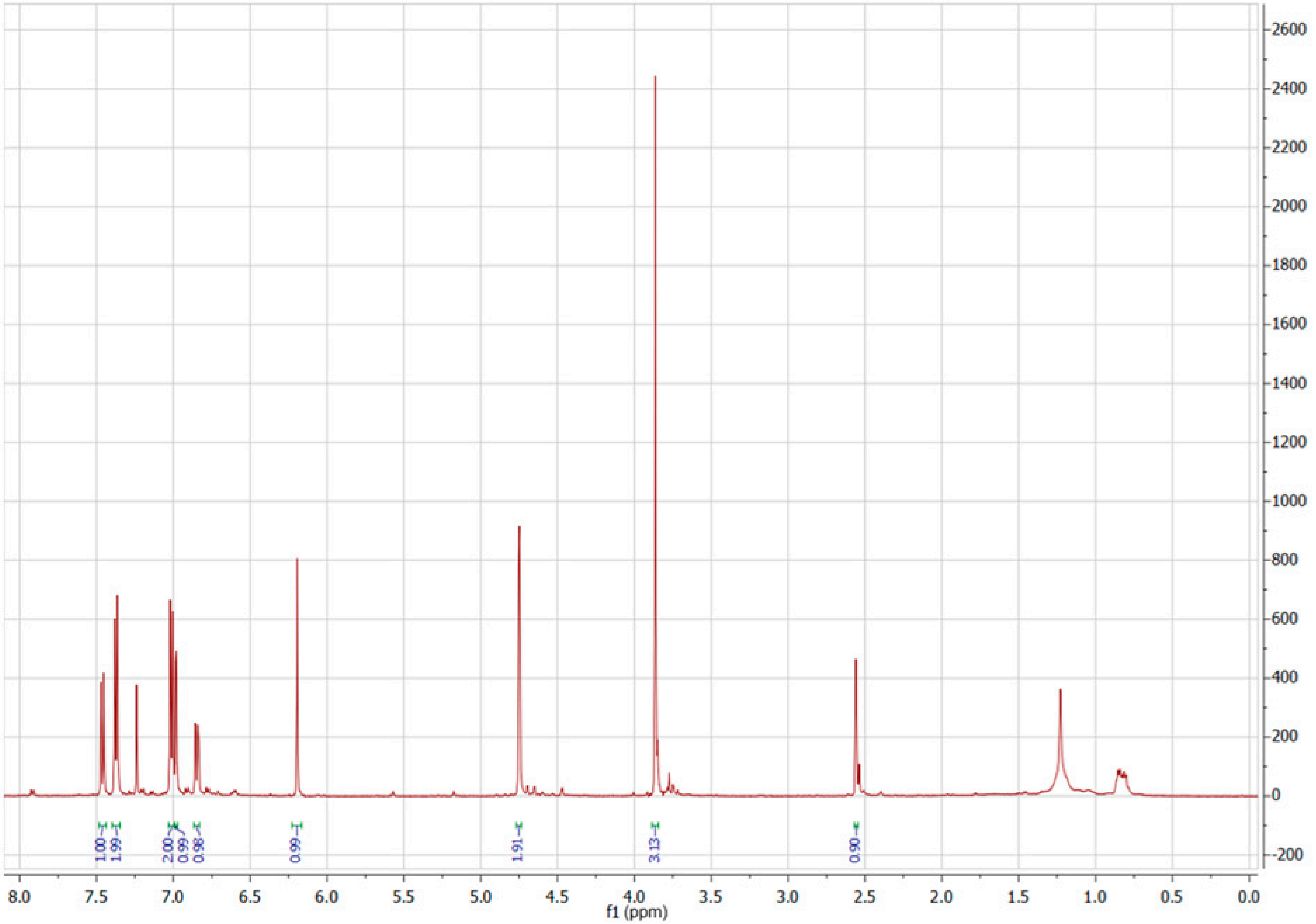

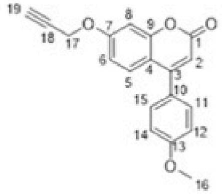

^{1}H NMR (400 MHz, $CDCl_3$) δ 7.46 (d, *J* = 8.9 Hz, 1H, H-5), 7.37 (d, *J* = 8.5 Hz, 2H, H-11,15), 7.01 (d, *J* = 8.5 Hz, 2H, H-12,14), 6.98 (d, *J* = 2.5 Hz, 1H, H-8), 6.85 (dd, *J* = 8.9, 2.5 Hz, 1H, H-6), 6.19 (s, 1H, H-2), 4.75 (d, *J* = 2.5 Hz, 2H, H-17), 3.86 (s, 3H, H-16), 2.56 (t, *J* = 2.5 Hz, 1H, H-19).

Figure S28. ^{13}C spectra of Compound **13e**

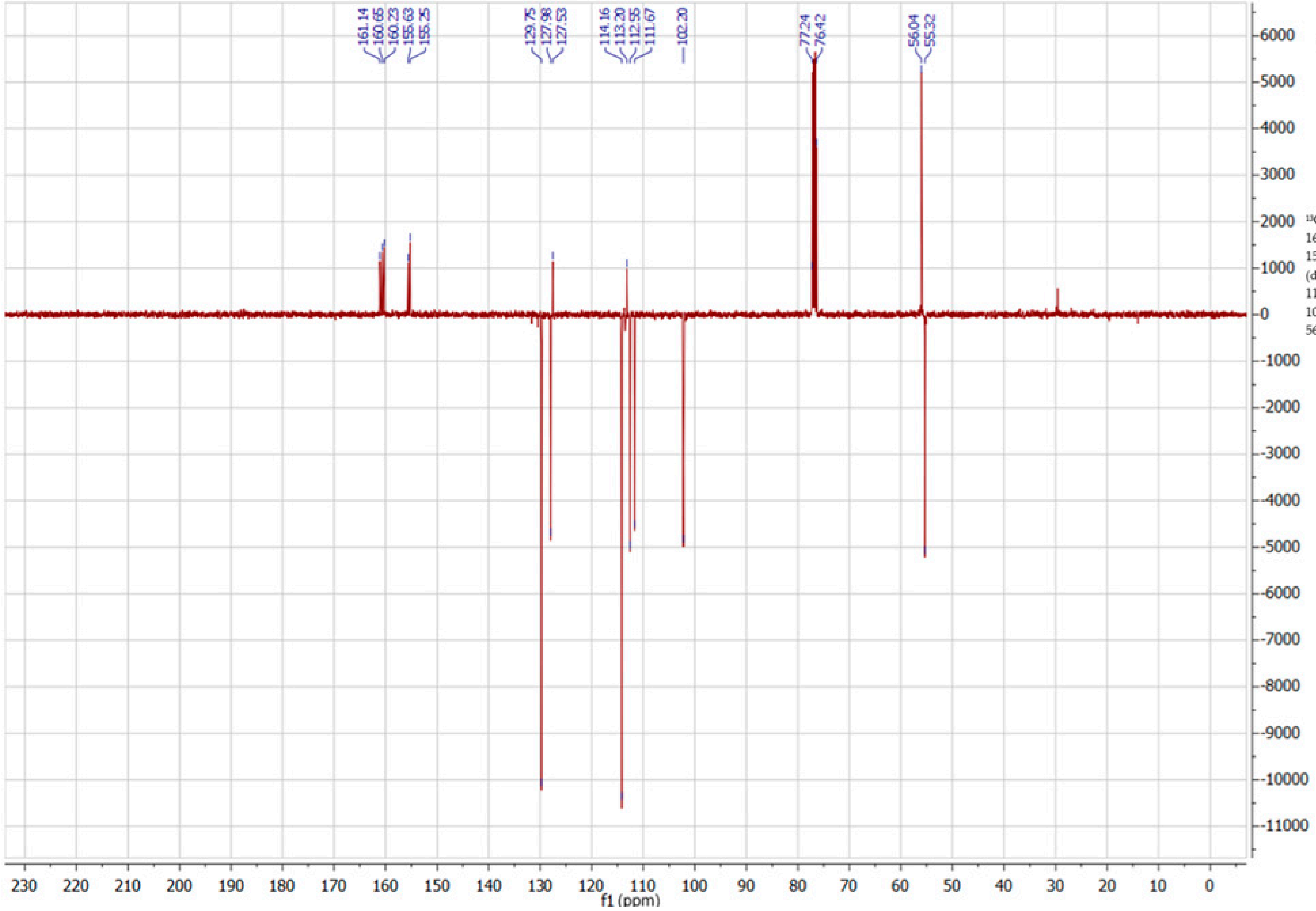

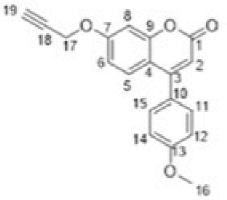

^{13}C NMR (101 MHz, $CDCl_3$) δ 161.14 (s, C-7), 160.65 (s, C-1), 160.23 (s, C-9), 155.63 (s, C-3), 155.25 (s, C-10), 129.75 (d, 2C, C-11,15), 127.98 (d, 2C, C-12,14), 127.53 (s, C-13), 114.16 (d, C-5), 113.20 (s, C-4), 112.55 (d, C-6), 111.67 (d, C-2), 102.20 (d, C-8), 77.24 (d, C-19), 76.42 (s, C-18), 56.04 (t, C-17), 55.32 (q, C-16).

2. HRMS spectra of the compounds 26a-26e; 14a-c, 15a-c, 16a-c, 17a-c, 13e.

Figure S29. HRMS spectra of Compound **26a**

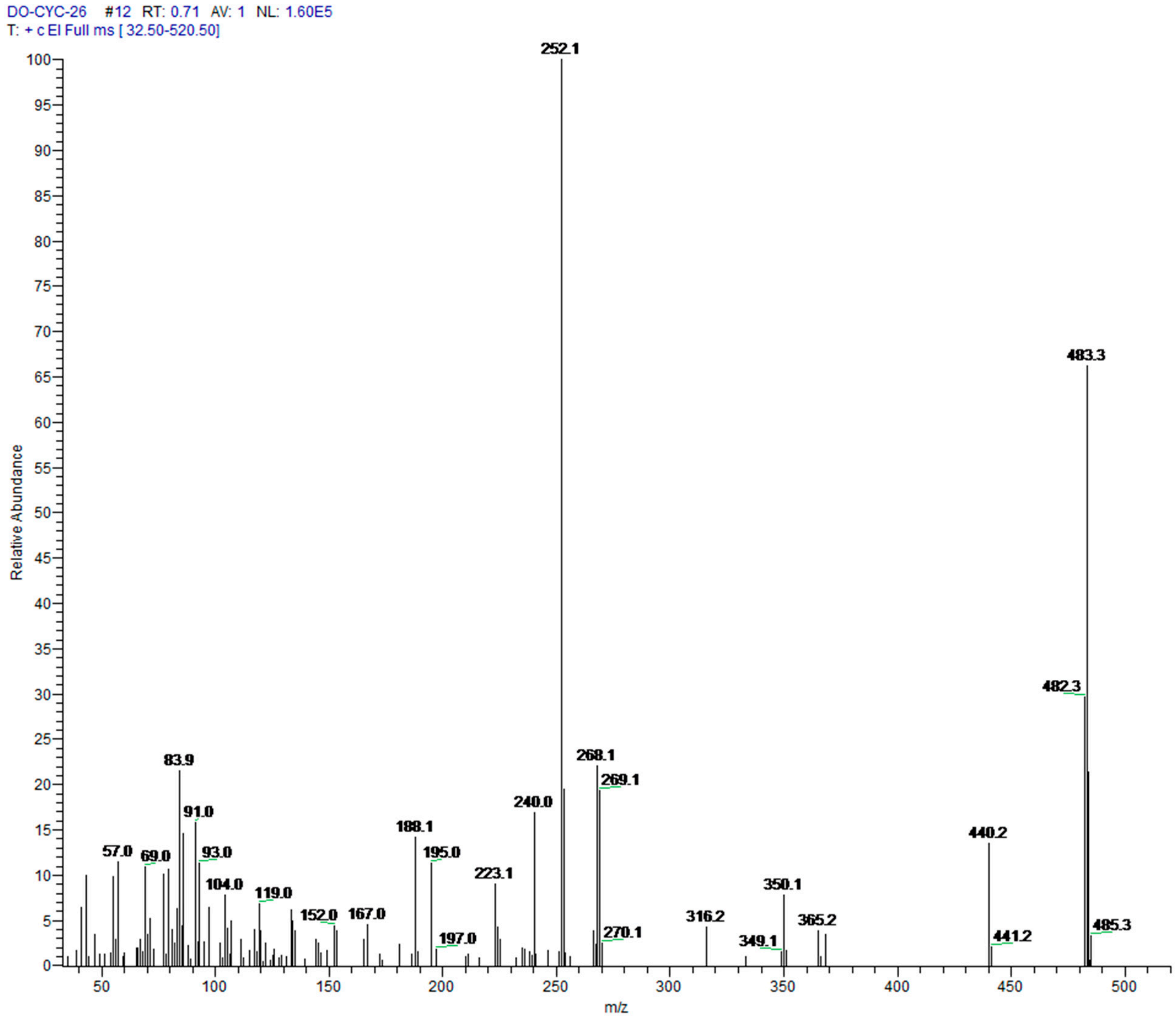

Figure S30. HRMS spectra of Compound **26b**

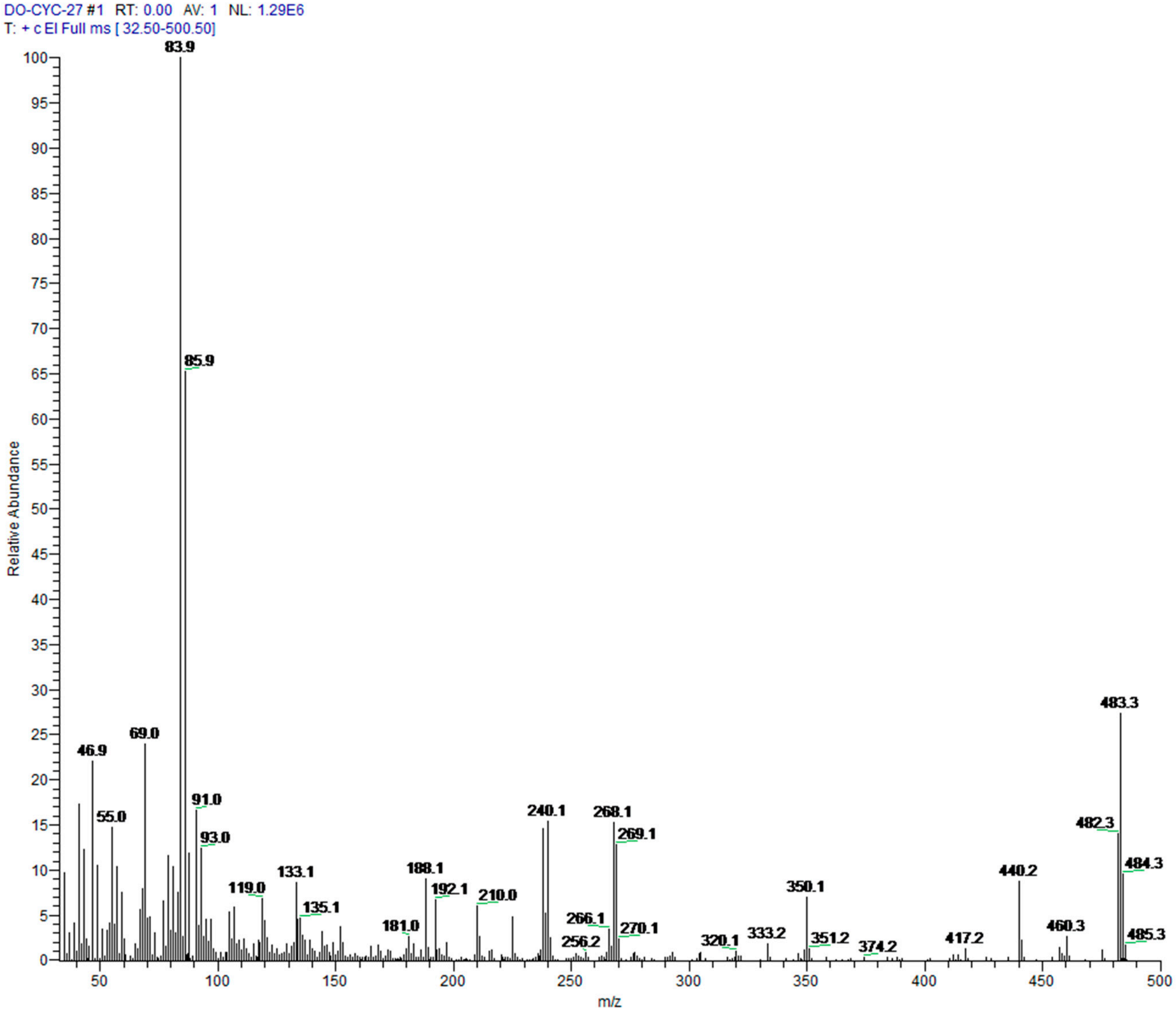

Figure S31. HRMS spectra of Compound **26c**

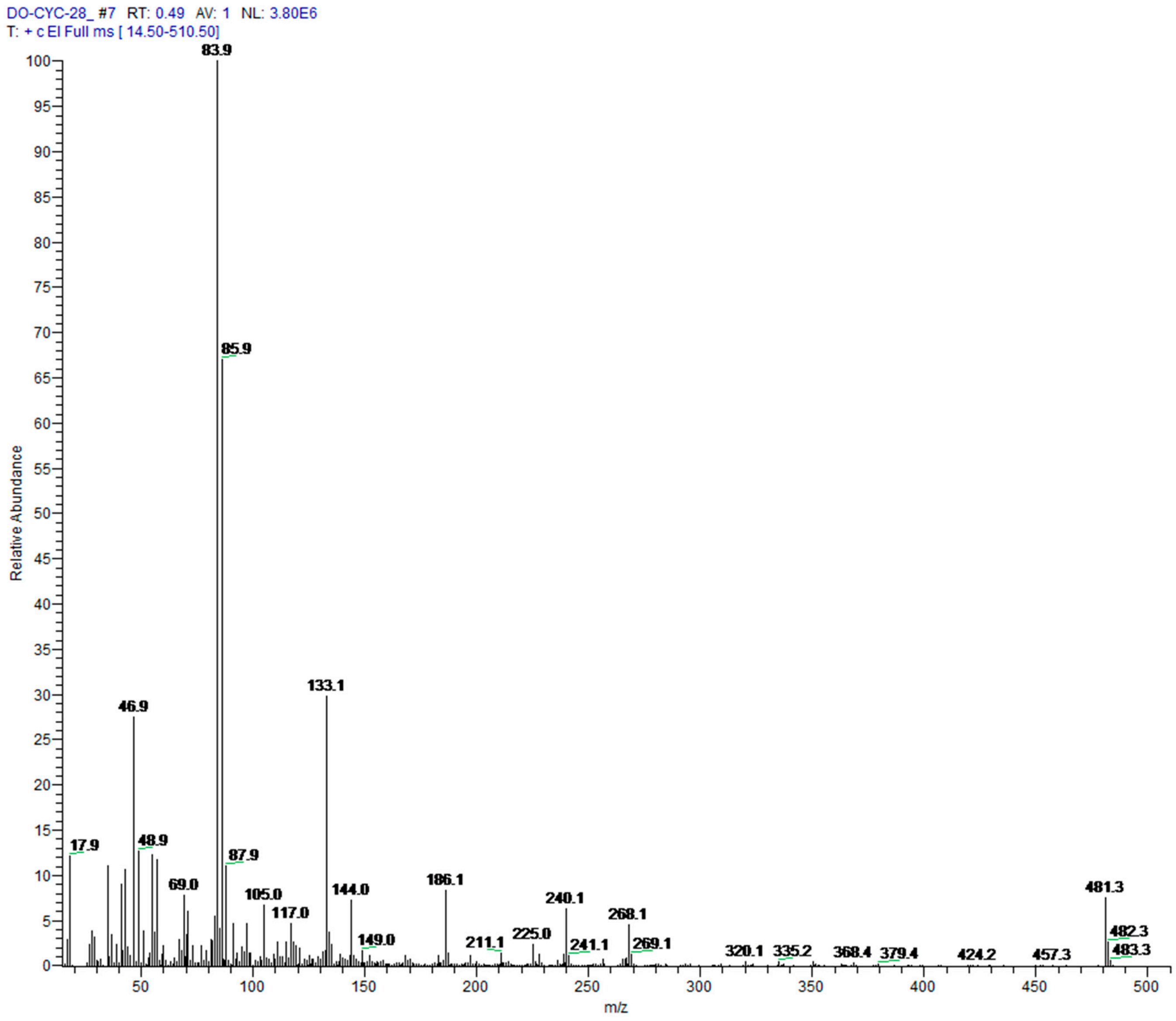

Figure S32. HRMS spectra of Compound **26d-*E***

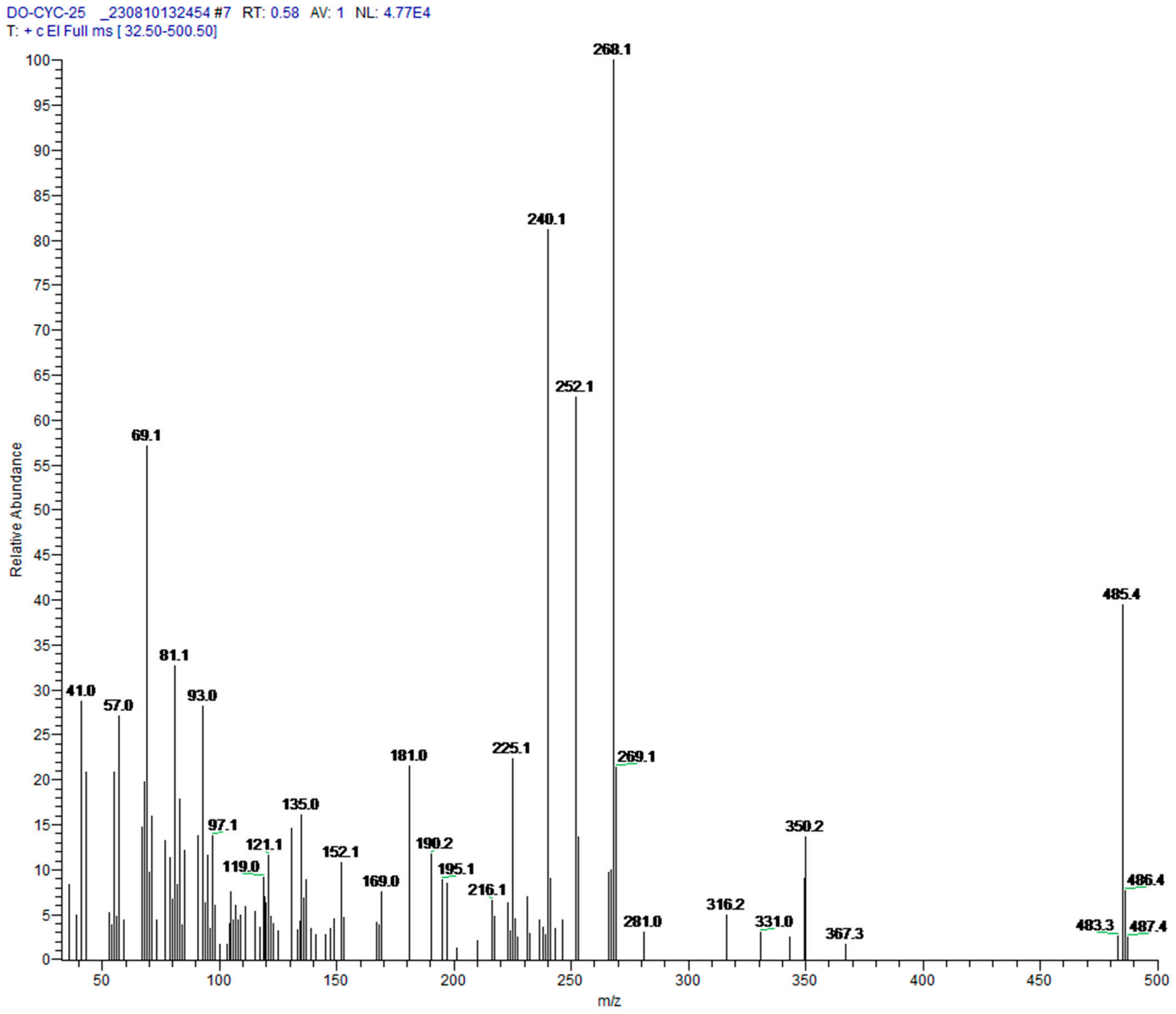

Figure S33. HRMS spectra of Compound **26e**

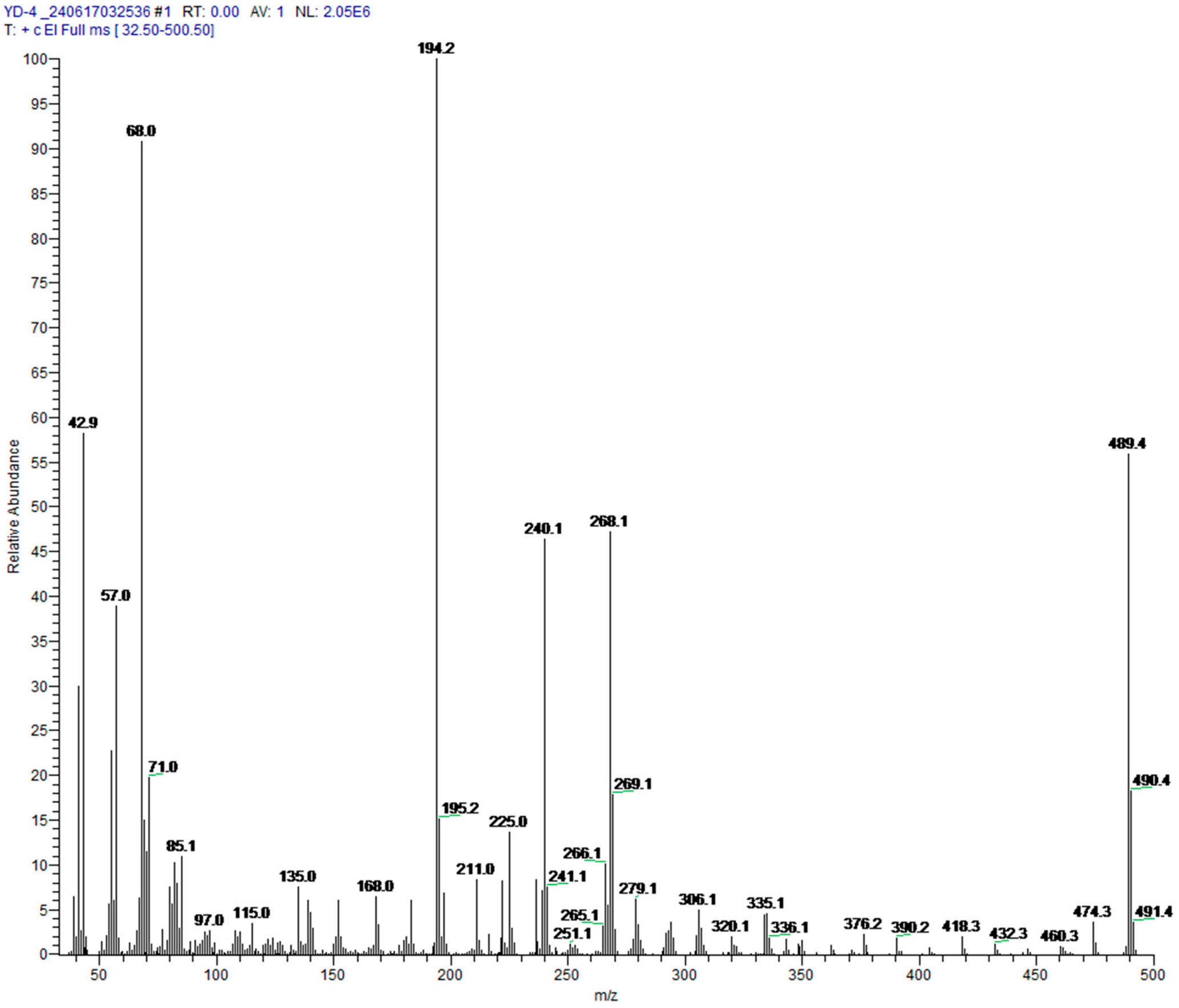

Figure S34. HRMS spectra of Compound **14a**

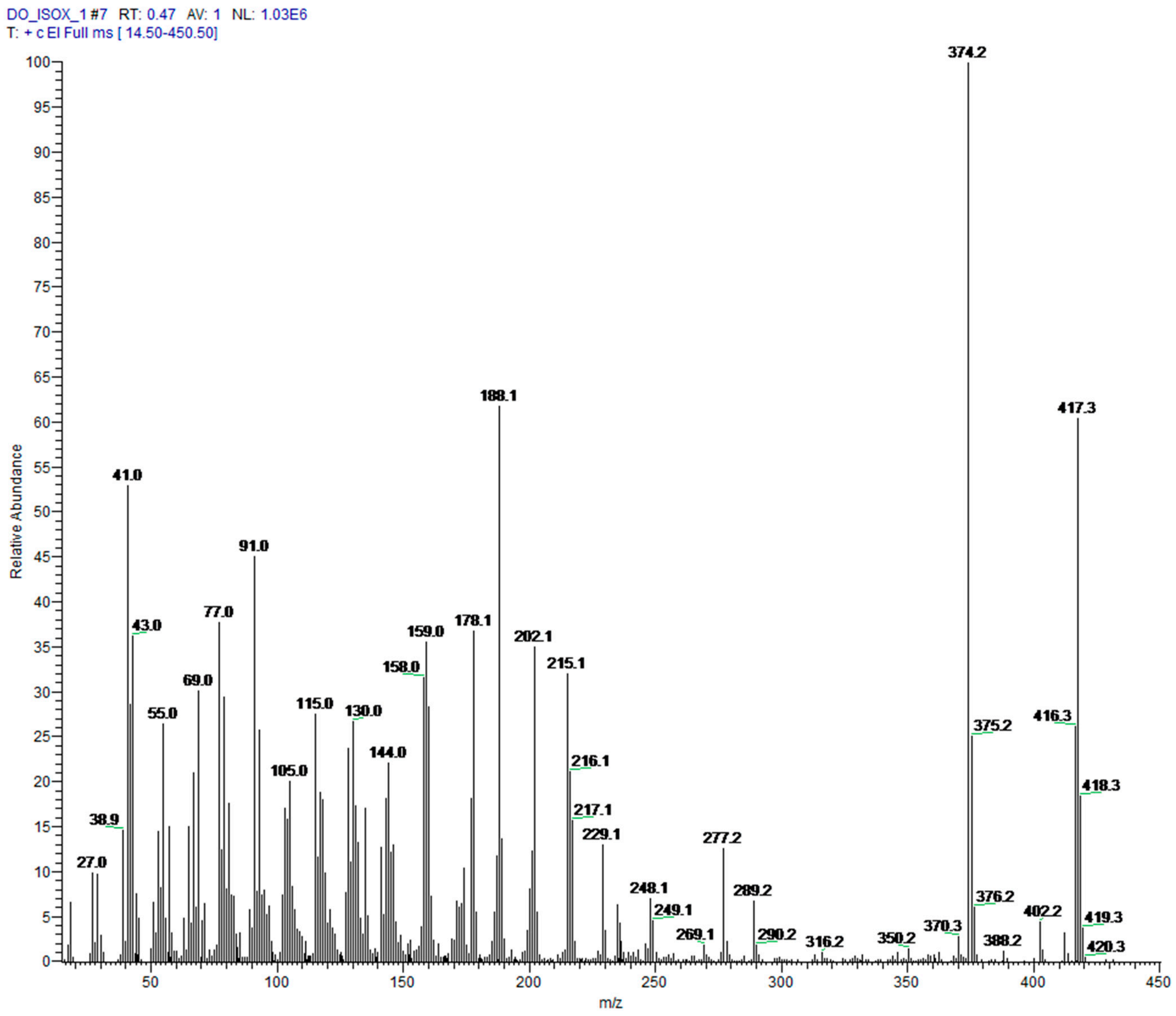

Figure S35. HRMS spectra of Compound **14b**

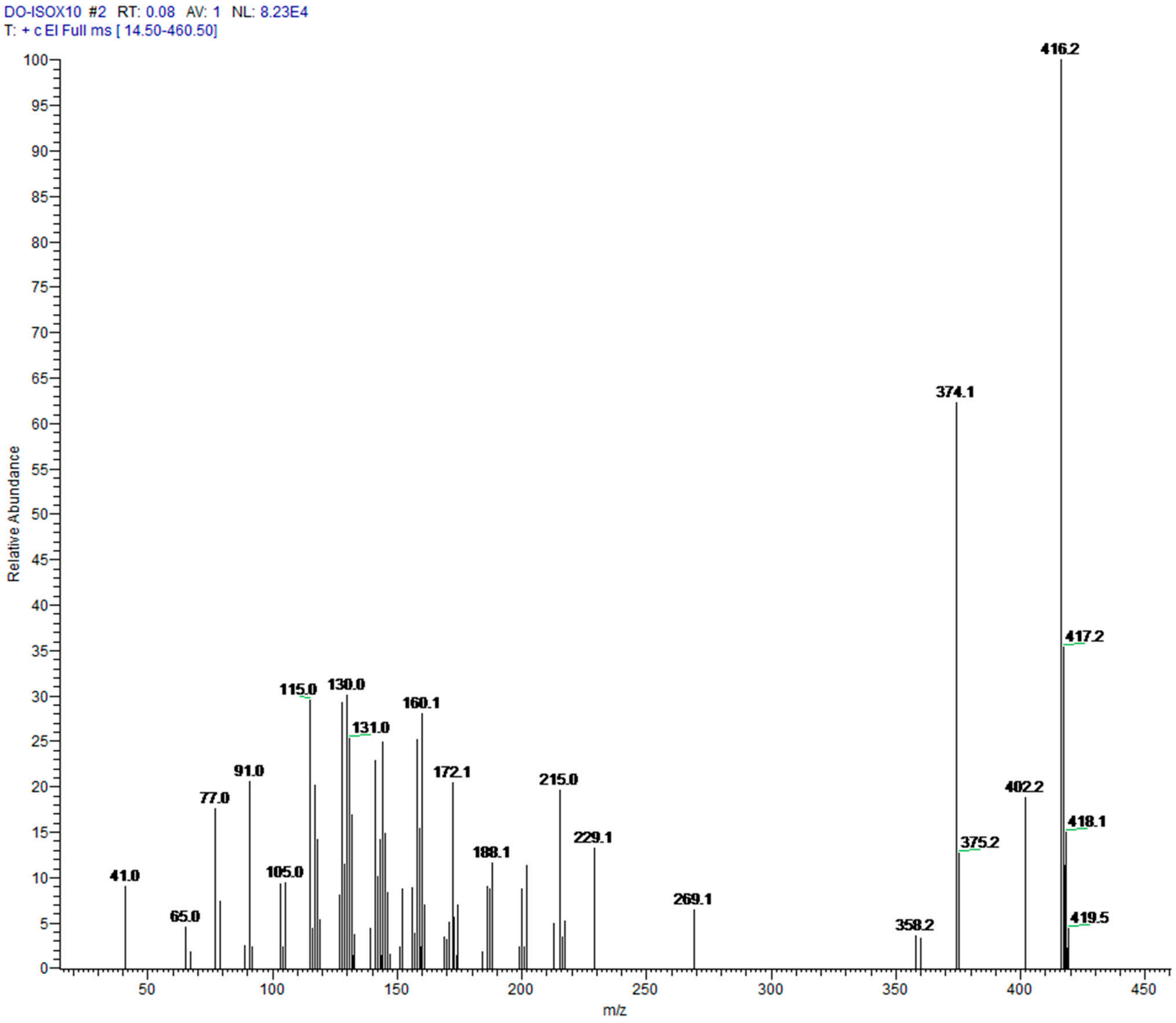

Figure S36. HRMS spectra of Compound **14c**

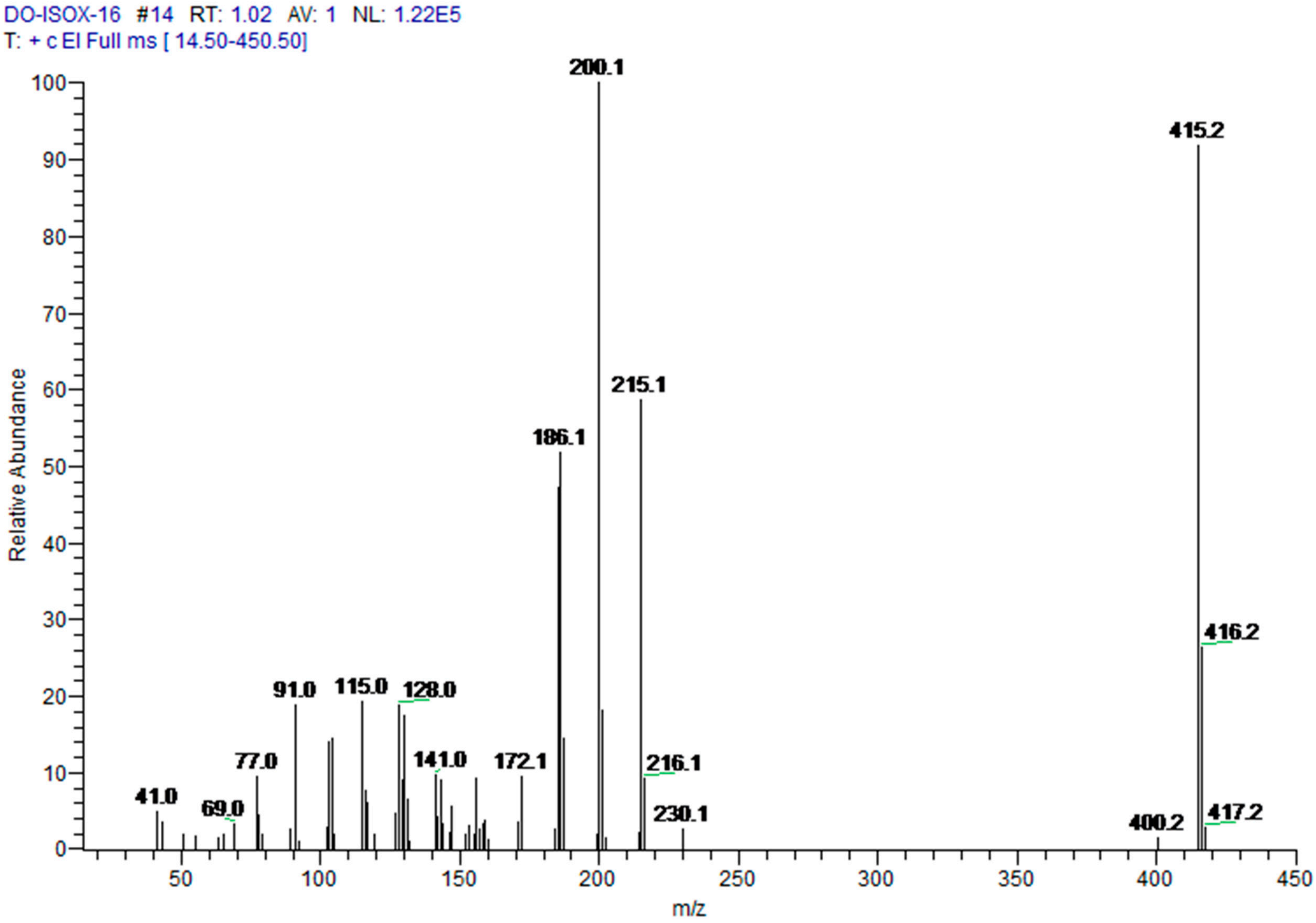

Figure S37. HRMS spectra of Compound **15a**

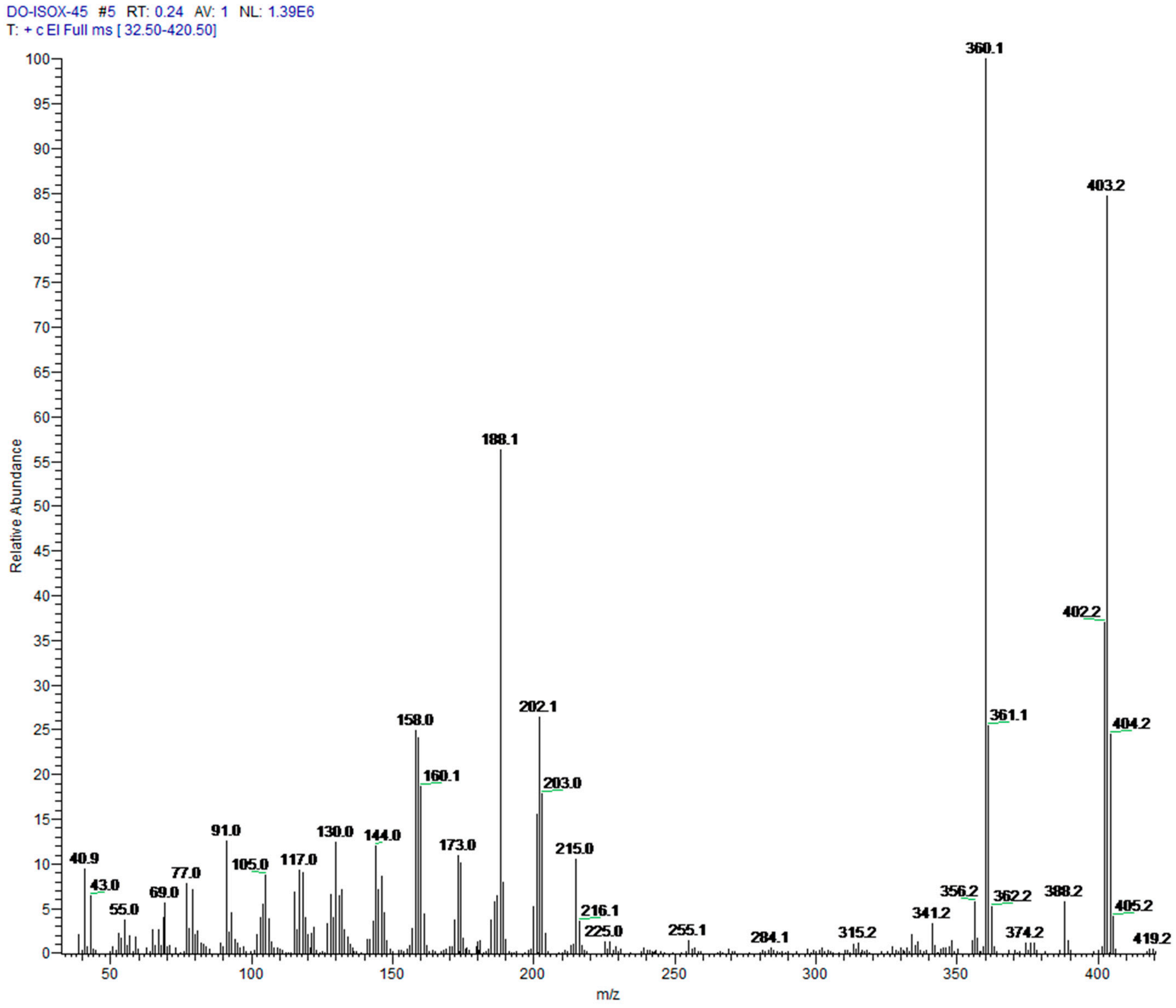

Figure S38. HRMS spectra of Compound **15b**

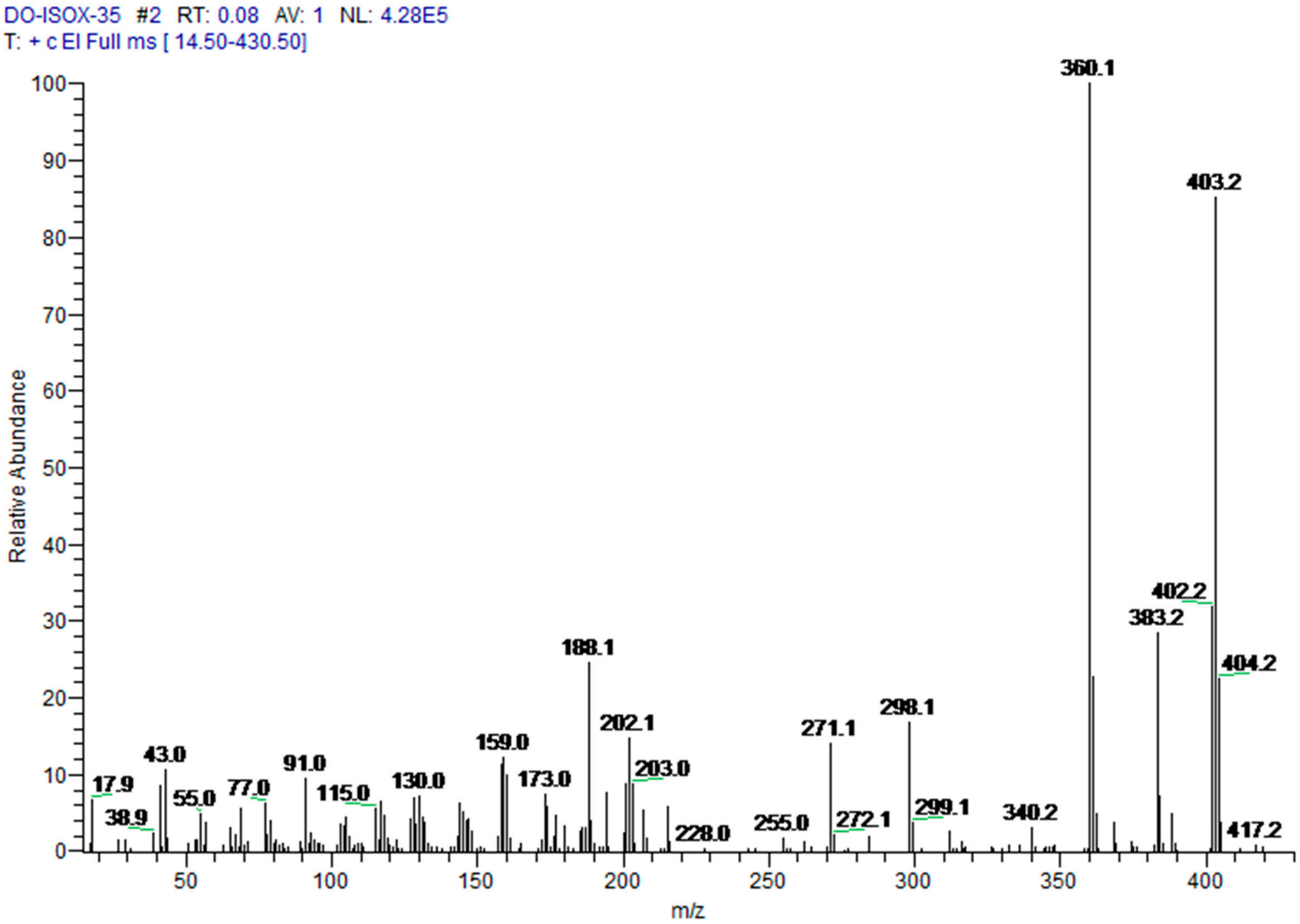

Figure S39. HRMS spectra of Compound **15c**

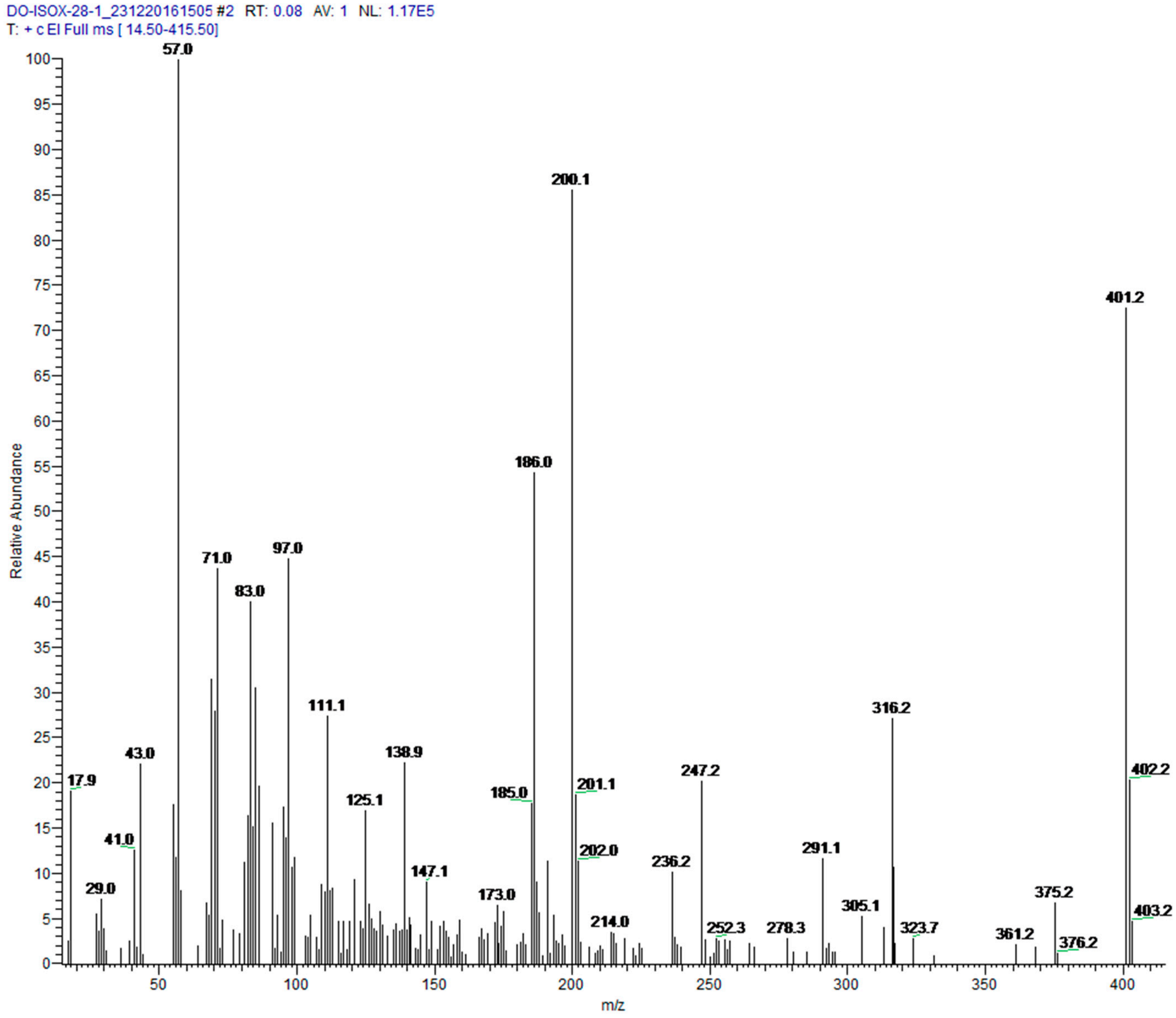

Figure S40. HRMS spectra of Compound **16a**

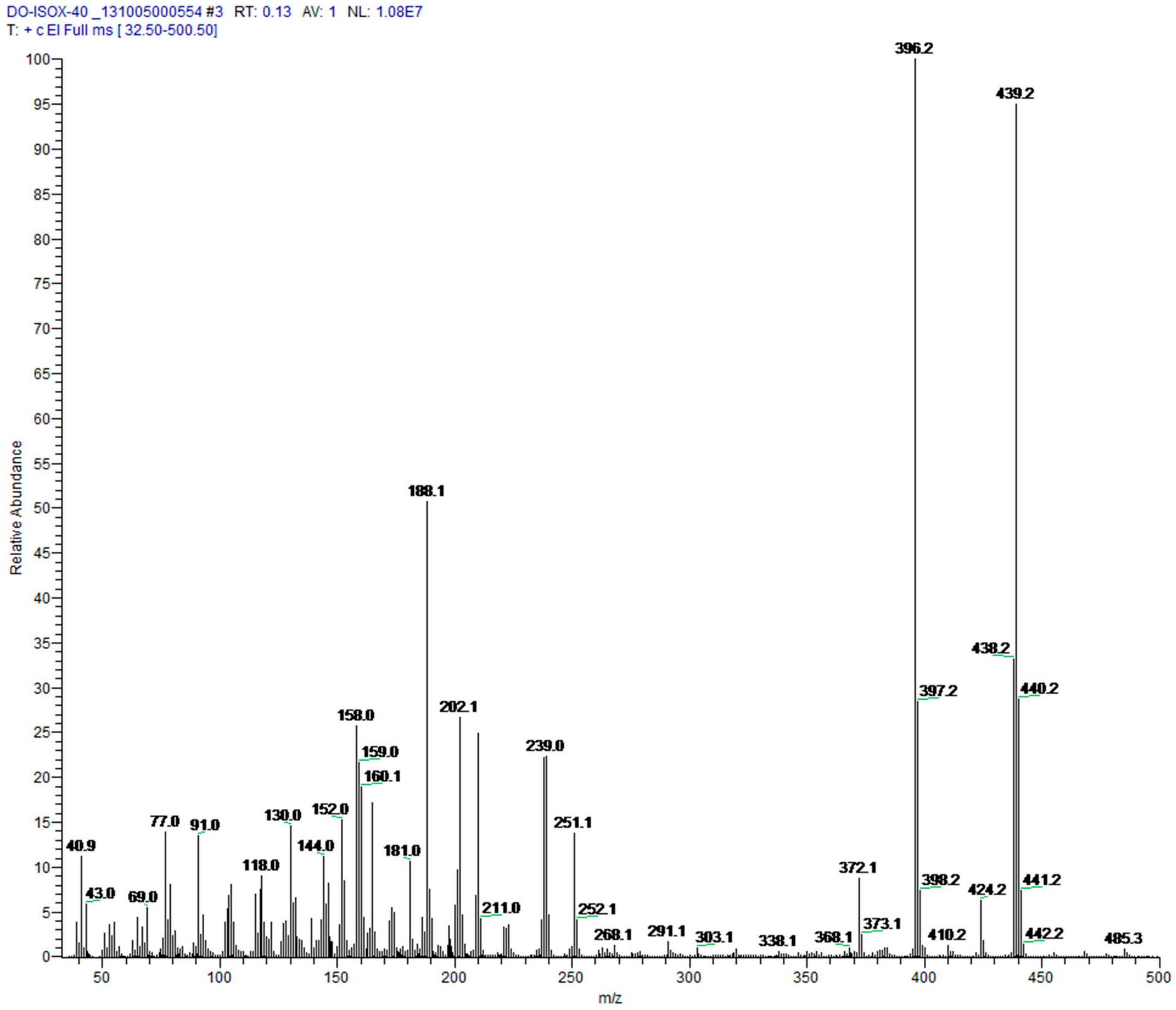

Figure S41. HRMS spectra of Compound **16b**

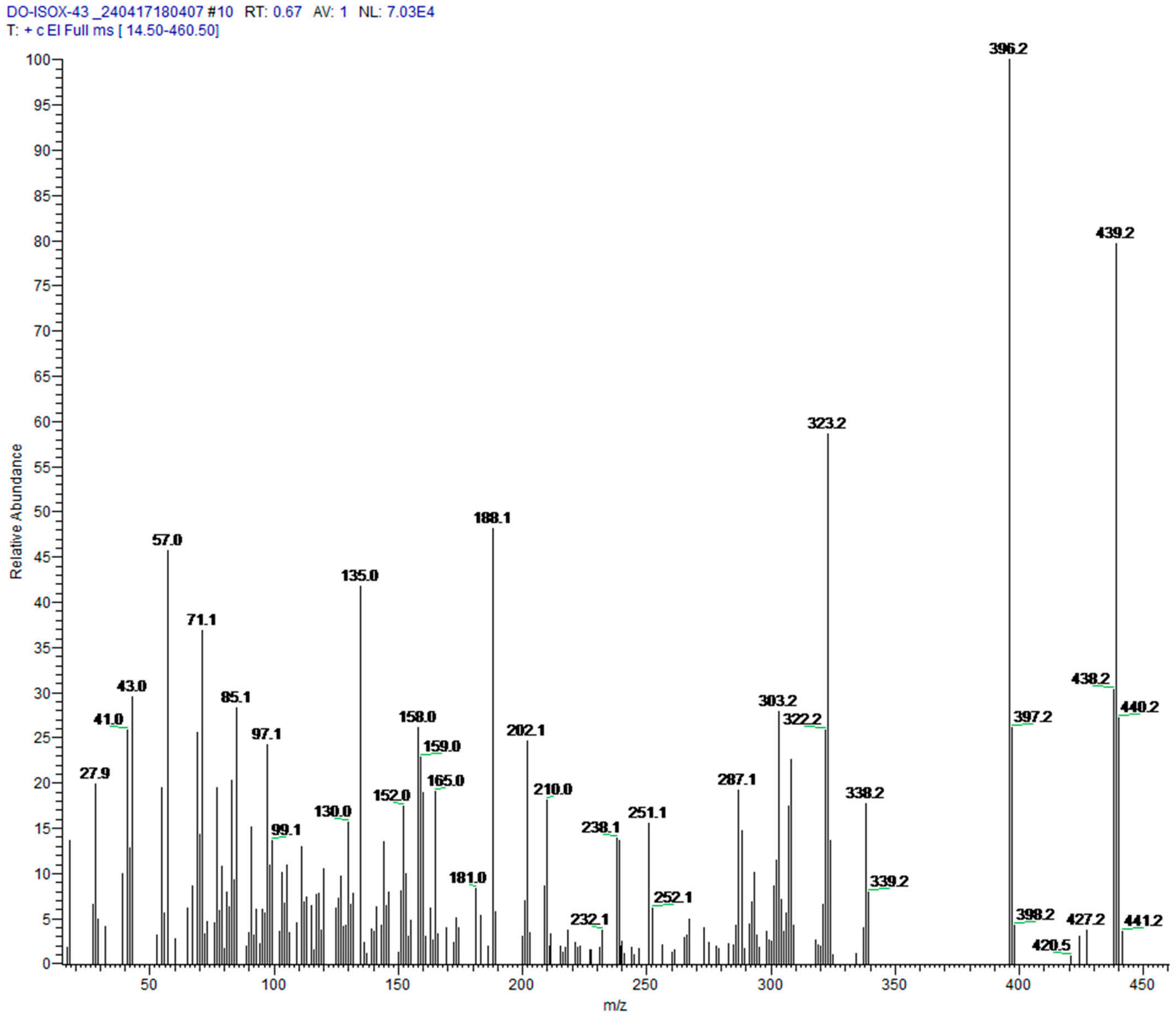

Figure S42. HRMS spectra of Compound **16c**

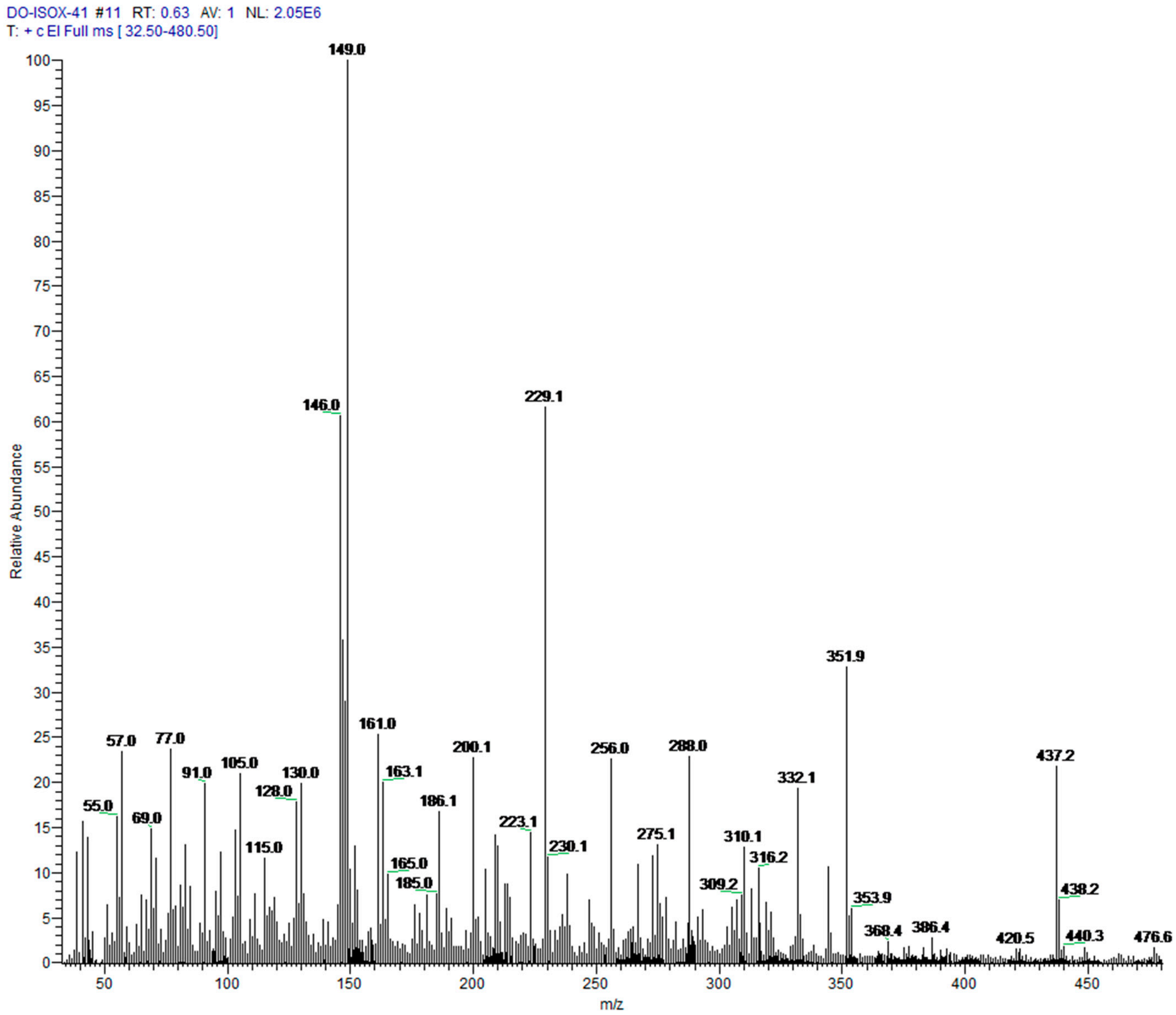

Figure S43. HRMS spectra of Compound **17a**

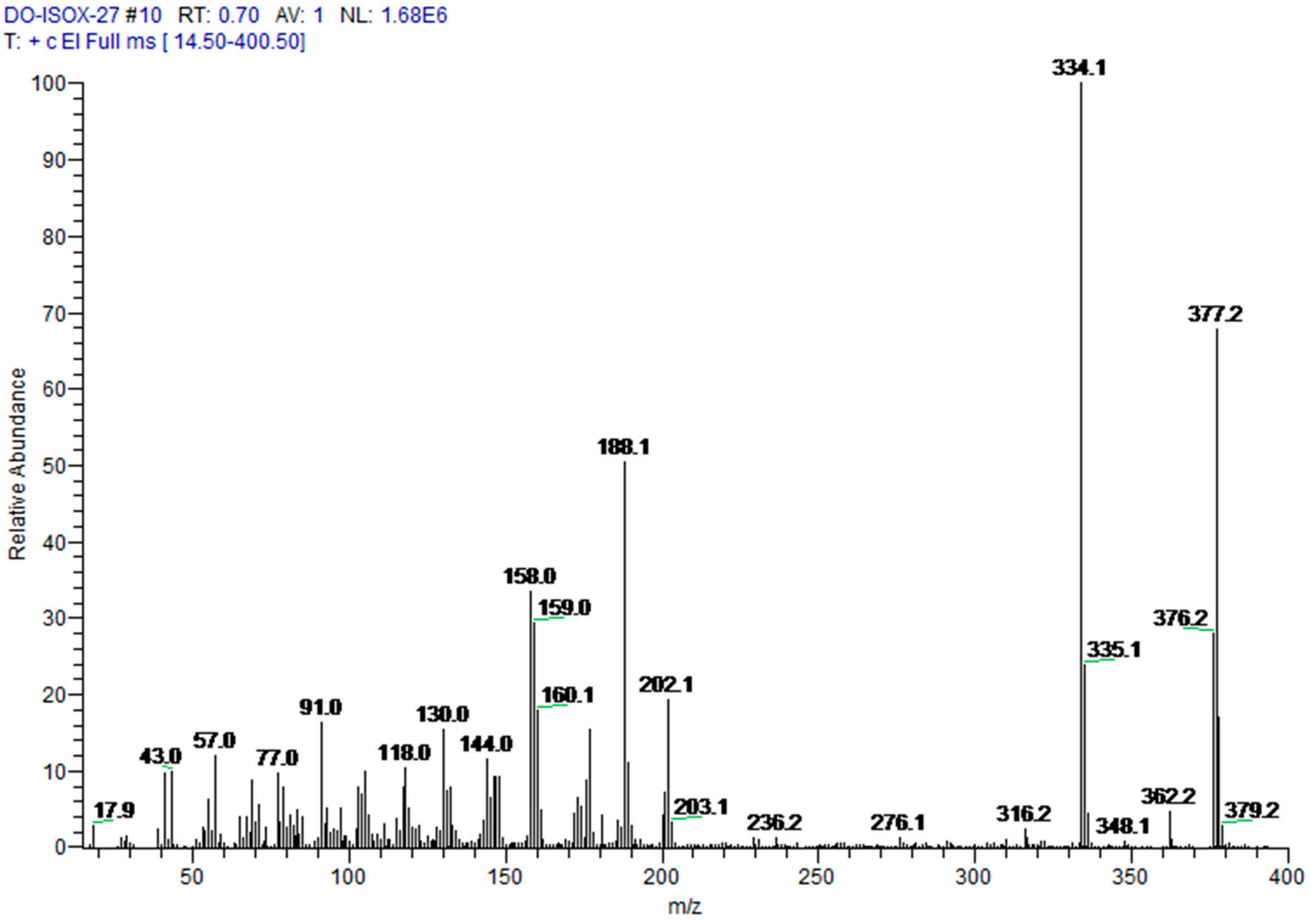

Figure S44. HRMS spectra of Compound **17b**

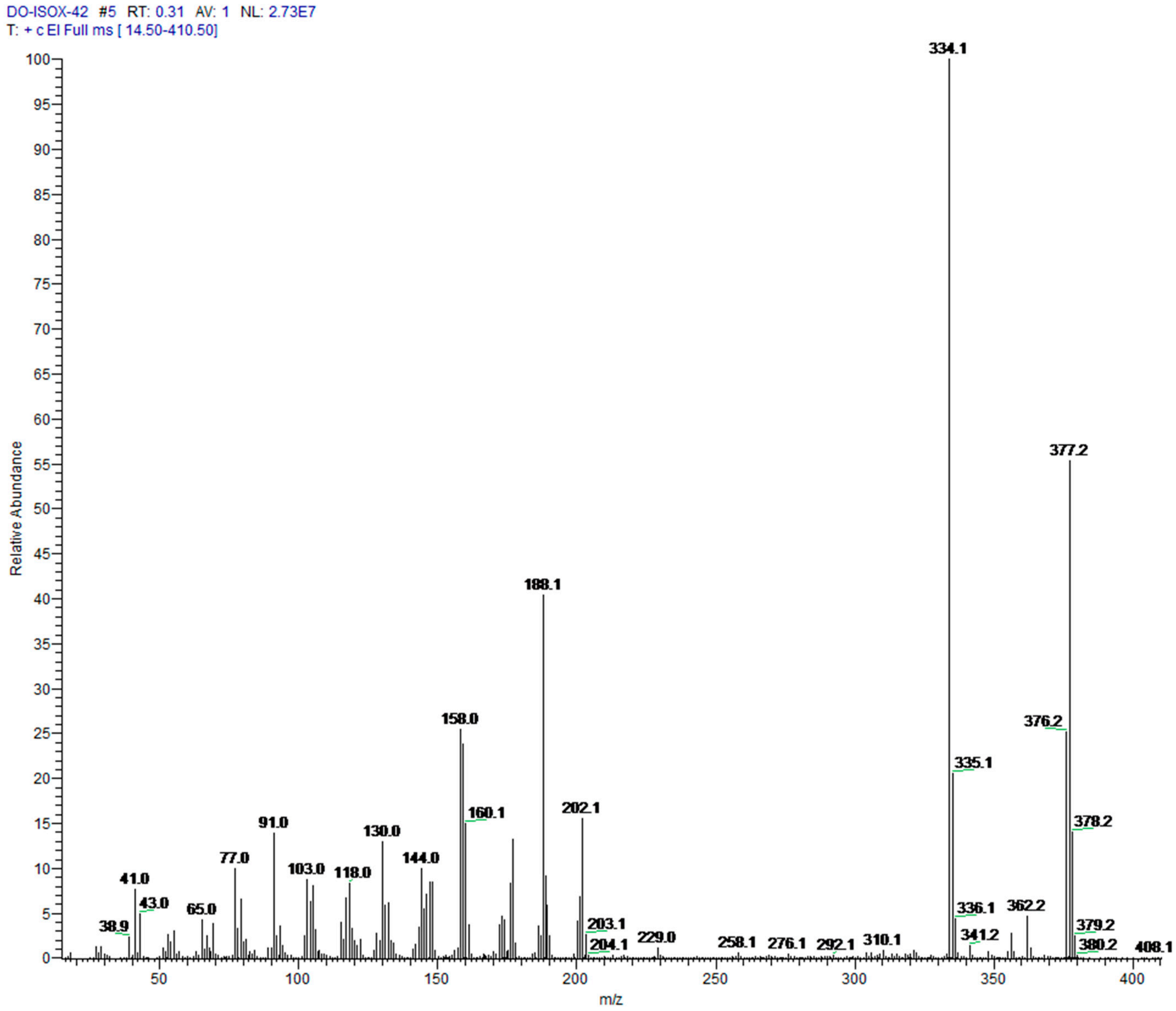

Figure S45. HRMS spectra of Compound **17c**

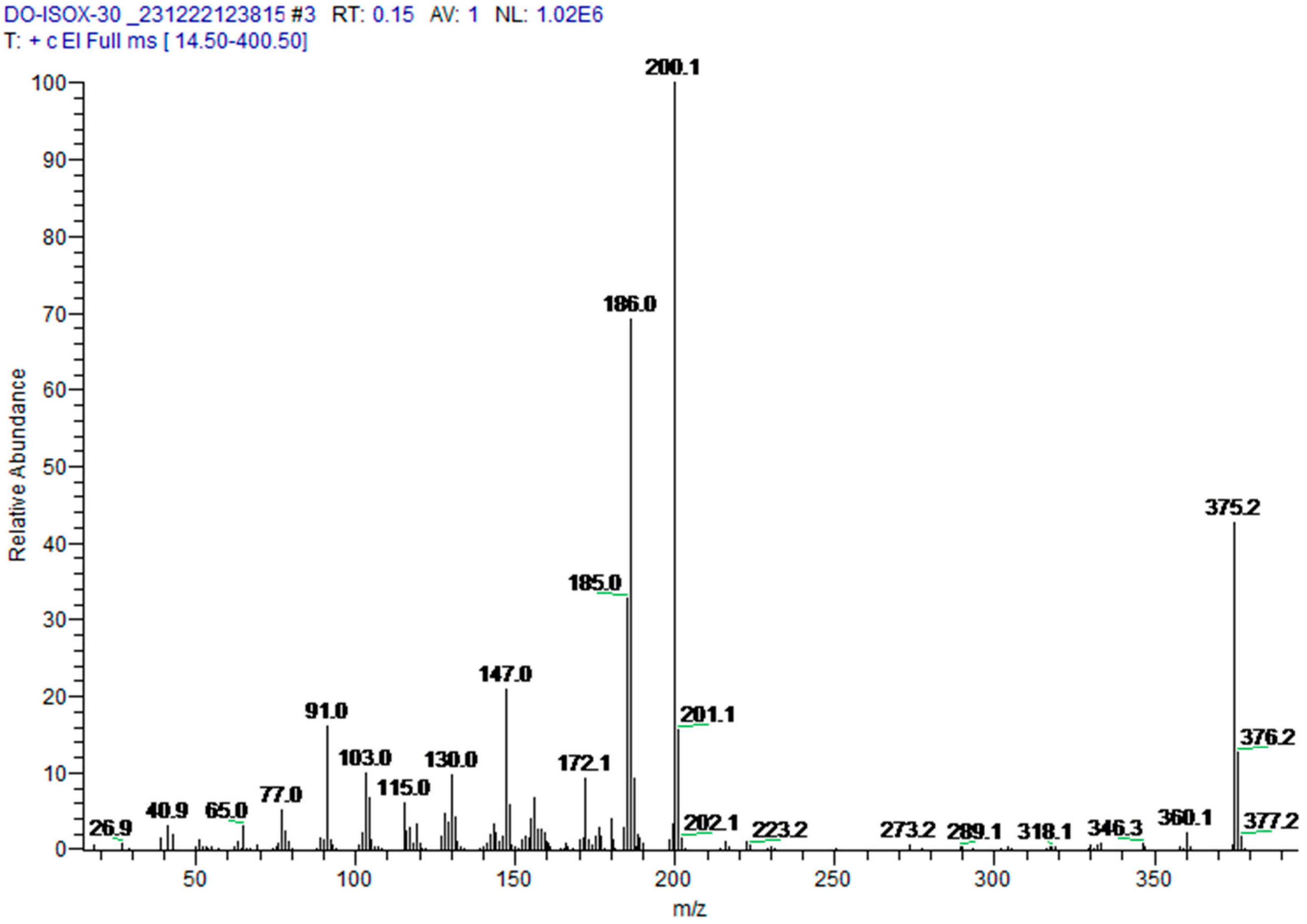

Figure S46. HRMS spectra of Compound **13e**

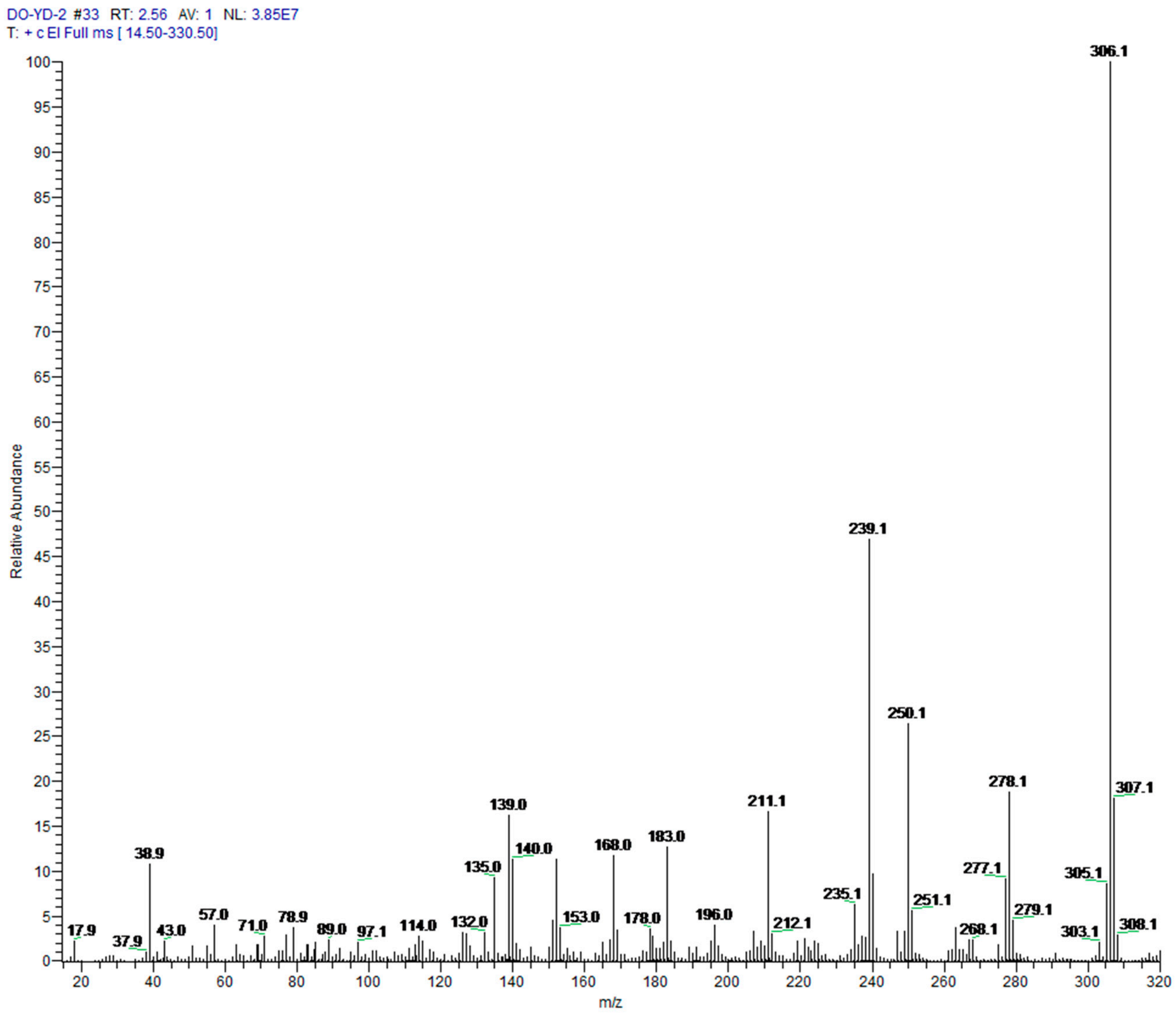

3. ^{1}H NMR spectra (ppm from 8.4 to 5.3) of the 12b dimerization recorded with a 3-minute delay at 200 MHz resolution.

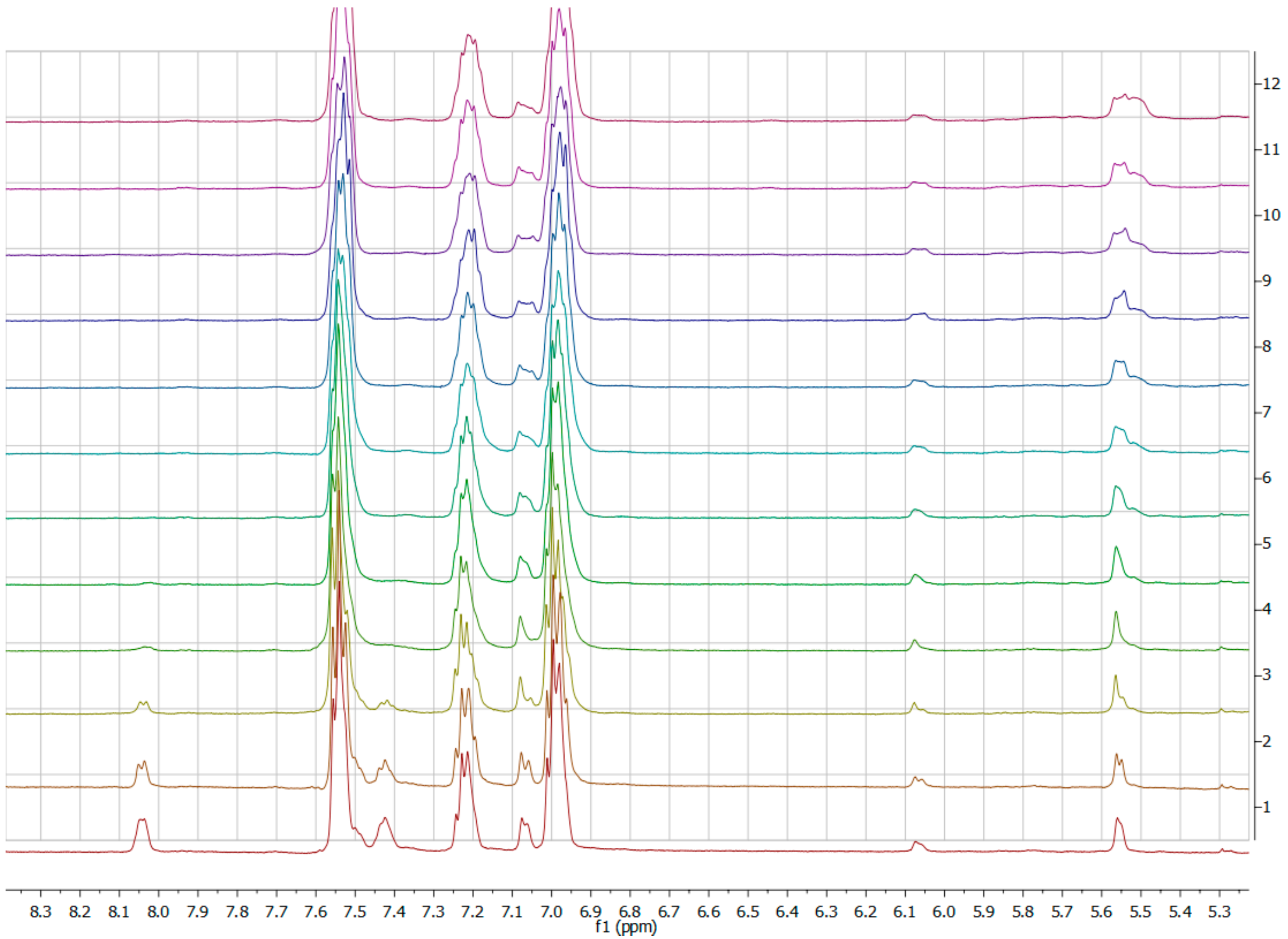

Figure S47. ^{1}H NMR spectra (ppm from 8.4 to 5.3) of the 12b dimerization recorded with a 3-minute delay at 200 MHz resolution. Different spectra are color-coded. The spectra are ordered by time from bottom to top.

4. Crystallographic structures of human TDP1 used in this study and validation of the docking process

Table S1. Crystallographic structures of human TDP1 used in this study and the RMSD value from redocking of each co-crystallized inhibitor

PDB code [a]	Reference	Resolution (Å)	Docking coordinates	RMSD (Å)
6dhu	[63]	1.63	7.62, -15.18, -33.97	4.8215
6dih	[63]	1.78	-7.34, -14.90, 34.17	2.9272
6dim	[63]	1.81	-7.09, -14.06, 32.14	0.6748
6dje	[63]	1.71	6.58, -15.25, -31.73	0.2997
6djf	[63]	1.67	-7.04, -14.41, 31.73	5.0333
6djg	[63]	1.88	-6.88, -14.66, 31.79	6.3284
6djh	[63]	1.92	-7.19, -13.94, 31.92	2.7436
6dji	[63]	1.75	7.34, -14.71, -34.22	0.6672
6djj	[63]	1.74	-6.98, -15.08, 33.92	2.4953
6mj5	[63]	1.85	-6.97, -14.43, 31.78	4.4558
6myz	[80]	1.66	6.44, -15.40, -31.57	2.0363
6mz0	[81]	1.97	7.70, -14.39, -33.05	2.5415
6n0d	[82]	1.45	7.57, -14.59, -34.09	0.3332
6n0n	[83]	1.48	8.02, 2.07, 0.61	2.7065
6n0o	[84]	1.94	-7.43, -14.79, 34.19	0.5436
6n0r	[85]	1.54	7.60, -15.88, -34.32	1.2845

6n17	[63]	1.64	7.67, -17.19, -34.15	1.4467
6n19	[63]	1.50	7.64, -17.75, -34.59	1.9401
6w4r	[86]	1.82	9.71, -3.29, -99.98	5.5867
6w7j	[87]	1.49	5.82, -16.44, -32.17	7.1206
6w7k	[22]	1.70	6.00, -16.76, -32.35	5.8508
6w7l	[22]	1.86	5.86, -16.26, -32.32	4.5798
7ufy	[23]	1.58	9.80, -4.49, -102.55	8.2684
8cvq	[21]	1.65	5.48, -18.01, -32.42	5.532
8cw2	[21]	1.81	4.50, -15.41, -30.13	6.1631
8uzv	[88]	1.85	6.52, -16.06, -31.33	0.7999
8v0b	[89]	1.65	6.25, -16.76, -31.11	3.3518

[a] PDB structures highlighted in yellow were selected for evaluation due to low RMSD values

Table S2. R^2 values between the IC_{50} and docking scores (kcal/mol) or ΔG (kcal/mol) for the title compounds with each PDB structure

PDB ID	Score	R^2
6dim	Docking score	0.0383
	ΔG	0.0571
6dje	Docking score	0.1083
	ΔG	0.1723
6dji	Docking score	0.0031
	ΔG	0.0413
6n0d	Docking score	0.0231
	ΔG	0.0918
6n0o	Docking score	0.0079
	ΔG	0.0997
8uzv	Docking score	0.0080
	ΔG	0.1595

Table S3. Docking scores (kcal/mol) and ΔG values (kcal/mol)

	6dim		6dje		6dji	
**Compound**	**Docking score**	**ΔG**	**Docking score**	**ΔG**	**Docking score**	**ΔG**
**14a**	-4.0	-58.0	-1.9	-52.5	-3.2	-46.6
**14b**	-2.4	-51.1	-3.9	-61.8	-3.5	-45.2
**14c**	-4.4	-59.2	-2.5	-68.1	-2.3	-57.1
**14d**	-3.4	-38.8	-3.5	-53.2	-1.5	-45.3
**15a**	-3.4	-51.8	-4.1	-63.7	-3.1	-55.2
**15b**	-2.1	-53.4	-4.1	-48.5	-3.3	-51.3
**15c**	-4.0	-48.7	-4.3	-51.9	-3.8	-46.9
**15d**	-2.4	-37.0	-3.1	-55.1	-2.9	-38.6
**16a**	-3.2	-50.4	-3.8	-56.9	-3.0	-56.1
**16b**	-3.5	-41.5	-1.6	-48.6	-2.5	-61.0
**16c**	-1.7	-59.0	-2.3	-51.6	-2.9	-60.1
**16d**	-3.3	-47.0	-2.3	-54.4	-0.9	-51.9
**17a**	-3.9	-45.3	-3.5	-40.3	-3.8	-40.9
**17b**	-3.8	-39.2	-3.4	-53.3	-3.7	-43.1
**17c**	-3.2	-51.9	-3.8	-46.9	-3.9	-47.7
**17d**	-3.9	-48.5	-3.5	-46.9	-2.5	-41.7

**22a**	**-3.9**	**-57.7**	**-4.0**	**-73.1**	**-2.7**	**-60.4**
**22b**	**-4.3**	**-56.3**	**-3.5**	**-74.8**	**-1.7**	**-58.6**
**22c**	**-0.3**	**-51.4**	**-3.6**	**-73.3**	**-2.9**	**-67.4**
**22d-*E***	**-2.3**	**-42.8**	**-0.4**	**-49.2**	**-2.5**	**-60.5**
**22d-*Z***	**-2.6**	**-58.8**	**-0.4**	**-47.5**	**-3.3**	**-59.4**
**22e**	**-0.5**	**-58.0**	**-2.7**	**-63.2**	**-1.3**	**-52.9**
**23a**	**-3.9**	**-52.3**	**-4.0**	**-63.5**	**-2.6**	**-44.7**
**23b**	**-3.7**	**-52.8**	**-3.0**	**-46.9**	**-3.1**	**-47.1**
**23c**	**-2.7**	**-56.8**	**-3.4**	**-55.1**	**-2.1**	**-62.9**
**23d**	**-1.8**	**-44.9**	**-3.4**	**-60.1**	**-2.7**	**-62.1**
**23e**	**1.4**	**-34.6**	**-3.4**	**-55.2**	**-2.7**	**-50.8**
**24a**	**-3.6**	**-41.5**	**-3.9**	**-60.7**	**-3.2**	**-53.0**
**24b**	**-3.1**	**-47.3**	**-4.1**	**-66.0**	**-2.7**	**-46.9**
**24c**	**-3.4**	**-45.2**	**-4.8**	**-64.9**	**-3.5**	**-49.0**
**24d**	**-3.1**	**-42.2**	**-2.3**	**-54.1**	**-3.0**	**-42.6**
**24e**	**-3.9**	**-45.2**	**-2.8**	**-56.4**	**-2.1**	**-48.8**
**25a**	**-3.9**	**-61.9**	**-3.9**	**-68.6**	**-2.3**	**-55.0**
**25b**	**-3.8**	**-47.7**	**-1.9**	**-47.5**	**-3.1**	**-61.1**
**25c**	**-3.4**	**-58.7**	**-3.3**	**-74.3**	**-3.5**	**-63.9**

25d	-2.3	-50.0	-3.2	-67.5	-3.5	-56.7
25e	-3.6	-56.4	-2.9	-67.2	-2.6	-56.6
26a	-4.1	-43.3	-0.1	-29.2	-2.5	-48.7
26b	-3.6	-41.6	-3.3	-53.8	-2.8	-46.7
26c	-4.1	-42.6	-1.4	-37.9	-3.6	-52.9
26d-*E*	-3.4	-41.4	-0.8	-54.8	-0.6	-47.3
26d-*Z*	-1.4	-49.2	-1.6	-42.9	-2.8	-34.3
26e	-2.9	-44.0	-3.5	-41.0	-2.8	-42.5

Table S3. ***Continued…***

	6n0d		6n0o		8uzv	
**Compound**	**Docking score**	**ΔG**	**Docking score**	**ΔG**	**Docking score**	**ΔG**
14a	-3.5	-50.5	-3.6	-43.8	-2.2	-44.6
14b	-4.0	-52.7	-3.6	-45.8	-2.8	-55.7
14c	-2.4	-63.2	-3.1	-46.7	-4.8	-60.4
14d	-3.3	-52.2	-3.9	-53.9	-0.2	-63.9
15a	-3.4	-48.8	-3.9	-37.4	-3.4	-50.1
15b	-1.1	-50.5	-2.7	-48.7	-3.1	-55.7
15c	-2.9	-51.1	-2.9	-48.4	-3.3	-59.0
15d	-2.7	-42.1	-2.5	-49.5	-2.9	-60.5

**16a**	**-3.4**	**-53.7**	**-3.1**	**-49.6**	**-3.4**	**-64.1**
**16b**	**-4.1**	**-58.5**	**-4.1**	**-52.4**	**-1.8**	**-68.4**
**16c**	**-2.4**	**-57.5**	**-3.1**	**-54.2**	**-0.1**	**-72.5**
**16d**	**-2.6**	**-57.9**	**-2.2**	**-45.1**	**-2.3**	**-71.2**
**17a**	**-2.0**	**-54.8**	**-3.6**	**-53.2**	**-3.6**	**-44.4**
**17b**	**-3.4**	**-49.8**	**-2.7**	**-53.6**	**-3.3**	**-59.7**
**17c**	**-0.6**	**-55.4**	**-3.7**	**-52.6**	**-2.4**	**-64.4**
**17d**	**-2.5**	**-51.9**	**-4.2**	**-36.2**	**-3.8**	**-57.8**
**22a**	**-4.9**	**-52.9**	**-3.8**	**-54.9**	**-3.0**	**-63.0**
**22b**	**-0.2**	**-54.9**	**-3.2**	**-51.4**	**-3.9**	**-48.8**
**22c**	**-1.3**	**-52.7**	**-2.8**	**-63.0**	**-0.3**	**-65.0**
**22d-*E***	**-1.5**	**-49.0**	**-4.5**	**-53.6**	**-1.0**	**-67.7**
**22d-*Z***	**-2.8**	**-59.5**	**-3.5**	**-55.5**	**-3.4**	**-39.1**
**22e**	**-3.2**	**-64.0**	**-3.2**	**-48.6**	**-0.2**	**-67.0**
**23a**	**-2.7**	**-49.0**	**-3.7**	**-39.4**	**-3.2**	**-51.8**
**23b**	**-2.2**	**-54.7**	**-3.6**	**-39.7**	**-0.3**	**-43.4**
**23c**	**-3.4**	**-57.5**	**-2.1**	**-52.9**	**-2.3**	**-59.8**
**23d**	**-2.7**	**-55.6**	**-2.7**	**-36.3**	**-3.5**	**-66.7**
**23e**	**-2.8**	**-59.2**	**-3.9**	**-46.8**	**-4.0**	**-60.1**

**24a**	**-3.5**	**-60.2**	**-3.1**	**-46.1**	**-3.3**	**-63.7**
**24b**	**-2.7**	**-47.9**	**-3.6**	**-37.6**	**-4.6**	**-62.9**
**24c**	**-3.1**	**-59.0**	**-2.4**	**-48.8**	**-2.5**	**-54.6**
**24d**	**-3.3**	**-64.2**	**-1.8**	**-49.0**	**-2.2**	**-62.3**
**24e**	**-3.1**	**-54.8**	**-2.4**	**-48.8**	**-4.3**	**-64.5**
**25a**	**-4.2**	**-54.0**	**-4.0**	**-60.5**	**-4.0**	**-58.7**
**25b**	**-1.1**	**-58.9**	**-3.3**	**-56.2**	**-3.3**	**-51.4**
**25c**	**-2.6**	**-57.1**	**-2.0**	**-58.2**	**-0.8**	**-52.2**
**25d**	**-2.0**	**-45.9**	**-3.5**	**-56.8**	**-2.0**	**-74.3**
**25e**	**-2.8**	**-46.2**	**-2.7**	**-56.9**	**-0.4**	**-68.1**
**26a**	**-3.4**	**-46.0**	**-3.5**	**-39.4**	**-2.7**	**-44.1**
**26b**	**-3.8**	**-46.5**	**-3.8**	**-41.1**	**-2.5**	**-39.9**
**26c**	**-3.3**	**-51.3**	**-2.7**	**-49.7**	**-2.6**	**-46.4**
**26d-*E***	**-1.0**	**-45.9**	**-3.4**	**-41.4**	**-3.9**	**-55.0**
**26d-*Z***	**-2.5**	**-40.1**	**-3.2**	**-50.5**	**-3.4**	**-54.6**
**26e**	**-1.5**	**-51.7**	**-2.3**	**-42.1**	**-3.6**	**-46.0**

5. Prediction of druglikeness

Figure S48. Bioavailability radars of the compounds. Pink area of each radar represents orally available druglike chemical space

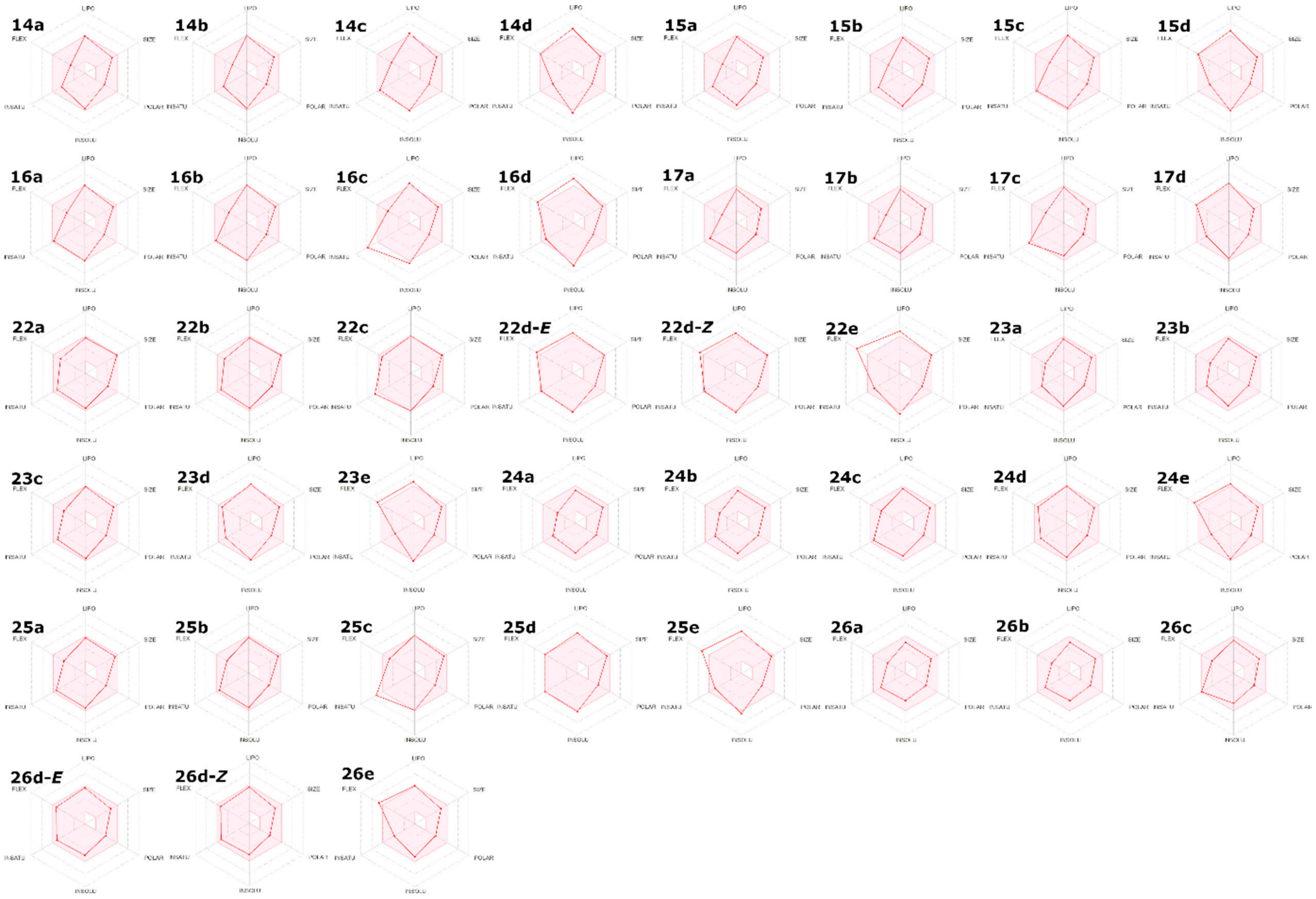

6. Electrostatic and van der Waals interactions of the compounds with TDP1 as predicted through molecular docking

Figure S49. 2D interaction diagrams for the predicted binding of 14b (A), 14d (B), 15a (C), 16a (D), 16b (E), and 16c (F) in human TDP1 active site. Electrostatic interactions are indicated by color-coded lines(magenta for H bond, green for π–π, and red for π–cation).

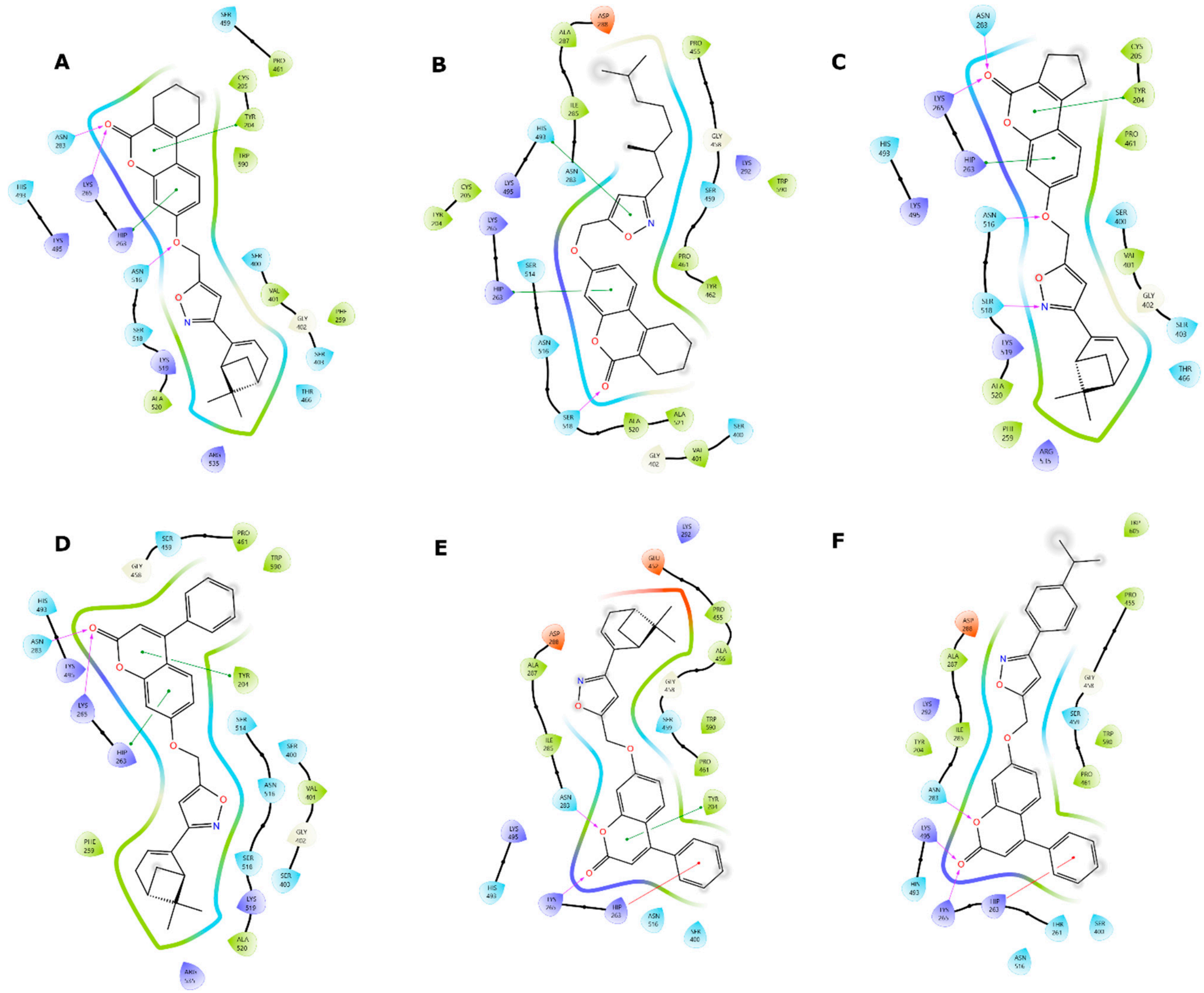

Figure S50. 2D interaction diagrams for the predicted binding of 22a (A), 22b (B), 22c (C), 23a (D), 23b (E), and 23c (F) in human TDP1 active site. Electrostatic interactions are indicated by color-coded lines(magenta for H bond, green for π–π, and red for π–cation).

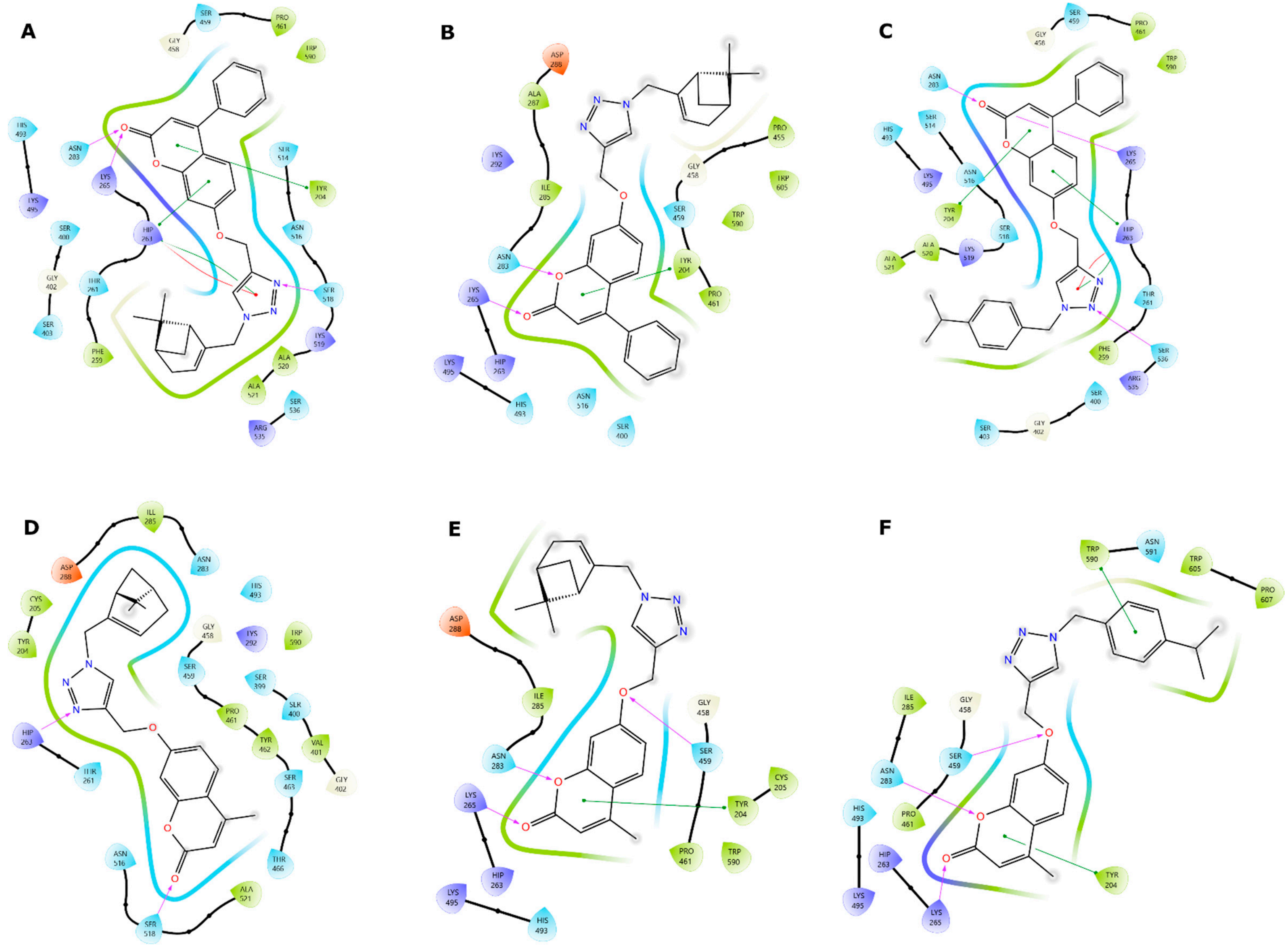

Table S4. TDP1 residues involved in electrostatic and van der Waals interactions with selected compounds according to molecular docking with 6DJE

Comp.	H-bond	π–π/π–cation	Hydrophobic
14b	Asn283, Lys265, Asn516	Tyr204, His263	Cys205, Phe259, Val401, Ala520, Trp590
14d	Ser518	His263, His493	Tyr204, Ile285, Ala287, Pro455, Pro461, Tyr462, Ala520, Ala521
15a	Lys265, Asn283, Asn516, Ser518	Tyr204, His263	Phe259, Val401, Pro461, Ala520
16a	Lys265, Asn283	Tyr204, His263	Phe259, Val401, Pro461, Ala520, Trp590
16b	Lys265, Asn283	Tyr204, His263 (π-cation)	Ile285, Ala287, Pro455, Ala456, Pro461, Trp590
16c	Lys265, Asn283, Lys495	His263 (π-cation)	Tyr204, Ile285, Ala287, Pro461, Pro455, Trp590, Trp605
22a	Lys265, Asn283, Ser518	Tyr204, His263 (π-cation)	Phe259, Pro455, Pro461, Ala520, Ala521, Trp590
22b	Lys265, Asn283	Tyr204	Ile285, Ala287, Pro455, Pro461, Trp590, Trp605
22c	Lys265, Asn283, Ser536	Tyr204, His263 (π-cation)	Phe259, Pro461, Ala520, Ala521, Trp590
23a	His263, Ser518	-	Tyr204, Ile285, Val401, Pro461, Tyr462, Ala521, Trp590
23b	Lys265, Asn283, Ser459	Tyr204	Ile285, Pro461, Trp590
23c	Lys265, Asn283, Ser459	Tyr204, Trp590	Ile285, Pro461, Trp605, Pro607

7. HPLC Retention times of compounds varying in the heterocyclic linker only.

Figure S51. HPLC Retention times of compounds 23b, 14b, SI1; 26b, 17b, SI2; 24a, 15a, SI3.

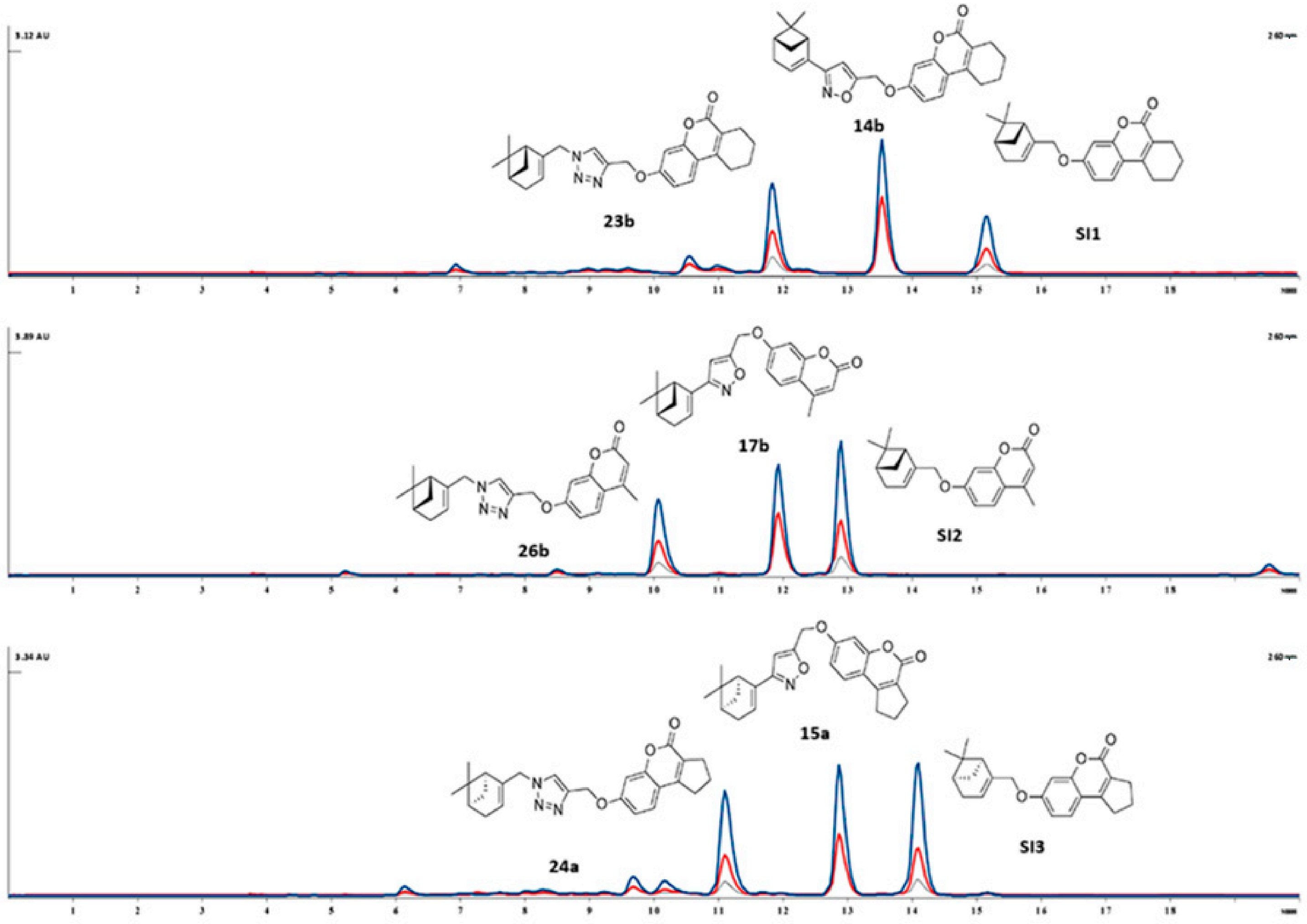

Supplement: Supplementary file 1 [file ijms-27-06421-s001.zip › ijms-4426852-supplementary.pdf]
